# P2X7 receptors promote atrial remodeling and atrial fibrillation susceptibility via reactive oxygen species‐mediated mitogen‐activated protein kinase signaling activation

**DOI:** 10.1002/ccs3.70071

**Published:** 2026-04-30

**Authors:** Lingnan Zhang, Yeran Zhu, Xinshun Gu

**Affiliations:** ^1^ Department of Cardiovascular The Second Hospital of Hebei Medical University Shijiazhuang China; ^2^ Department of Cardiovascular Affiliated Hospital of Hebei University Baoding China

**Keywords:** atrial fibrillation, atrial remodeling, inflammatory response, mitogen‐activated protein kinase signaling, P2X purinoceptor 7 receptor, reactive oxygen species

## Abstract

Atrial fibrillation (AF), the most common clinical arrhythmia, is driven by inflammatory activation and oxidative stress, though precise molecular links remain unclear. This study identifies the P2X7 receptor as a key upstream regulator orchestrating proarrhythmic atrial remodeling through reactive oxygen species (ROS)‐mediated mitogen‐activated protein kinase (MAPK) signaling. Transcriptomic analysis of rapid‐paced cardiomyocytes revealed P2X7 upregulation and MAPK pathway enrichment. Functional validation demonstrated that P2X7 activation promotes ROS accumulation, MAPK phosphorylation (p‐ERK, p‐p38, and p‐JNK), and pro‐inflammatory cytokine release (IL‐6 and IL‐1β), culminating in action potential shortening and calcium handling dysfunction. Critically, both P2X7 inhibition (A‐438079) and ROS scavenging (NAC) attenuated this signaling axis. In vivo, P2X7 antagonism reduced AF susceptibility, improved conduction heterogeneity, and ameliorated structural and autonomic remodeling. These results establish the P2X7‐ROS‐MAPK axis as a central mechanism in AF vulnerability and highlight its therapeutic potential.

## Graphical abstract

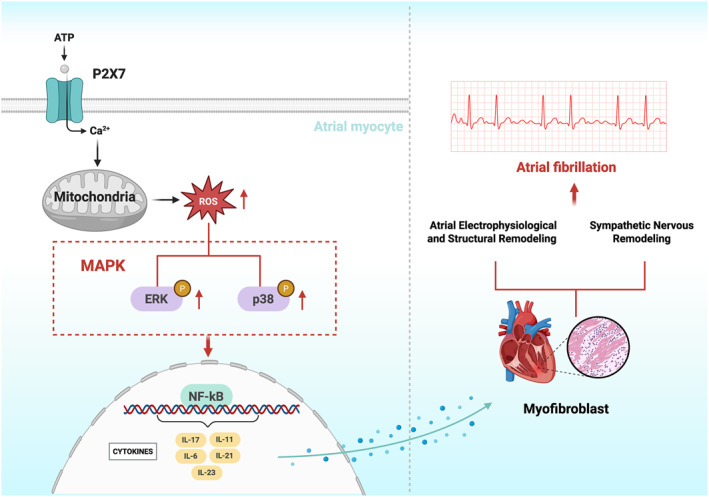

## 1 INTRODUCTION

Atrial fibrillation (AF) is among the most prevalent forms of persistent cardiac arrhythmia, with its incidence escalating sharply with advancing age. It has emerged as a major global public health concern in the context of cardiovascular disease.^1^, ^2^ Epidemiological data indicate that AF affects over 33 million individuals worldwide and significantly increases the risk of stroke, heart failure, and all‐cause mortality.^3^, ^4^ Despite the widespread clinical use of catheter ablation and anticoagulant therapy, recurrence rates remain high, and many patients exhibit suboptimal responses to or adverse effects in current pharmacological treatments.^5^, ^6^ These limitations underscore the urgent need to elucidate the molecular mechanisms underlying AF pathogenesis and to identify novel therapeutic targets.

Mounting evidence suggests that AF is not merely an electrical disorder but rather a multifactorial condition involving atrial structural remodeling, inflammation, and autonomic nervous system dysregulation.^7^ Among these, the interplay between electrical and structural remodeling in atrial cardiomyocytes constitutes a central pathological basis for the initiation and maintenance of AF, characterized by shortened action potential duration (APD), ion channel dysfunction, and interstitial fibrosis.^8^, ^9^ Oxidative stress and inflammatory responses have also been implicated as critical contributors to AF development, closely linked to myocardial injury and activation of pathogenic signaling cascades.^10^, ^11^ However, a comprehensive understanding of the cross talk among these pathological processes remains incomplete, thereby constraining the development of targeted therapeutic interventions. AF, as a typical supraventricular tachyarrhythmia, is characterized by disorganized electrical activity and impaired atrial contraction and conduction, often closely associated with atrial structural remodeling. Recent studies have identified oxidative stress as a key factor in cardiovascular pathology, with elevated levels of reactive oxygen species (ROS) contributing to atrial remodeling and increased susceptibility to AF.^12^ However, effective therapeutic strategies targeting ROS‐related signaling pathways remain limited, posing a major challenge in AF prevention and treatment.

The P2X purinoceptor 7 (P2X7) receptor, a member of the purinergic receptor family, functions as a ligand‐gated ion channel primarily activated by extracellular ATP. It plays a pivotal role in immune modulation, inflammatory signaling, and cell death.^13^, ^14^ Under physiological conditions, P2X7 expression is typically low; however, it can be markedly upregulated under pathological states such as inflammation, ischemia‐reperfusion injury, and tissue fibrosis.^15^, ^16^ Upon activation, P2X7 promotes membrane depolarization, calcium influx, and pore formation, triggering downstream signaling pathways including nuclear factor kappa B (NF‐κB), the NLRP3 inflammasome, and the mitogen‐activated protein kinase (MAPK) cascade.^17^, ^18^ Prior studies have implicated P2X7 in exacerbating inflammation and cellular injury in conditions such as myocardial infarction (MI), cardiac fibrosis, and hypertensive heart disease, with its inhibitor A‐438079 demonstrating cardioprotective effects in preclinical models.^19^, ^20^

Nonetheless, whether P2X7 is directly involved in AF pathogenesis and through what mechanisms remains poorly defined. Given the characteristic presence of ATP release, inflammatory activation, and oxidative stress in AF, P2X7 may represent a critical molecular nexus linking these pathological features.^13^, ^15^ Preliminary in vitro findings suggest that upregulation of P2X7 may contribute to ion channel dysregulation in cardiomyocytes; however, definitive evidence demonstrating its role in coordinating the inflammation oxidative stress‐signaling axis in AF is lacking.^13^, ^21^ Accordingly, further investigation is warranted to delineate the functional involvement of P2X7 in AF and to identify its downstream effector molecules.

Oxidative stress, a pathological condition arising from excessive production or insufficient clearance of ROS, is commonly implicated in cardiovascular diseases.^22^, ^23^ Accumulated ROS can directly impair cardiomyocytes, disrupt calcium handling, and promote apoptosis. Additionally, ROS function as signaling molecules that activate various downstream pathways.^24^, ^25^ In patients with AF, ROS levels are markedly elevated in atrial tissue, primarily due to NADPH oxidase isoforms (NOX2 and NOX4), mitochondrial leakage, and infiltration of inflammatory cells.^26^, ^27^ Among the affected signaling cascades, the MAPK pathway is particularly responsive to oxidative and inflammatory stimuli.^28^, ^29^ The MAPK family—comprising ERK, p38, and JNK subgroups—regulates apoptosis, pro‐inflammatory cytokine release, and matrix metalloproteinase expression, thereby contributing to atrial structural remodeling.^30^, ^31^ Previous studies have demonstrated that MAPK activation is pronounced in models of rapid atrial pacing and is closely associated with electrophysiological disturbances.^32^, ^33^ Moreover, ROS has been shown to enhance MAPK phosphorylation, indicating a potential signaling interplay.^34^, ^35^ However, whether this pathway is driven by upstream receptors such as P2X7 and its direct role in AF pathogenesis remain to be fully elucidated.^21^, ^36^ Elucidating the molecular origin and regulatory mechanisms of the ROS‐MAPK axis is therefore critical for developing targeted strategies to mitigate AF‐related remodeling.^37^, ^38^

Recent studies suggest that the P2X7 receptor not only facilitates ATP‐mediated inflammatory responses but may also modulate ROS levels, thereby influencing the activation of downstream signaling pathways. This positions P2X7 as a pivotal component of the P2X7‐ROS‐MAPK signaling axis.^34^ Activation of P2X7 has been shown to upregulate NOX2 and NOX4 expression, enhancing ROS production and indirectly promoting the phosphorylation of MAPK pathway components.^39^, ^40^ This axis is also closely linked to the expression of pro‐inflammatory cytokines such as interleukin‐1β (IL‐1β), interleukin‐6 (IL‐6), and tumor necrosis factor alpha (TNF‐α)—factors known to be elevated in both AF models and patients, and positively correlated with atrial conduction abnormalities and structural remodeling.^21^, ^41^ However, whether P2X7 drives MAPK activation via ROS accumulation to induce AF remains unverified.^15^ Moreover, there is a lack of integrative evidence from transcriptomic analyses to in vivo and in vitro functional validation, leaving the causal role of this signaling axis in AF onset and maintenance unresolved.^41^, ^42^ It also remains uncertain whether pharmacological inhibition of P2X7 or ROS scavenging can effectively attenuate MAPK activation and mitigate AF phenotypes.^43^ Thus, a comprehensive elucidation of the P2X7‐ROS‐MAPK axis may offer novel insights into the molecular mechanisms underlying AF and provide promising therapeutic targets.

This study aims to elucidate whether P2X7 promotes AF by mediating ROS accumulation and MAPK activation, thereby inducing inflammatory and remodeling responses. Using integrated bioinformatic, electrophysiological, and molecular approaches in vitro and in vivo, we systematically validate the P2X7‐ROS‐MAPK axis as a key mechanistic pathway and a therapeutic target in AF.

## 2 MATERIALS AND METHODS

### 2.1 Microarray data acquisition and preprocessing

This study utilized the GSE10598 dataset from the GEO database, which contains transcriptomic microarray data derived from HL‐1 murine atrial cardiomyocytes subjected to either rapid electrical stimulation or spontaneous beating conditions. Four microarray samples were analyzed in total: GSM267173 and GSM267175 as control samples and GSM267172 and GSM267174 as the stimulation group. Data acquisition was performed using the Affymetrix mouse genome 430 2.0 array platform. Raw probe signals were processed using the robust multi‐array average algorithm for background correction and normalization.

### 2.2 Principal component and differential expression analyses

To identify transcriptomic differences between experimental groups, differential expression analysis was conducted using the limma package in R. Genes were considered differentially expressed if they met the thresholds of |log_2_ fold change| > 1 and an adjusted *p*‐value (adj. p‐val) < 0.05. The results were visualized using volcano plots, and principal component analysis (PCA) was performed to assess overall expression variation and clustering patterns across samples.

### 2.3 Gene ontology (GO) and Kyoto encyclopedia of genes and genomes (KEGG) pathway enrichment analyses

A total of 433 DEGs were identified, including 304 upregulated and 129 downregulated genes. Functional annotation and enrichment analyses were performed using the clusterProfiler package, covering GO categories—biological process (biological process (BP)), cellular component, and molecular function (MF)—as well as KEGG pathways. A significance threshold of adjusted *p*‐value < 0.05 was applied for all enrichment results.

### 2.4 Cell culture

HL‐1 atrial cardiomyocytes were cultured in supplemented Claycomb medium (Sigma‐Aldrich) following previously established protocols. Prior to seeding, sterilized glass coverslips were coated with gelatin/fibronectin to enhance cell adherence. Cells were allowed to grow for approximately 96 h, typically reaching over 90% confluence. The cultures were then divided into two groups: a control group exhibiting spontaneous contraction and a stimulation group subjected to rapid electrical pacing at 300 beats per minute (bpm) for 24 h, a rate approximately 1.5–2 times the spontaneous frequency. Successful cellular response to pacing was confirmed via fluorescence microscopy by monitoring intracellular Ca^2+^ release, with cells preloaded with the calcium‐sensitive dye XRhod‐1 (5 μM, Invitrogen). Detailed experimental procedures are provided online as described in the Methods section.

### 2.5 Action potential (AP) recording

To assess the impact of rapid electrical stimulation on the AP waveform of HL‐1 cells, whole‐cell patch clamp recordings were done using the current clamp bridge mode. Cells grown on coverslips were exposed to electrical pacing (300 bpm for 24 h), while control cells maintained spontaneous activity. Recordings were done at a pacing frequency of 1 Hz and a constant temperature of 25°C, using an EPC‐10 amplifier and Patchmaster software (HEKA, Germany). The extracellular solution (Tyrode's solution) contained 135 mM NaCl, 5.4 mM KCl, 1.8 mM CaCl_2_, 1.0 mM MgCl_2_, 10 mM HEPES, and 10 mM glucose; their pH values were adjusted to 7.4. The intracellular solution comprised 110 mM K‐aspartate, 20 mM KCl, 5 mM MgATP, 10 mM EGTA, and 10 mM HEPES, and their pH values were adjusted to 7.2. AP waveforms were recorded and analyzed to assess changes in their duration and morphology.

### 2.6 Analysis of AP duration at 90% repolarization (APD_90_)

To quantify changes in repolarization dynamics, the APD_90_ was measured. Raw AP recordings were analyzed using Clampfit software (Molecular Devices, USA). APD_90_ was defined as the time from the peak of the AP to the point where the membrane potential repolarized to 90% of its peak amplitude. Recordings were performed under stimulation frequencies of 0.5, 1, and 2 Hz in both control and paced groups, with each condition replicated three times. Results were expressed in milliseconds and subjected to subsequent statistical analysis.

### 2.7 Recording of L‐type calcium channel currents

To assess L‐type calcium currents (I_Ca,L_), whole‐cell patch clamp recordings were done on HL‐1 cardiomyocytes under voltage clamp conditions. Cells were divided into a control group and a paced group (stimulated at 300 bpm for 24 h). Membrane potentials were stepped from −90 mV or −40 mV to +60 mV in 10 mV increments, with each step lasting 200 ms. The external recording solution was a calcium‐permeable Tyrode's solution containing 135 mM NaCl, 5.4 mM CsCl, 1.8 mM CaCl_2_, 1.0 mM MgCl_2_, 10 mM HEPES, and 10 mM glucose, adjusted to pH 7.4. The internal pipette solution consisted of 120 mM CsCl, 5 mM MgATP, 10 mM EGTA, and 10 mM HEPES, adjusted to pH 7.2. Recordings were done using an EPC‐10 amplifier (HEKA, Germany), and data were acquired using Patchmaster software. Peak Ca^2+^ current amplitudes at each voltage step were used for subsequent analyses.

### 2.8 Concentration–response relationship of nimodipine

To evaluate the sensitivity of L‐type calcium channels to pharmacological blockade, HL‐1 cells from both control and paced groups were exposed to increasing concentrations of nimodipine (0.01, 0.1, 1, and 10 μM). Changes in I_Ca,L_ were recorded, and the percentage inhibition was calculated for each concentration. Nimodipine (HY‐10350) was obtained from MedChemExpress (MCE, USA), prepared in 0.1% dimethyl sulfoxide (DMSO) as a stock solution, and then diluted to the desired concentrations for experimental use. Concentration–inhibition curves were fitted using nonlinear regression to calculate the half‐maximal inhibitory concentration, and data analysis was conducted with GraphPad Prism 10.

### 2.9 Current–voltage (I–V) relationship analysis

To investigate the effect of rapid pacing on the voltage‐dependent activation of L‐type calcium channels, I–V curves were generated using whole‐cell patch clamp recordings. HL‐1 cells were preloaded with the calcium‐sensitive dye X‐Rhod‐1 (X14210, Invitrogen, USA) to confirm responsiveness to electrical stimulation. Recordings were performed at room temperature using an EPC‐10 amplifier (HEKA) and Patchmaster software. Voltage steps ranged from −60 mV to +60 mV (10 mV increments), with holding potentials set at either −90 mV or −40 mV. The external Tyrode's solution contained 135 mM NaCl, 5.4 mM KCl, 1.8 mM CaCl_2_, 1.0 mM MgCl_2_, 10 mM HEPES, and 10 mM glucose (pH 7.4), while the internal solution comprised 120 mM CsCl, 5 mM MgATP, 10 mM EGTA, and 10 mM HEPES (pH 7.2). Nimodipine (MCE, HY‐10350) was used to assess channel sensitivity during recordings. Ca^2+^ current amplitudes (I_Ca,L_) were normalized to membrane capacitance, and I–V curves were plotted to characterize the voltage‐dependent activation properties of the channel.

### 2.10 2′,7′‐dichlorodihydrofluorescein diacetate (DCFH‐DA) fluorescence staining

HL‐1 atrial cardiomyocytes were seeded onto glass coverslips in 6‐well plates at a density of 5 × 10^5^ cells per well. Following experimental treatments, cells were rinsed twice with phosphate‐buffered saline (PBS) and incubated with 10 μM DCFH‐DA (S0033S, Beyotime) at 37°C for 30 min. After incubation, the cells were washed three additional times with PBS to remove excess probe. Intracellular ROS levels were visualized using a fluorescence microscope (Leica DMi8, Leica Microsystems) under the fluorescein isothiocyanate (FITC) channel, and representative images were captured. Quantitative analysis was performed by measuring the mean fluorescence intensity per cell within randomly selected fields of view for each experiment, with at least three independent fields analyzed per condition.

### 2.11 WB analysis

Total protein from cultured cells was extracted using radioimmunoprecipitation assay (RIPA) lysis buffer (P0013B, Beyotime). For tissue samples, left atrial tissue from rats was rapidly snap frozen in liquid nitrogen and homogenized in chilled tubes containing RIPA buffer supplemented with protease and phosphatase inhibitor cocktail (P1045, Beyotime). Homogenates were incubated on ice for 30 min with intermittent vortexing every 10 min, followed by centrifugation at 12,000 × g for 15 min. Protein concentrations in the supernatant were determined using the bicinchoninic acid assay kit (23227, Thermo Fisher Scientific), and samples were denatured by boiling. Equal amounts of protein (20 μg per lane) were resolved on 10% sodium dodecyl sulfate–polyacrylamide gel electrophoresis (SDS‐PAGE) gels and transferred onto polyvinylidene fluoride (PVDF) membranes (IPVH00010, Millipore). Membranes were incubated overnight at 4°C with the following primary antibodies (1:1000): anti‐p‐ERK1/2 (4370, CST), anti‐p‐p38 (4511, CST), anti‐p‐JNK (4668, CST), anti‐NOX2 (19013‐1‐AP, Proteintech), anti‐NOX4 (14347‐1‐AP, Proteintech), anti‐GAPDH (60004‐1‐Ig, Proteintech), anti‐P2X7 (13809, Cell Signaling Technology), anti‐NOX2 (MA5‐35348, Thermo Fisher Scientific), anti‐ERK1/2 (4695, Cell Signaling Technology), anti‐p38 (9212, Cell Signaling Technology), and anti‐JNK (9252, Cell Signaling Technology). The following day, horseradish peroxidase (HRP)‐conjugated secondary antibodies (SA00001‐2, Proteintech, China) were applied for 1 h at room temperature. Signals were visualized using enhanced chemiluminescence (ECL) reagents (P0018S, Beyotime), and images were acquired using a chemiluminescence imaging system (Tanon 5200, Tanon).

### 2.12 ELISA

To quantify inflammatory cytokines IL‐6, IL‐1β, TNF‐α, and IL‐18, ELISA was performed. Culture supernatants were collected from each group. For tissue analysis, 50–100 mg of left atrial tissue was placed in precooled EP tubes, homogenized in 1 mL of cold PBS using an electric homogenizer and centrifuged at 12,000 × g for 15 min. Cytokine levels were measured using mouse‐specific ELISA kits for IL‐6 (EK0411, Boster Biological Technology), IL‐1β (EK0393, Boster Biological Technology), mouse TNF‐α ELISA kit (PT512, Beyotime), and mouse IL‐18 ELISA kit (PI553, Beyotime), following the manufacturer's protocols. This included standard curve generation, sample loading, incubation, washing, and color development. Absorbance was measured at 450 nm using a microplate reader (SpectraMax iD3, Molecular Devices), and cytokine concentrations were calculated accordingly.

### 2.13 Transfection of P2X7 overexpression plasmid

HL‐1 cells were seeded in 6‐well plates at a density of 6 × 10^5^ cells per well. Once adherent, cells were transfected with either the P2X7 overexpression plasmid (pcDNA3.1‐P2X7, #60107, Addgene) or the empty vector control (pcDNA3.1) using Lipofectamine™ 3000 reagent (L3000015, Invitrogen), following the manufacturer's protocol. After 24 h, the medium was replaced with a fresh culture medium, and cells were incubated for an additional 24 h (total transfection time: 48 h).

### 2.14 Treatment with ROS scavengers

Cells were treated with N‐acetylcysteine (NAC) (A9165, Sigma‐Aldrich) at a final concentration of 5 mM or with apocynin (HY‐N0088, MedChemExpress) at a final concentration of 10 μM. Both agents were administered 1 h prior to stimulation, and the treatments were maintained for 48 h.

### 2.15 MitoSOX red staining for mitochondrial ROS detection

Cells were incubated with 5 μM MitoSOX™ Red (M36008, Invitrogen) in the dark at 37°C for 30 min. Following staining, nuclei were counterstained with Hoechst 33342 (C1027, Beyotime) for 5 min. Cells were subsequently washed with PBS and visualized under a fluorescence microscope to assess mitochondrial ROS (red fluorescence) and nuclear morphology (blue fluorescence). For quantitative analysis, 5–8 randomly selected fields per sample were analyzed in each group. The mean fluorescence intensity was calculated by measuring ≥50 cells per field, summing the fluorescence intensity across all analyzed cells and dividing it by the number of fields. At least, three independent fields of view were analyzed for each experimental condition, as previously described.^44^

### 2.16 Animal model establishment and grouping

Specific pathogen‐free (SPF) male Sprague Dawley (SD) rats, aged 5–6 weeks and weighing 160–200 g, were obtained from Beijing Vital River Laboratory Animal Technology Co., Ltd. The animals were housed under SPF conditions in a controlled environment with a temperature of 20–25°C and relative humidity of 40%–70%, with ad libitum access to food and water. All experimental procedures complied with institutional guidelines for animal ethics and welfare.

Rats were randomly assigned to three groups: control, model, and P2X7 inhibition (A‐438079) groups. The control group received daily tail vein injections of 0.1 mL/100 g normal saline for seven consecutive days. The model group received tail vein injections of an acetylcholine‐calcium chloride (Ach‐CaCl_2_) mixture (60 mg/L acetylcholine and 10 g/L calcium chloride, freshly prepared) at a dose of 0.1 mL/100 g/day for seven days to induce paroxysmal AF (PAF). In the A‐438079 group, in addition to the Ach‐CaCl_2_ induction protocol, rats were administered the selective P2X7 receptor antagonist A‐438079 (30 mg/kg/day; HY‐112205, MedChemExpress) via intraperitoneal injection for seven days. On day eight, all animals were anesthetized, and their left atrial tissues were collected for subsequent analyses.^45^

### 2.17 PAF model evaluation

Following intraperitoneal anesthesia with 20% urethane (0.5 mL/100 g), rats were connected to a limb lead electrocardiogram (ECG) system. PAF was induced via tail vein injection of the Ach‐CaCl_2_ mixture. The onset of AF was defined by the disappearance of P waves and the appearance of f waves, while termination was marked by the reappearance of P waves and loss of f waves. These criteria were used to confirm successful PAF model establishment.^45^

### 2.18 ECG‐based assessment of AF susceptibility

Upon successful model induction, ECG recordings were obtained using LabChart software to capture the timing of Ach‐CaCl_2_ injection and the initiation and termination of AF episodes. The AF induction latency and duration were calculated for each group to evaluate AF susceptibility.^45^

### 2.19 Electrophysiological mapping of atrial conduction

Krebs–Henseleit (K‐H) solution was prepared and equilibrated with a gas mixture of 95% O_2_ and 5% CO_2_ for at least 30 min. The pH was adjusted to 7.4. The oxygenated solution was then transferred into the pre‐warmed reservoir of the perfusion apparatus and maintained at 37°C. For each experimental group, three rats were randomly selected. Following intravenous administration of 105 IU L^−1^ heparin (1 mL/100 g) for anticoagulation, the animals were anesthetized. A midline thoracotomy was performed to expose the heart, which was then excised along with the aortic arch and rinsed in cold K‐H solution. The aortic stump was gently dilated using forceps and cannulated for connection to the Langendorff perfusion system. Perfusion pressure was maintained at 80 mmHg. Electrophysiological mapping was conducted using the MappingLab system, with electrode placement standardized across all samples. Once stable atrial conduction was achieved, atrial activation signals were recorded, and conduction heterogeneity was quantified. Data were analyzed using EMapScope 5.0 software to compare intergroup differences.

### 2.20 Echocardiographic assessment of left atrial geometry

After hair removal and disinfection of the thoracoabdominal region, transthoracic echocardiography was performed using a high‐resolution imaging system for small animals. Two‐dimensional images were acquired to measure left atrial diameter and area, enabling evaluation of structural remodeling across groups.

### 2.21 Masson's trichrome staining for collagen fiber assessment

After standard dewaxing, atrial tissue sections underwent Masson's trichrome staining. The protocol involved staining with Weigert's iron hematoxylin for 5 min, followed by differentiation in acid ethanol for 5–15 s and rinsing in distilled water. Sections were then stained with Biebrich scarlet‐acid fuchsin for 5–10 min, washed with weak acid solution for 1 min, immersed in phosphomolybdic acid for 1–2 min, washed again in weak acid, stained with aniline blue for 1–2 min, and finally subjected to a final weak acid wash. Tissues were dehydrated, cleared in xylene, and mounted with neutral resin. Collagen fiber distribution in atrial myocardium was evaluated under a light microscope in randomly selected fields.

### 2.22 Hematoxylin and eosin (H&E) staining

To evaluate general histopathological changes and inflammatory cell infiltration, atrial tissue sections were subjected to H&E staining. Following deparaffinization and rehydration, sections were stained with hematoxylin solution for 5 min to label nuclei, followed by differentiation in 1% acid alcohol and bluing in ammonia water. Sections were then counterstained with eosin for 2–3 min to label the cytoplasm and the extracellular matrix. After dehydration through graded alcohols and clearing in xylene, slides were mounted with neutral resin. Histological alterations, including cardiomyocyte arrangement and inflammatory infiltration, were examined under a light microscope. For the quantification of inflammatory infiltration, a semi‐quantitative scoring analysis was performed based on previously described methods.^46^ Five randomly selected fields per section were analyzed at a high magnification. The severity of inflammatory cell infiltration was scored or the number of infiltrating inflammatory cells was counted to determine the average inflammatory density for each group.

### 2.23 Immunohistochemical analysis of tyrosine hydroxylase (TH) expression in atrial tissue

Paraffin‐embedded tissue sections from each group underwent standard deparaffinization and rehydration, followed by antigen retrieval and incubation with 3% hydrogen peroxide to block endogenous peroxidase activity. After serum blocking, primary and secondary antibodies were sequentially applied, with PBS used as the negative control. Color development was achieved using DAB, and sections were counterstained and mounted. The presence of brown‐colored granules under the microscope was considered indicative of positive TH expression. Immunoreactive sympathetic nerve fibers were automatically identified and quantified using the Image‐Pro Plus 6.0 morphometric analysis system. For each section, six randomly selected fields were analyzed. The average nerve density was expressed as the ratio of the nerve area to the total analyzed area (μm^2^/mm^2^). The degree of heterogeneity in nerve distribution was quantified by calculating the mean difference between the fields with the highest and lowest nerve densities.

### 2.24 Statistical analysis

Statistical analyses were performed using GraphPad Prism 10. All datasets were assessed for normality using the Shapiro–Wilk test. Differences among multiple treatment groups or time points were analyzed using one‐way analysis of variance followed by Sidak's multiple comparison test. Comparisons between two groups were conducted using Student's *t*‐test. Sample size and statistical power for cell‐based experiments were estimated using G*Power version 3.1, with the following parameters: *α* = 0.05, effect size (d) = 1.2, and *n* = 3. A *p*‐value of less than 0.05 was considered statistically significant.

## 3 RESULTS

### 3.1 P2X7 as a potential initiator of oxidative stress and inflammatory responses in AF

**FIGURE 1:**
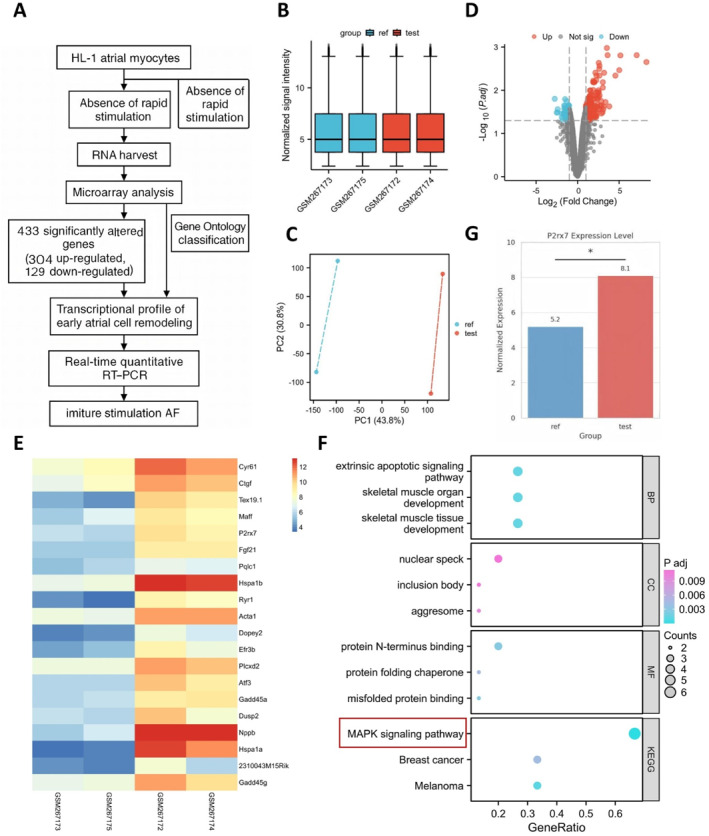
Transcriptomic profiling of HL‐1 atrial cardiomyocytes under rapid electrical stimulation. (A) Experimental workflow: HL‐1 atrial cardiomyocytes were subjected to either rapid electrical stimulation or left untreated. RNA was extracted for microarray analysis and identification of DEGs. (B) Distribution of normalized signal intensities across RNA‐seq samples. (C) Principal component analysis reveals clear separation between ref and test groups along PC1 and PC2 dimensions, indicating distinct transcriptomic profiles. (D) Volcano plot displaying the distribution of DEGs—red denotes upregulated genes and blue denotes downregulated genes. (E) Heat map displaying the expression patterns of the top 20 upregulated genes. (F) Gene ontology and and Kyoto Encyclopedia of Genes and Genomes enrichment analysis bubble plots, showing that differentially expressed genes are significantly enriched in pathways such as mitogen‐activated protein kinase signaling, apoptotic signaling, and protein folding chaperone‐related pathways. (G) Bar graph showing a significant upregulation of P2rx7 expression in the test group. “Ref” refers to spontaneously beating HL‐1 cardiomyocytes (*n* = 2), and “test” refers to stimulated HL‐1 cardiomyocytes (*n* = 2). To minimize potential technical bias, batch effect correction was applied to the normalized expression matrix during data preprocessing. *p < 0.05.

To investigate early transcriptional regulatory changes associated with AF induced by rapid electrical stimulation, we analyzed the GSE10598 dataset from the GEO database. This dataset includes transcriptomic profiling of HL‐1 atrial cardiomyocytes subjected to rapid pacing, with microarray data collected from both paced and spontaneously beating control cells (Figure 1A). A total of four samples were analyzed: GSM267173 and GSM267175 as controls and GSM267172 and GSM267174 as the pacing group. After normalization, gene expression profiles demonstrated high consistency (Figure 1B). PCA revealed clear transcriptomic separation between the two groups, indicating significant transcriptional reprogramming in response to stimulation (Figure 1C).

Using the Limma package for differential expression analysis with thresholds set at |log_2_FC| > 1 and adjusted *p*‐value < 0.05, we identified 433 DEGs, including 304 upregulated and 129 downregulated genes (Figure 1D). A heat map illustrates the expression profiles of the top 20 upregulated genes (Figure 1E).

Functional enrichment analysis of DEGs (Figure 1F) revealed significant enrichment of the MAPK signaling pathway, along with pathways related to cellular stress, apoptosis, and protein homeostasis, such as the extrinsic apoptotic signaling pathway, protein folding chaperones, and inclusion body formation. GO BP analysis also indicated transcriptional activation of pathways associated with cell death, spindle dysfunction, and microtubule disruption.

Calcium signaling plays a pivotal role in cardiomyocytes, particularly in excitation–contraction coupling and signal transduction.^47^ Gene annotation revealed that P2rx7 was significantly upregulated in the paced group (log_2_FC = 2.87 and adj. p‐val = 0.022) (Figure 1G), suggesting that P2X7 may act upstream of ROS accumulation and MAPK pathway activation.

### 3.2 Rapid electrical stimulation upregulates P2X7 and activates the ROS‐MAPK signaling pathway

**FIGURE 2:**
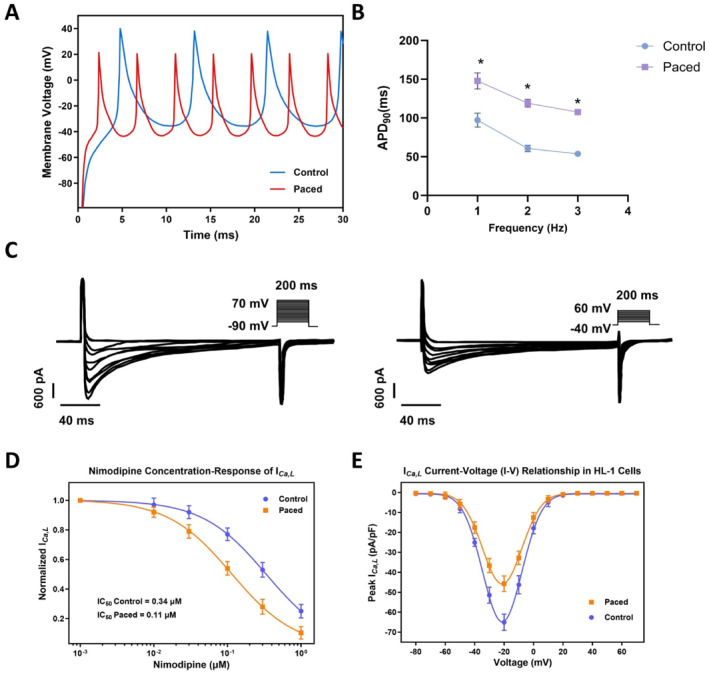
Shortening of action potential duration in HL‐1 cells following rapid electrical stimulation. (A) Representative action potential traces recorded from HL‐1 cells under control conditions and after 2 days of rapid pacing at 300 bpm; the recording frequency was 1 Hz. (B) Summary of APD_90_ measurements at different stimulation frequencies, comparing control and paced groups. (C) Representative I_Ca, L_ traces recorded via patch clamp, with voltage steps initiated from −90 mV (left) and −40 mV (right). (D) Dose–response curves of L‐type calcium current inhibition by varying concentrations of nimodipine. (E) Current–voltage (I–V) relationships under different holding potentials (−90 mV and −40 mV), illustrating voltage dependence. All experiments were repeated in triplicate (*n* = 3 biological replicates). *p < 0.05.

To investigate the electrophysiological effects of rapid electrical stimulation on atrial cardiomyocytes, APs in HL‐1 cells were recorded using the patch clamp technique in current clamp bridge mode. Under 1 Hz stimulation, HL‐1 cells subjected to rapid pacing (300 bpm for 48 h) exhibited significantly narrower AP waveforms compared to controls, indicating accelerated repolarization (Figure 2A).

Quantitative analysis revealed that the AP duration at 90% repolarization (APD_90_) was significantly shortened across various stimulation frequencies in the paced group relative to controls, with statistically significant differences (Figure 2B).

To elucidate the underlying mechanisms of APD shortening, L‐type calcium currents (I_Ca, L_) were assessed. Patch clamp recordings under stepwise depolarizations from −90 mV or −40 mV demonstrated a marked reduction in peak I_Ca, L_ in the paced group (Figure 2C).

Dose–response analysis following nimodipine administration showed enhanced current inhibition in paced cells, suggesting increased sensitivity to calcium channel blockade (Figure 2D). Additionally, current–voltage (I–V) relationship curves revealed diminished voltage‐dependent activation and a rightward shift in the activation threshold in the paced group (Figure 2E).

Collectively, these findings indicate that rapid pacing significantly shortens APD in HL‐1 cells, potentially due to impaired L‐type calcium channel function, reduced current density, and altered voltage sensitivity.

**FIGURE 3:**
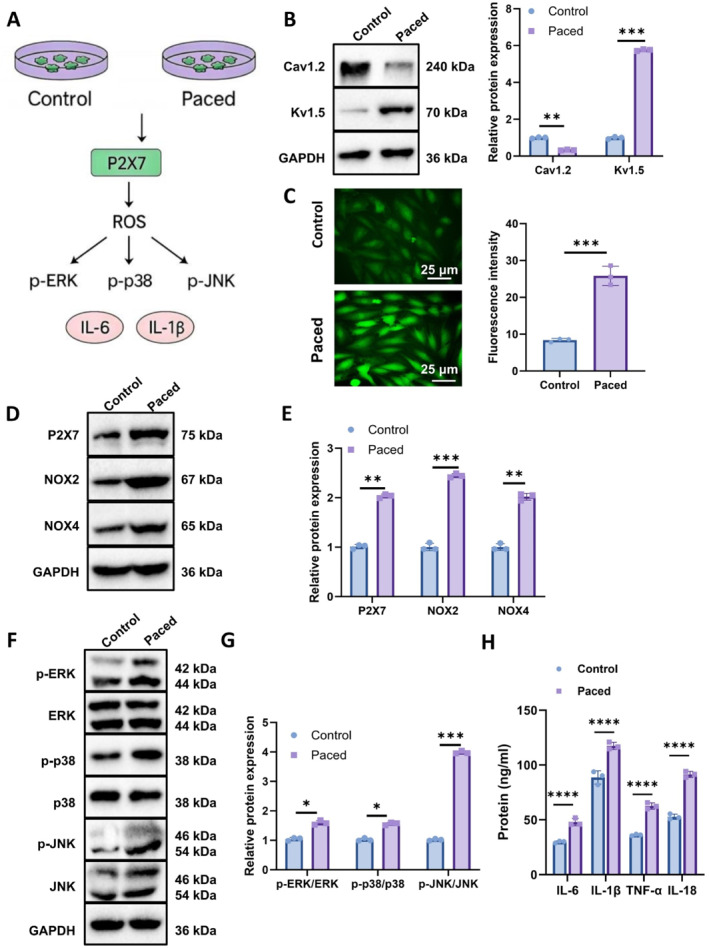
Effects of rapid electrical stimulation on ion channel expression, oxidative stress, and mitogen‐activated protein kinase (MAPK) pathway activation in HL‐1 cells. (A) Experimental grouping schematic. (B) WB analysis of Cav1.2 and Kv1.5 protein expression in control (control) and rapid stimulation (paced) groups. (C) Fluorescence microscopy images and quantitative analysis of reactive oxygen species accumulation using the DCFH‐DA fluorescent probe (scale bar: 50 μm). (D–E) WB analysis of ROS‐related proteins NOX2 and NOX4. (F) WB analysis of MAPK pathway proteins including phosphorylated and total ERK, p38, and JNK. (G) Quantification of relative expression levels of phosphorylated MAPK proteins (p‐ERK, p‐p38, and p‐JNK). (H) ELISA results showing concentrations of inflammatory cytokines IL‐6, IL‐1β, TNF‐α, and IL‐18 in cell culture supernatants. All experiments were performed in triplicate (*n* = 3 biological replicates). *p < 0.05, **p < 0.01, and ***p < 0.001.

To further characterize the phenotypic changes induced by rapid pacing, WB analysis was performed (Figure 3A). The paced group exhibited downregulation of Cav1.2 and upregulation of Kv1.5 (Figure 3B), consistent with shortened APD and accelerated repolarization.

Oxidative stress levels were then assessed using DCFH‐DA fluorescence. The paced cells showed significantly elevated ROS levels compared to controls (Figure 3C), indicating that rapid stimulation induces a pronounced oxidative stress response.

To determine whether ROS accumulation was mediated by P2X7, we assessed protein expression levels in the ROS‐MAPK axis. WB results revealed increased expression of ROS‐generating enzymes NOX2 and NOX4 in paced cells, implicating P2X7 activation in ROS upregulation (Figure 3D,E).

Further analysis showed significant increases in phosphorylated MAPK pathway proteins—p‐ERK, p‐p38, and p‐JNK—without changes in total protein levels, indicating pathway activation via enhanced phosphorylation (Figure 3F, and 3G). These results, together with elevated ROS levels, suggest that ROS may act as a key mediator of MAPK pathway activation.

To exclude nonspecific effects, ELISA was performed to assess the expression of inflammatory cytokines IL‐6 and IL‐1β. Both cytokines were significantly upregulated in the paced group (Figure 3H), reinforcing the activation of the P2X7‐ROS‐MAPK‐inflammation cascade.

### 3.3 The P2X7‐specific antagonist A‐438079 attenuates oxidative stress and MAPK pathway activation

**FIGURE 4:**
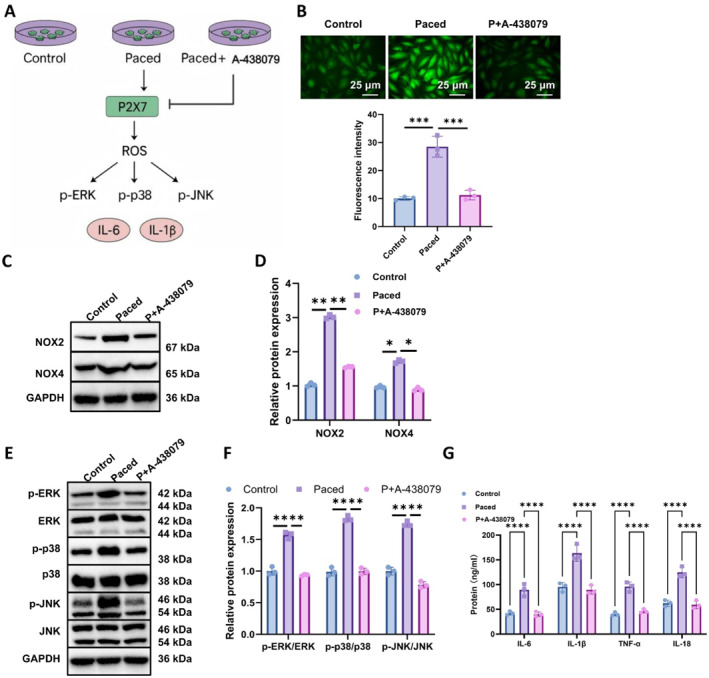
Inhibition of P2X7 confirms its central role in the ROS‐MAPK inflammation pathway. (A) Schematic overview of the experimental design, (B) representative fluorescence images and quantitative analysis of intracellular reactive oxygen species levels in HL‐1 cells from control, paced, and paced + A‐438079 groups using DCFH‐DA staining (scale bar: 50 μm), (C–F) WB analysis and corresponding densitometry of ROS‐generating enzymes and mitogen‐activated protein kinase pathway proteins, and (G) ELISA quantification of IL‐6, IL‐1β, TNF‐α, and IL‐18 levels in cell culture supernatants. All cell experiments were performed in triplicate (*n* = 3 biological replicates). *p < 0.05, **p < 0.01, ***p < 0.001, and ****p < 0.0001.

To elucidate the role of P2X7 in the ROS‐MAPK‐inflammatory signaling cascade, we established three experimental groups: a control group (spontaneous pacing), a paced group (rapid electrical stimulation), and a paced group treated with the P2X7‐specific antagonist A‐438079 (P + A‐438079). These groups were systematically assessed for intracellular ROS levels, MAPK phosphorylation status, and pro‐inflammatory cytokine expression (Figure 4A).

Intracellular ROS levels were measured using the DCFH‐DA fluorescent probe. HL‐1 cells in the paced group exhibited a marked increase in green fluorescence intensity compared to the control group, indicating substantial ROS accumulation following electrical stimulation. In contrast, the P + A‐438079 group displayed a significant reduction in fluorescence intensity relative to the paced group, suggesting that P2X7 inhibition effectively mitigates stimulation‐induced oxidative stress (Figure 4B).

WB analysis further revealed that phosphorylation levels of MAPK pathway components—p‐ERK, p‐p38, and p‐JNK—were significantly elevated in the paced group. Concurrently, the expression of ROS‐related enzymes NOX2 and NOX4 was also upregulated. Treatment with A‐438079 in the P + A‐438079 group markedly reduced the expression of these proteins, indicating that P2X7 activity contributes to both MAPK pathway activation and ROS generation (Figure 4C–F).

In parallel, ELISA assays demonstrated that levels of IL‐6, IL‐1β, TNF‐α, and IL‐18 were significantly increased in the paced group compared to the control, while treatment with A‐438079 effectively suppressed their expression. These findings implicate P2X7 activation as a key upstream driver of inflammatory cytokine release (Figure 4G).

In addition, to further demonstrate that the upregulation of NOX2/NOX4 expression is attributable to P2X7 activation, an NADPH oxidases (NOX) inhibitor (apocynin) was employed. Three experimental groups were established: a control group (spontaneous beating), a paced group (rapid electrical stimulation), and a paced + apocynin group (rapid electrical stimulation combined with apocynin treatment). Changes in ROS levels, MAPK phosphorylation, and inflammatory cytokine expression were systematically evaluated (Figure S1A).

First, intracellular ROS levels were assessed using the DCFH‐DA fluorescent probe. The results showed that consistent with the effects observed with A‐438079, ROS fluorescence intensity in the paced + apocynin group was markedly reduced compared with that in the paced group (Figure S1B).

Subsequently, WB analysis was performed to examine the activation status of the MAPK signaling pathway and the expression of ROS‐generating proteins. Compared with the control group, the paced group exhibited significantly increased phosphorylation levels of p‐ERK, p‐p38, and p‐JNK, along with elevated protein expression of NOX2 and NOX4. In contrast, the expression levels of these proteins were significantly decreased in the paced + apocynin group relative to the paced group (Figure S1C–F).

Furthermore, the secretion levels of inflammatory cytokines IL‐6, IL‐1β, TNF‐α, and IL‐18 were quantified by ELISA. The results demonstrated that the levels of these cytokines were significantly increased in the paced group compared with the control group, whereas treatment with apocynin markedly reduced the levels of IL‐6, IL‐1β, TNF‐α, and IL‐18 in the paced + apocynin group (Figure S1G).

### 3.4 MAPK pathway activation mediated by P2X7 is dependent on ROS accumulation

**FIGURE 5:**
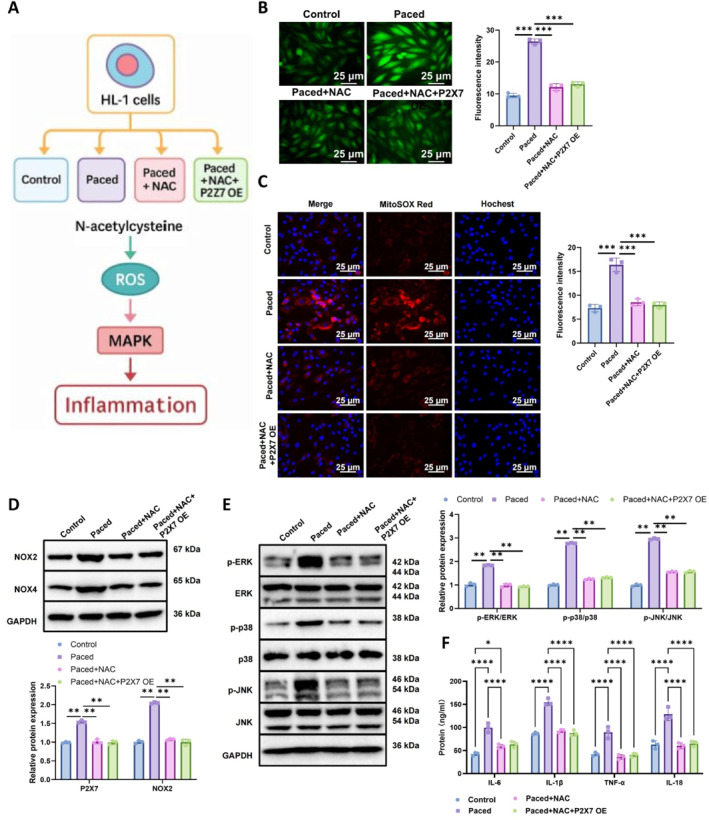
Reactive oxygen species (ROS) as a critical mediator of P2X7‐induced mitogen‐activated protein kinase (MAPK) activation and inflammatory cytokine expression. (A) Experimental workflow illustrating four groups, control, paced, paced + NAC, and paced + NAC + P2X7 overexpression (OE), to assess whether MAPK signaling and inflammatory responses depend on ROS accumulation, (B) DCFH‐DA staining of cytosolic ROS in HL‐1 cells across groups, with quantification of fluorescence intensity (scale bar: 50 μm), (C) MitoSOX Red staining to visualize mitochondrial ROS levels (scale bar: 50 μm), (D and E) WB detection of phosphorylated MAPK proteins (p‐ERK, p‐p38, and p‐JNK) and ROS‐associated enzymes (NOX2 and NOX4), with corresponding densitometry, and (F) ELISA measurement of IL‐6, IL‐1β, TNF‐α, and IL‐18 levels in supernatants. All cell experiments were conducted in triplicate (*n* = 3 biological replicates). *p < 0.05, **p < 0.01, ***p < 0.001, and ****p < 0.0001; ns, not significant.

To further elucidate the mediating role of ROS in P2X7‐regulated MAPK signaling, we performed experiments involving ROS scavenging with NAC in combination with P2X7 overexpression. This allowed us to assess whether MAPK activation by P2X7 is contingent upon intracellular ROS levels. Four experimental groups were established: control, paced, paced + NAC, and paced + NAC + P2X7 overexpression (OE) (Figure 5A).

DCFH‐DA staining (Figure 5B) revealed minimal fluorescence in the control group, consistent with basal ROS levels. In contrast, the paced group exhibited significantly intensified green fluorescence, indicating cytosolic ROS accumulation in response to rapid electrical stimulation. NAC treatment markedly reduced ROS fluorescence in the paced + NAC group, confirming its efficacy in ROS elimination. Notably, even with P2X7 overexpression in the aced + NAC + P2X7 OE group, fluorescence intensity remained comparable to that of the paced + NAC group, suggesting that P2X7 overexpression cannot induce ROS accumulation in the absence of upstream oxidative signaling.

MitoSOX staining (Figure 5C), used to assess mitochondrial ROS, corroborated these findings. Mitochondrial ROS levels were significantly elevated in the paced group, while NAC treatment reduced both cytosolic and mitochondrial ROS. Again, in the presence of NAC, P2X7 overexpression failed to restore elevated ROS levels, further supporting the notion that P2X7‐mediated ROS regulation requires intact ROS‐generating pathways.

WB analysis (Figure 5D and 5E) showed that phosphorylation of MAPK pathway components (p‐ERK, p‐p38, and p‐JNK) and the expression of ROS‐generating enzymes (NOX2 and NOX4) were markedly increased in the paced group. These effects were significantly attenuated by NAC treatment. Importantly, even with robust P2X7 overexpression, MAPK phosphorylation and NOX expression levels remained low under ROS‐depleted conditions, indicating that ROS accumulation is essential for P2X7‐driven MAPK activation.

ELISA results for pro‐inflammatory cytokines (Figure 5F) further confirmed this dependency: IL‐6, IL‐1β, TNF‐α, and IL‐18 secretion increased substantially in the paced group but were significantly reduced by NAC. Overexpression of P2X7 in the NAC‐treated background failed to elevate cytokine levels, remaining statistically indistinguishable from the paced + NAC group.

### 3.5 Effect of P2X7 receptor inhibition on atrial electrical and structural remodeling in a PAF animal model

**FIGURE 6:**
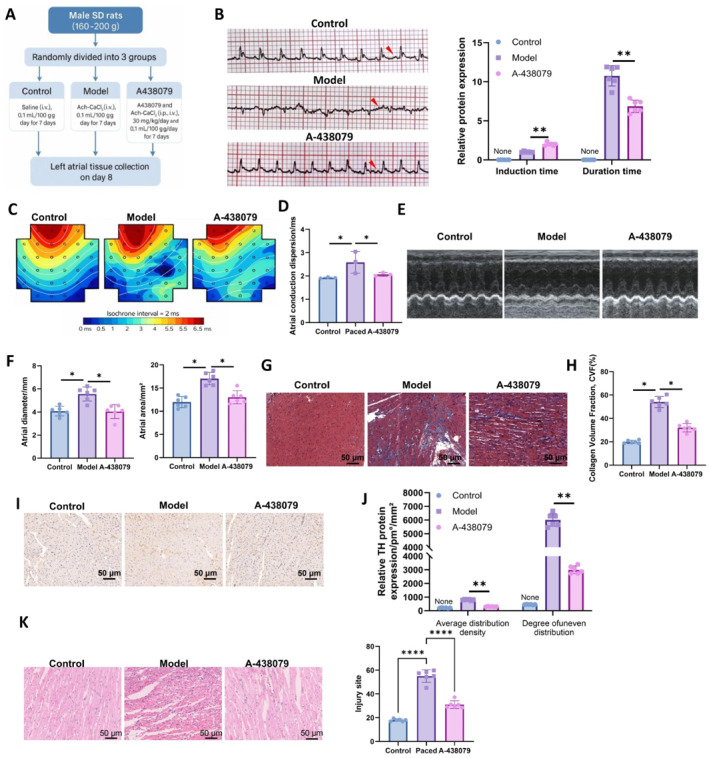
Inhibition of the P2X7 receptor improves atrial conduction and structural remodeling in PAF rats. (A) Schematic of experimental grouping and treatment protocol, (B) representative ECG recordings from each group, (C and D) atrial conduction dispersion assessed using the MappingLab system, (E and F) echocardiographic measurement of the left atrial systolic diameter and the area, (G and H) Masson's trichrome staining to evaluate atrial collagen deposition (scale bar: 50 μm), (I and J) immunohistochemical detection of tyrosine hydroxylase protein expression in atrial tissue (scale bar: 50 μm), and (K) representative hematoxylin and eosin (H&E) staining images showing pathological alterations in each group. *n* = 6 animals per group. *p < 0.05, **p < 0.01, and ****p < 0.0001; ns indicates no significant difference.

Animals were divided into different groups for pharmacological intervention, as illustrated in Figure 6A. As shown in Figure 6B, rats in the control group exhibited normal ECG patterns, with clearly discernible P waves and regular RR intervals. In contrast, rats in the model group displayed disappearance of P waves, which were replaced by irregular f waves, accompanied by absolutely irregular RR intervals, confirming the successful induction of acute AF episodes. In the A‐438079 intervention group, partial restoration of P waves was observed on the ECG recordings, and the rhythm of the QRS complexes appeared relatively regular. Moreover, both the amplitude and the density of f waves were markedly reduced compared with those in the model group. These findings suggest that P2X7 receptor inhibition may attenuate the degree of atrial electrical remodeling and partially alleviate the occurrence of AF. Subsequently, ECG recordings were used to assess AF inducibility and duration in each group to evaluate AF susceptibility in PAF rats. As quantified in Figure 6B, A‐438079 treatment significantly prolonged the AF induction time and shortened the duration of AF episodes compared to the model group, indicating that P2X7 inhibition mitigates AF susceptibility in PAF rats.

Next, atrial conduction heterogeneity was evaluated using the MappingLab mapping system (*n* = 3 per group). Figure 6C and 6D reveal a marked increase in conduction dispersion in the model group compared to controls. Notably, treatment with A‐438079 substantially reduced this parameter relative to the model group, implying improved atrial conduction homogeneity upon P2X7 inhibition.

Echocardiographic measurements of the left atrial diameter and the area during ventricular systole were used to evaluate structural remodeling. As shown in Figure 6E–G, the model group displayed significant left atrial enlargement compared to controls. These structural changes were attenuated in the A‐438079 group, as evidenced by significantly reduced atrial diameter and area, indicating that P2X7 inhibition may effectively limit atrial structural remodeling in PAF.

To further investigate tissue‐level alterations, Masson's trichrome staining was performed to evaluate atrial fibrosis. Figure 6G and 6H show orderly myocardial fiber arrangement and minimal collagen deposition in the control group. In contrast, the model group exhibited disorganized cardiomyocyte architecture with prominent perivascular collagen accumulation. A‐438079 treatment partially restored myocardial alignment and markedly reduced collagen deposition, supporting the role of P2X7 inhibition in mitigating fibrotic remodeling.

Finally, to assess sympathetic nerve remodeling, TH expression was analyzed via immunohistochemistry. As shown in Figure 6I and 6J, the model group demonstrated increased TH density and heterogeneous distribution, with disordered, bundle‐like nerve projections—hallmarks of sympathetic remodeling. A‐438079 treatment led to a significant reduction in both TH density and distributional inhomogeneity. These findings suggest that P2X7 inhibition may also ameliorate sympathetic nerve remodeling in PAF. In addition, as shown by H&E staining, compared with the control group, atrial tissues from the model group exhibited markedly disorganized tissue architecture, accompanied by extensive fibrotic tissue proliferation, prominent inflammatory cell infiltration, and increased neovascularization. In contrast, treatment with A‐438079 substantially reversed these pathological alterations, resulting in improved atrial tissue structure and reduced inflammatory infiltration (Figure 6K).

### 3.6 In vivo validation of P2X7‐mediated MAPK activation and inflammatory response via ROS

**FIGURE 7:**
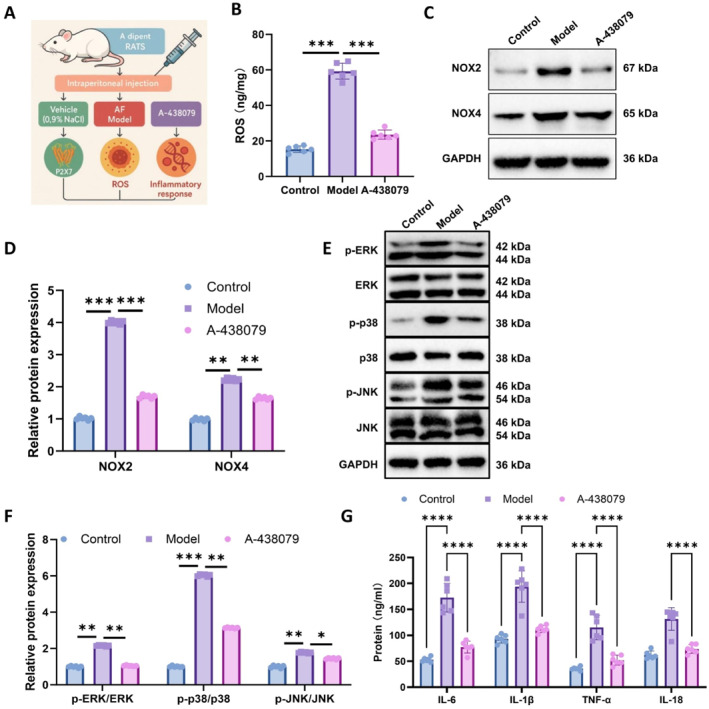
In vivo validation showing that P2X7 receptor activation promotes mitogen‐activated protein kinase signaling and inflammation via reactive oxygen species (ROS) in atrial tissue. (A) Experimental workflow diagram, (B) DCFH‐DA fluorescence staining of ROS levels in left atrial tissue (scale bar: 50 μm), (C–F) WB analysis of NOX2, NOX4, phosphorylated and total ERK, p38, and JNK proteins, and (G) ELISA quantification of IL‐6, IL‐1β, TNF‐α, and IL‐18 in left atrial tissue. Sample size per group: *n* = 6. *p < 0.05, **p < 0.01, ***p < 0.001, and ****p < 0.0001.

To further investigate whether P2X7 mediates MAPK pathway activation and inflammatory responses through ROS, we evaluated the expression of P2X7, ROS levels, MAPK pathway activation, and pro‐inflammatory cytokine expression in a rat model of PAF, as illustrated in Figure 7A.

Quantitative analysis of ROS in left atrial tissue was performed by an ROS detection assay (Figure 7B). Compared with the control group, the PAF model group exhibited a significant elevation in ROS levels. Notably, treatment with the selective P2X7 inhibitor A‐438079 markedly reduced ROS accumulation, indicating that P2X7 activation may facilitate ROS production in atrial tissue, while its inhibition effectively mitigates oxidative stress.

WB analysis (Figure 7C–G) revealed that expression levels of NOX2 and NOX4 were substantially increased in the model group relative to controls, accompanied by pronounced phosphorylation of key MAPK pathway components—p‐ERK, p‐p38, and p‐JNK. These elevations were significantly attenuated following A‐438079 treatment, suggesting that P2X7‐driven MAPK activation is dependent on ROS‐mediated upstream signaling.

Further supporting this, ELISA results (Figure 7G) demonstrated elevated concentrations of IL‐6, IL‐1β, TNF‐α, and IL‐18 in the model group, all of which were significantly reduced upon P2X7 inhibition. These findings corroborate the role of P2X7 in triggering inflammation through ROS signaling in vivo.

## 4 DISCUSSION

In the present study, we integrated transcriptomic analysis with in vivo and in vitro experiments to elucidate a novel mechanism by which P2X7 receptor activation contributes to the pathogenesis of AF. Our findings demonstrate that P2X7 acts as a critical upstream switch that promotes ROS accumulation via NOX2/NOX4 upregulation. This oxidative stress subsequently triggers the phosphorylation of MAPK signaling pathways and the release of pro‐inflammatory cytokines (IL‐6 and IL‐1β). Crucially, we provide evidence that this P2X7‐ROS‐MAPK axis is causally linked to atrial electrical remodeling, structural remodeling (fibrosis), and autonomic remodeling (sympathetic hyperinnervation). Consequently, inhibition of P2X7 significantly attenuates these remodeling processes and reduces AF susceptibility in a rat model. To our knowledge, this is the first study to systemically verify the P2X7‐ROS‐MAPK axis as a key driver of the atrial substrate for AF.

Biological functions of P2X7. As an ATP‐gated cation channel, P2X7 has garnered increasing attention for its involvement in cardiovascular pathology.^13^ Previous research has primarily focused on its pro‐inflammatory and pro‐fibrotic roles in MI, cardiac fibrosis, and hypertension models.^17^ For instance, P2X7 activation has been shown to exacerbate myocardial injury through NLRP3 inflammasome‐mediated cell death.^21^ Building on this foundation, our study extends the functional relevance of P2X7 to AF, providing the first mechanistic evidence of its pathogenic involvement in this arrhythmia. This expansion not only broadens the biological framework of P2X7 but also offers new mechanistic insight into the multifactorial etiology of AF.

The P2X7 receptor promotes ROS generation by enhancing the expression and activity of NOX2 and NOX4, and ROS acts as a key mediator driving MAPK activation and inflammatory cytokine release. Elucidation of the causal relationship between P2X7 and ROS and identification of P2X7 as an upstream signaling initiator of ROS production provide a solid theoretical basis for antioxidant‐based therapeutic interventions. The pivotal role of ROS in AF has been substantiated by numerous studies, with key sources including mitochondrial leakage, inflammatory responses, and activation of NOX such as NOX2 and NOX4.^25^, ^28^ While previous research has primarily characterized ROS as a downstream effector in inflammation and structural remodeling, the upstream regulatory mechanisms governing ROS production remain poorly defined.^48^ In this study, we demonstrate for the first time that the P2X7 receptor significantly enhances ROS generation by upregulating NOX2 and NOX4 expression and activity. This ROS accumulation, in turn, drives activation of the MAPK signaling cascade and induces pro‐inflammatory cytokine release. Notably, ROS scavenging with NAC abolished MAPK activation, even in the context of P2X7 overexpression, indicating a strict ROS dependency within this signaling axis. These findings not only establish a causal link between P2X7 and ROS but also position P2X7 as a critical upstream modulator of oxidative stress, thereby enriching our understanding of ROS regulation. Contrary to earlier studies that regarded ROS merely as a byproduct of disease progression,^49^, ^50^ our results delineate ROS as an integral mediator within the P2X7‐ROS‐MAPK axis, offering a mechanistic foundation for targeted antioxidant interventions.

Advances in MAPK signaling research show that the MAPK signaling pathway, a central regulator of cellular responses to stress, governs diverse biological processes including proliferation, apoptosis, differentiation, and inflammation.^51^ Prior studies have shown that p38 and JNK activation under oxidative stress can stimulate fibroblast activation and extracellular matrix deposition, contributing to atrial fibrosis.^38^, ^52^ Consistent with these findings, our pacing‐induced AF model revealed upregulation of phosphorylated ERK, p38, and JNK, all of which correlated with increased P2X7 activity and ROS levels, underscoring the MAPK pathway's key role in AF‐related electrical and structural remodeling. Additionally, we observed that MAPK activation led to elevated expression of inflammatory cytokines, highlighting its function as a conduit between oxidative stress and inflammatory amplification. Unlike studies focusing on isolated MAPK branches, our integrated analysis of all three subfamilies (ERK, p38, and JNK) provides a more comprehensive depiction of pathway activation. Moreover, pharmacological inhibition of P2X7 or ROS effectively attenuated MAPK activation, reinforcing the hierarchical relationship within this signaling cascade. These insights position the MAPK pathway as a convergence hub for multiple pathological signals and support its potential as a therapeutic target. Notably, functional heterogeneity exists among MAPK subfamilies. Under conditions of cardiac pressure overload, such as hypertension or rapid pacing, ERK1/2 is persistently activated and promotes cardiac hypertrophy by phosphorylating intracellular transcription factors, including c‐Myc and Elk‐1, thereby inducing the expression of hypertrophy‐associated genes such as ANP and BNP, which directly drive atrial and ventricular hypertrophy.^53^ Consistent with these reports, our study demonstrated that p‐ERK1/2 protein expression was significantly increased in rapidly paced HL‐1 atrial cardiomyocytes compared with control cells (*p* < 0.01; Figure 3F and 3G). In addition, ERK activation may indirectly contribute to myocardial interstitial fibrosis by stimulating fibroblast proliferation, further supporting the concept that ERK promotes atrial hypertrophy. In contrast, p38 and JNK are preferentially activated in response to oxidative stress (e.g., ROS accumulation) and pro‐inflammatory cytokine stimulation (e.g., IL‐1β and TNF‐α). In line with this, our results showed that p‐p38 and p‐JNK protein levels were significantly elevated following rapid electrical pacing (*p* < 0.05), accompanied by reduced cell viability, increased apoptosis, and enhanced secretion of the pro‐inflammatory cytokine IL‐6 (*p* < 0.01; Figure 3F–H). Mechanistically, p38 induces the expression of apoptosis‐related proteins, such as Bax and Caspase‐3, through phosphorylation of p53 and ATF2, whereas JNK promotes the release of inflammatory cytokines, including IL‐6 and IL‐8, via activation of the NF‐κB signaling pathway. Together, these pathways cooperatively mediate cardiac inflammation and cell death, directly supporting the established mechanism whereby p38/JNK signaling drives apoptosis and inflammation.^54^ Furthermore, mitochondrial ROS and MAPK signaling form a positive feedback loop, in which NLRP3 inflammasome activation and Ca^2+^ dysregulation may also participate, collectively amplifying oxidative stress and inflammatory responses and thereby accelerating AF‐related electrical and structural remodeling.^53^

Roles of IL‐6 and IL‐1β. Pro‐inflammatory cytokines IL‐6 and IL‐1β are consistently elevated in both peripheral blood and atrial tissue of AF patients, and their levels have been positively associated with disease duration and recurrence risk.^55^, ^56^ While past studies have primarily regarded these cytokines as biomarkers of AF, their upstream regulation has been insufficiently addressed.^57^, ^58^ Our findings reveal that P2X7 activation robustly increases IL‐6 and IL‐1β expression, whereas inhibition of either P2X7 or ROS markedly reduces their secretion. This suggests that these cytokines function not only as downstream markers but also as actively regulated effectors within the P2X7‐ROS‐MAPK network. Furthermore, the temporal co‐elevation of MAPK phosphorylation and cytokine release implies a direct regulatory linkage. In contrast to previous work that emphasized cytokine expression as a response to exogenous stimuli,^59^ our study positions IL‐6 and IL‐1β as components of an endogenous inflammatory feedback loop. This recontextualization offers new mechanistic insights and supports the rationale for targeting this axis in inflammation‐driven AF pathogenesis.

Mechanisms of AF show that atrial remodeling serves as a fundamental substrate for the persistence and recurrence of AF, encompassing electrical, structural, and autonomic components.^7^, ^60^ In this study, upregulation of the P2X7 receptor was found to be closely associated with a shortened APD and diminished L‐type calcium current, indicating its direct impact on electrophysiological stability. In vivo experiments further revealed that P2X7 activation promoted atrial enlargement, increased interstitial collagen deposition, and elevated NOX2/NOX4 expression, underscoring its pro‐fibrotic role in structural remodeling. Additionally, a marked rise in TH expression suggested enhanced sympathetic innervation, implicating P2X7 in the indirect modulation of autonomic remodeling via the ROS‐MAPK signaling axis. In addition, P2X7 signaling may exert ROS‐independent effects. Specifically, P2X7 signaling can directly influence sympathetic nerve remodeling through the NLRP3 inflammasome–IL‐1β axis, independently of ROS‐mediated mechanisms. In an MI model, P2X7 receptors are highly expressed in infiltrating macrophages, and their activation promotes IL‐1β release via activation of the NLRP3 inflammasome, which in turn induces macrophage secretion of nerve growth factor (NGF).^61^ As a key regulator of sympathetic nerve regeneration, NGF accelerates sympathetic nerve sprouting, thereby increasing the susceptibility to malignant arrhythmias.^62^ Unlike previous studies that tend to isolate individual remodeling pathways,^63^ our findings integrate electrical, structural, and neural alterations into a cohesive regulatory framework initiated by P2X7 signaling, thereby reflecting the complex, multidimensional nature of AF pathogenesis. This system‐level perspective facilitates the synthesis of disparate findings and supports a conceptual shift from localized lesions to a network‐based understanding of AF mechanisms.

The most notable innovation of the present study lies in the first systematic validation of the P2X7‐ROS‐MAPK axis as a pathogenic signaling cascade in AF. While prior literature often centers on isolated molecular events or singular pathways,^64^ our work elucidates a hierarchical signaling circuit beginning with the P2X7 receptor and extending through ROS production, MAPK activation, and subsequent inflammatory mediator release. Methodologically, this research integrates multiple platforms—including public transcriptomic databases, in vitro and in vivo models, electrophysiological profiling, fluorescence imaging, and protein quantification—to enhance the robustness and translational relevance of the conclusions. Moreover, we demonstrate the feasibility of pharmacological modulation at various points along this axis, highlighting the therapeutic viability of targeting P2X7. Compared with the fragmented characterizations of P2X7 in existing studies,^13^ our work provides a comprehensive mechanistic framework that substantially expands its recognized role in arrhythmogenic processes and lays a foundation for future target‐based interventions. Notably, the P2X7‐ROS‐MAPK mechanism may exhibit heterogeneity between chronic AF and rapid pacing‐induced AF models (such as the rapid electrical stimulation model used in the present study), which is closely related to the distinct pathophysiological characteristics of these two models. The rapid pacing model primarily mimics the early, acute electrical stress phase of AF, characterized by abrupt electrophysiological disturbances, burst oxidative stress, and rapid activation of inflammatory responses. In this context, P2X7 acts as a core initiating node, driving rapid and robust pathway activation, with downstream effects dominated by electrical remodeling. Consequently, this model is highly sensitive to P2X7 inhibition or ROS scavenging, and pathological phenotypes can be rapidly reversed following intervention.^65^, ^66^ In contrast, chronic AF is accompanied by largely irreversible pathological remodeling, including myocardial fibrosis, autonomic nerve remodeling, metabolic dysregulation, and other complex alterations. To date, the involvement of the P2X7–ROS–MAPK signaling pathway in chronic AF has not been clearly defined, as existing studies on chronic AF have predominantly focused on pharmacological treatment strategies, with relatively limited mechanistic exploration.^67^ By comparison, rapid pacing models are widely used to simulate the acute electrical stress state of early stage AF, featuring rapid‐onset electrophysiological instability, oxidative stress accumulation, and inflammatory activation.^32^ In the present study, the observed rapid upregulation of P2X7, acute ROS accumulation, and transient activation of MAPK signaling are highly consistent with this acute pathological process. Under these conditions, P2X7 functions as an “on–off switch” for stress signaling, rapidly activating the ROS–MAPK axis and directly triggering early electrical and structural remodeling. In future studies, we will further investigate the role of P2X7‐ROS‐MAPK signaling in chronic AF to determine whether this pathway also contributes to long‐term atrial remodeling.

Despite the systematic design and novelty of the present study, several limitations should be acknowledged. First, the lack of validation using atrial tissue samples from clinical patients represents an important limitation. In addition, some downstream effects of the identified signaling pathways were not explored in depth, and the long‐term efficacy and safety of pharmacological interventions remain to be evaluated. Second, functional validation of P2X7 in this study relied primarily on the small‐molecule inhibitor A‐438079. Although A‐438079 has been widely reported to exhibit high selectivity for P2X7^68^ and is considered an effective and selective P2X7 receptor antagonist capable of specifically blocking its ion channel activity and pore formation, its potential limitations must be objectively recognized. In our experiments, treatment with A‐438079 significantly attenuated stimulation‐induced ROS accumulation—for example, in paced HL‐1 cells, intracellular ROS fluorescence intensity was markedly increased compared with the control group, whereas ROS levels were substantially reduced in the paced + A‐438079 group (Figure 4B)—supporting the on‐target inhibitory effect of A‐438079 on P2X7. Nevertheless, we cannot fully exclude the possibility of low‐probability off‐target effects, particularly given that dose–response analyses were not systematically performed. In this study, A‐438079 was administered at 10 μM in vitro and 30 mg/kg/day in vivo, based on previously reported effective and safe dosage ranges in neurological studies.^69^ However, the absence of a comprehensive dose–response assessment represents a limitation. Therefore, to enhance mechanistic rigor, future studies should employ siRNA‐mediated P2X7 knockdown or CRISPR/Cas9‐mediated P2X7 gene knockout to establish P2X7‐deficient HL‐1 cell models and evaluate changes in ROS generation, MAPK activation, and inflammatory cytokine expression under rapid electrical pacing. In parallel, virus‐mediated, cardiomyocyte‐specific P2X7 knockdown (e.g., AAV9‐P2X7‐shRNA) could be implemented in animal models and directly compared with A‐438079 treatment to assess consistency in outcomes such as AF inducibility, atrial conduction heterogeneity, and collagen deposition. Moreover, although only male rats were used in the present study to avoid confounding effects related to sex hormones,^70^ we acknowledge the importance of considering sex as a biological variable in future investigations to achieve a more comprehensive understanding of P2X7‐mediated mechanisms in AF. Another important consideration is the potential impact of long‐term P2X7 inhibition. P2X7 plays physiological roles in immune regulation (e.g., macrophage activation and inflammasome modulation) and neural function (e.g., synaptic transmission and neuroinflammation).^13^, ^71^, ^72^ Prolonged blockade of P2X7 may therefore carry risks of impaired immune responses or neurological dysfunction. In the present study, the duration of A‐438079 intervention was shorter, and no overt adverse effects were observed. However, for clinical translation, the long‐term safety profile of P2X7 inhibition must be carefully evaluated to balance therapeutic benefits in AF against potential immune and neurological risks. Finally, the smaller sample size in some experiments (*n* = 3) represents an additional limitation. Future studies should incorporate larger sample sizes and integrate clinical cohort validation, CRISPR‐based genetic manipulation, and single‐cell transcriptomic approaches to further delineate the upstream and downstream networks of the P2X7‐ROS‐MAPK signaling axis and facilitate its translation into clinical applications.

## 5 CONCLUSION

This study systematically elucidates the pivotal regulatory role of the P2X7 receptor in the pathogenesis of AF. It is the first to demonstrate that P2X7 facilitates the accumulation of ROS, thereby driving the phosphorylation and activation of the MAPK signaling cascade. This activation subsequently promotes the expression of pro‐inflammatory cytokines such as IL‐6 and IL‐1β, contributing to both atrial electrical and structural remodeling as well as sympathetic nerve remodeling—key processes that increase the susceptibility to and persistence of AF. Transcriptomic analysis revealed significant upregulation of P2rx7 in a rapid electrical stimulation model, with enrichment in the MAPK pathway. In vitro experiments confirmed that P2X7 mediates ROS production and MAPK activation and that these effects are abrogated by ROS scavengers. Moreover, in vivo studies showed that the selective P2X7 antagonist A‐438079 markedly delayed AF onset, shortened its duration, reduced atrial conduction heterogeneity, and alleviated both structural remodeling and inflammatory responses. Collectively, these findings establish a novel pathological signaling axis—P2X7‐ROS‐MAPK‐inflammation‐AF susceptibility—offering new molecular insights into the mechanistic underpinnings of AF.

At the mechanistic level, this work expands the current understanding of atrial electrostructural remodeling by positioning P2X7 as a key molecular bridge linking electrophysiological alterations with oxidative stress. It also enriches the conceptual framework of inflammation‐driven AF. On the translational front, the study provides compelling evidence that A‐438079 can suppress the ROS‐MAPK signaling cascade and significantly ameliorate both electrical conduction defects and structural abnormalities in AF models, highlighting its promise as an early therapeutic target for molecular intervention.

While these conclusions are primarily derived from HL‐1 atrial myocyte models subjected to rapid pacing and from a transient AF animal model, further validation is required in chronic AF models and human atrial tissues to confirm the robustness and generalizability of the P2X7 axis. Additionally, P2X7 may engage other mechanisms, including calcium influx and NLRP3 inflammasome activation, suggesting broader roles in the immunoregulatory landscape of AF. Future research should focus on delineating the clinical expression profile of P2X7, mapping the upstream and downstream regulatory networks with higher resolution and evaluating its synergistic potential in combination with antioxidant or anti‐inflammatory therapies—ultimately advancing precise and targeted strategies for AF management.

## AUTHOR CONTRIBUTIONS

Lingnan Zhang and Yeran Zhu performed the experiments, collected and analyzed the data, and drafted the manuscript. Lingnan Zhang also contributed to the bioinformatics and electrophysiological analyses. Yeran Zhu participated in the in vivo animal studies and molecular assays. Xinshun Gu conceived and designed the study, supervised the research, interpreted the results, and critically revised the manuscript. All authors read and approved the final manuscript.

## CONFLICT OF INTEREST STATEMENT

The authors declare no conflicts of interest.

## ETHICS STATEMENT

All animal experiments were approved by the Animal Ethics Committee of The Second Hospital of Hebei Medical University (No. 20231026A). The clinical trial number is not applicable.

## Supporting information

Supporting Information S1

Figure S1

## Data Availability

All data generated or analyzed during this study are included in this article and/or its supplementary material files. Further inquiries can be directed to the corresponding author.
