# Supplementary material for: P2X7 receptors promote atrial remodeling and atrial fibrillation susceptibility via reactive oxygen species‐mediated mitogen‐activated protein kinase signaling activation
Source: J Cell Commun Signal. 2026 Apr 30;20(2):e70071. doi: 10.1002/ccs3.70071 (PMC13130150; doi:10.1002/ccs3.70071)
Supplement: Supplementary file 1 — Supporting Information S1 [file CCS3-20-e70071-s001.docx]

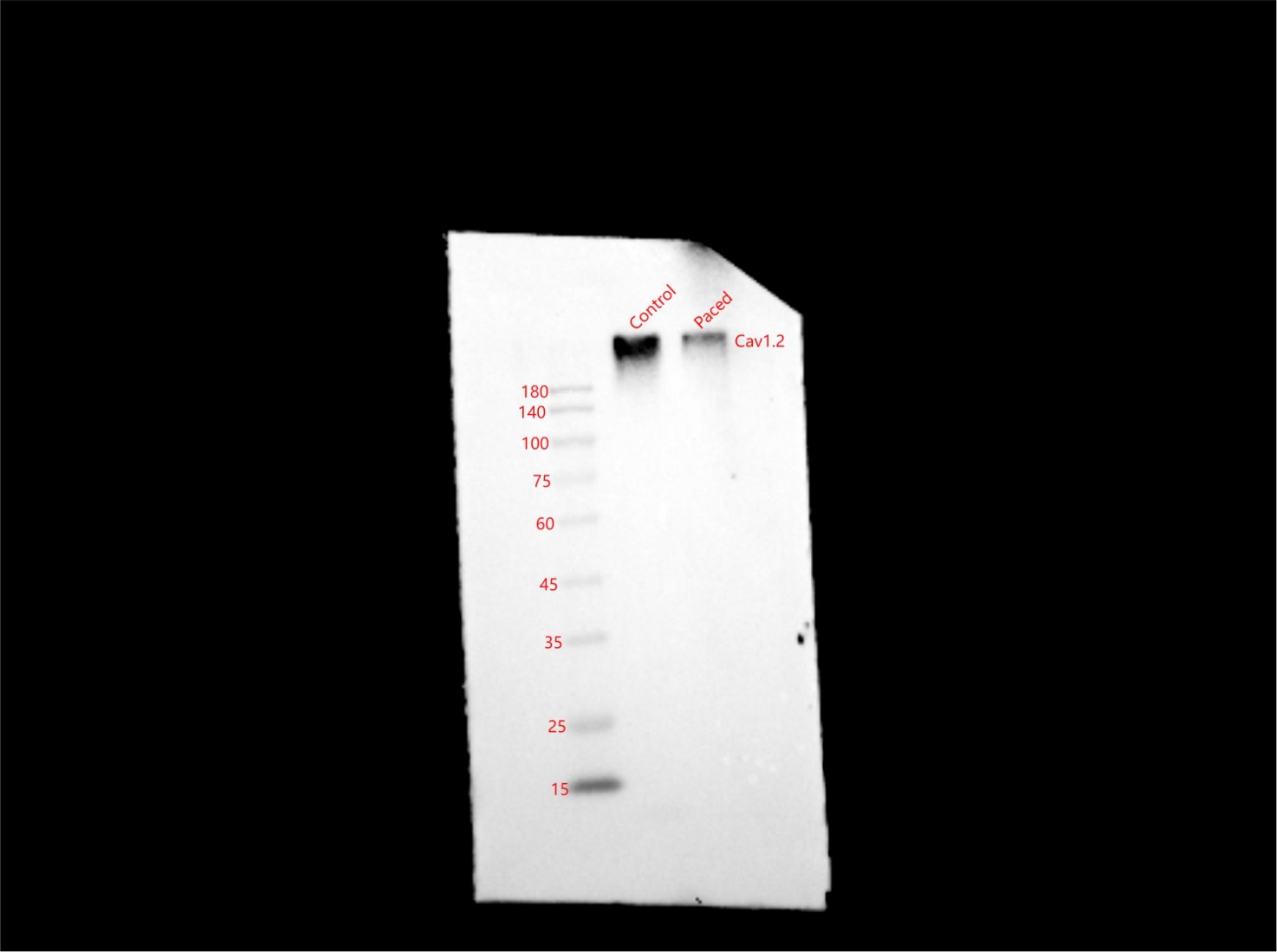


Figure 3B-1


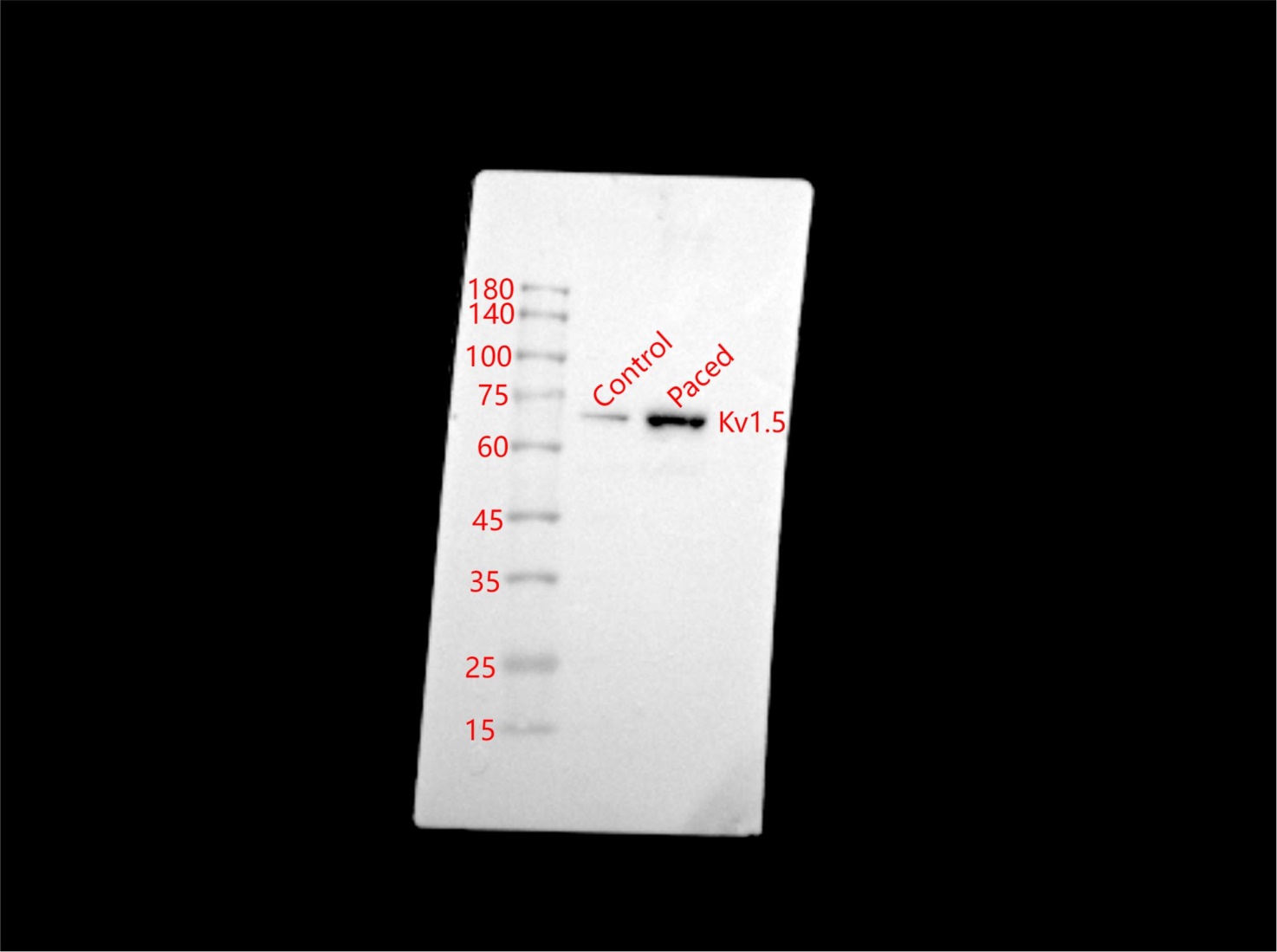


Figure 3B-2


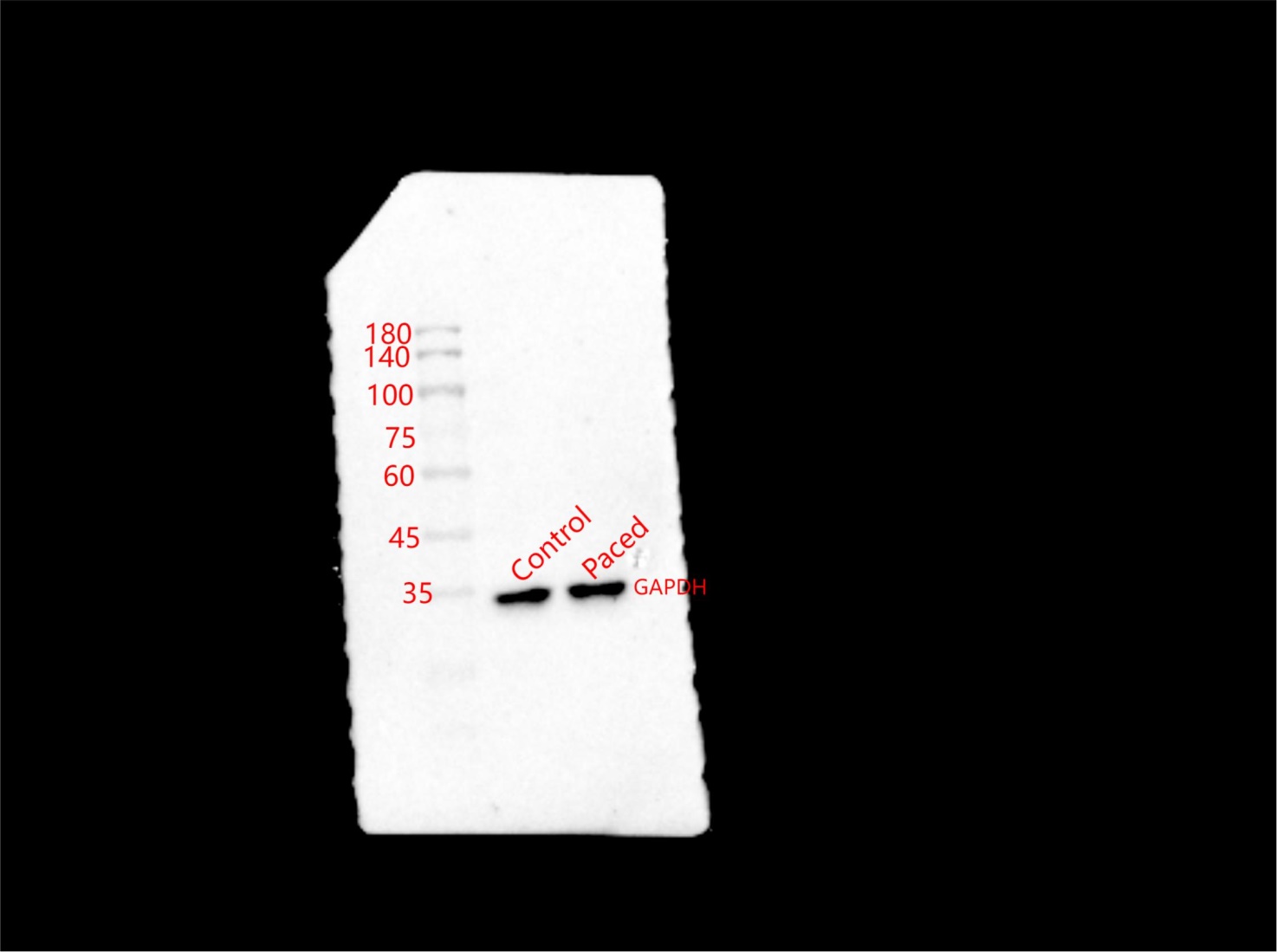


Figure 3B-3


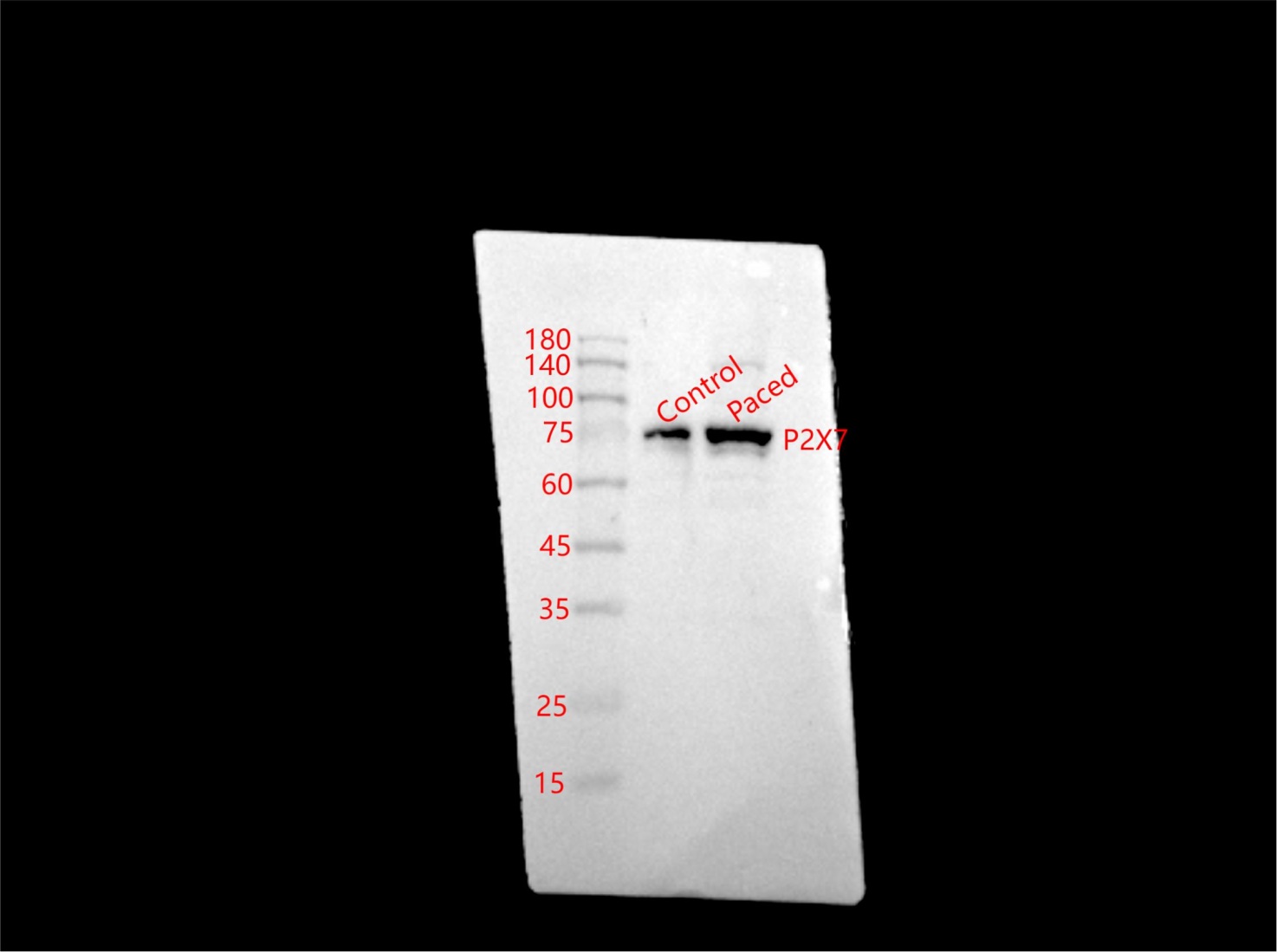


Figure 3D-1


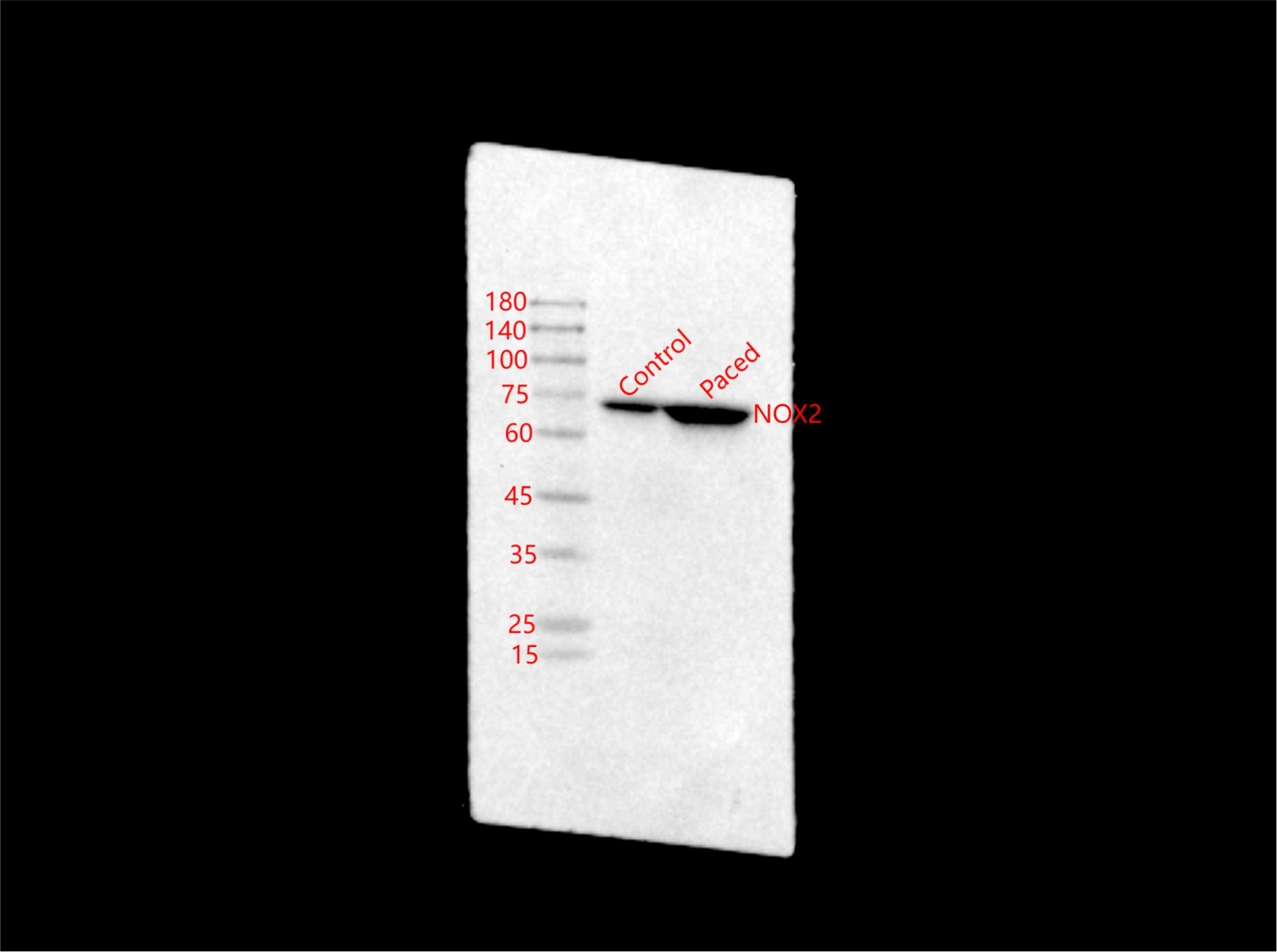


Figure 3D-2


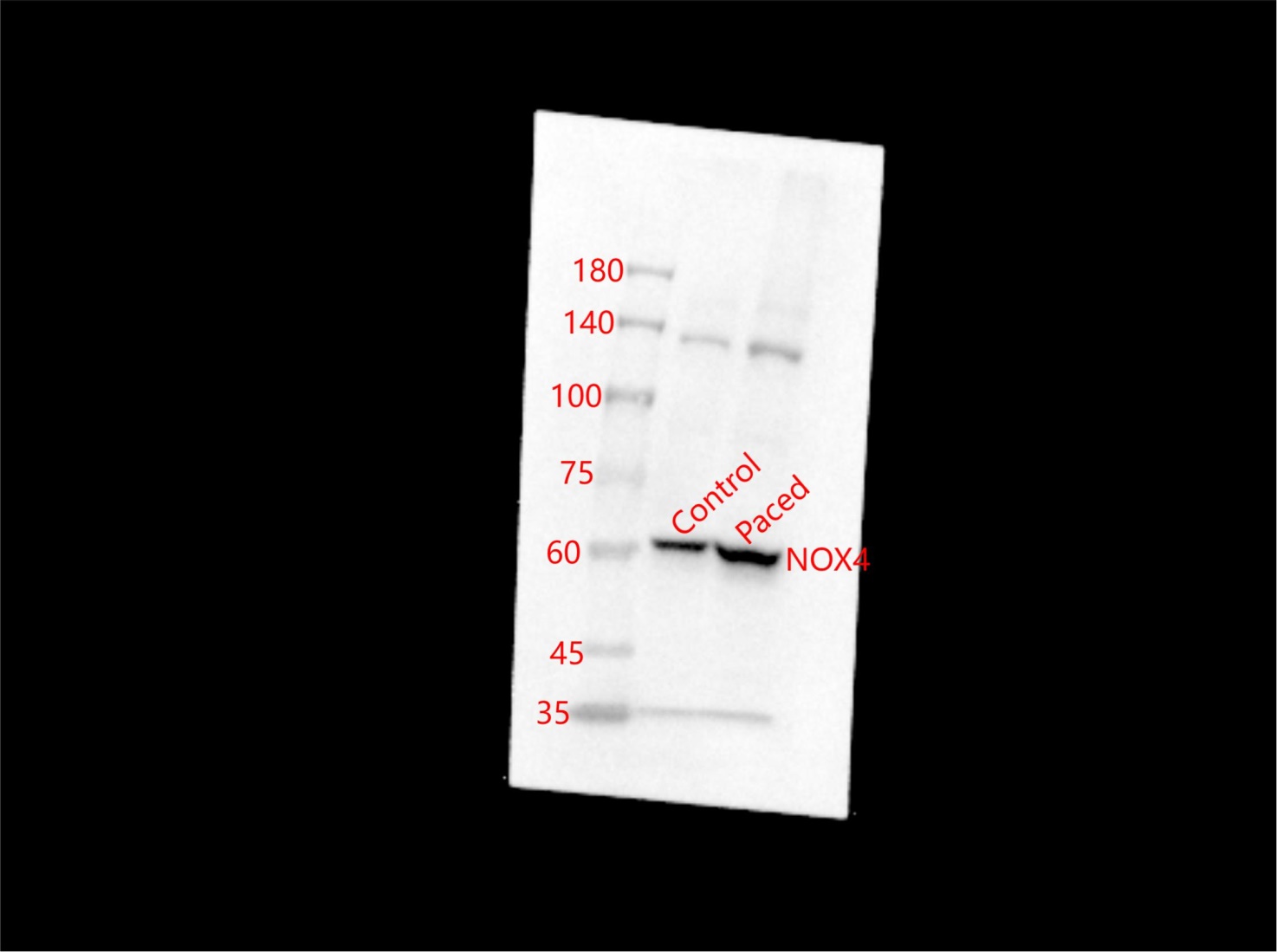


Figure 3D-3


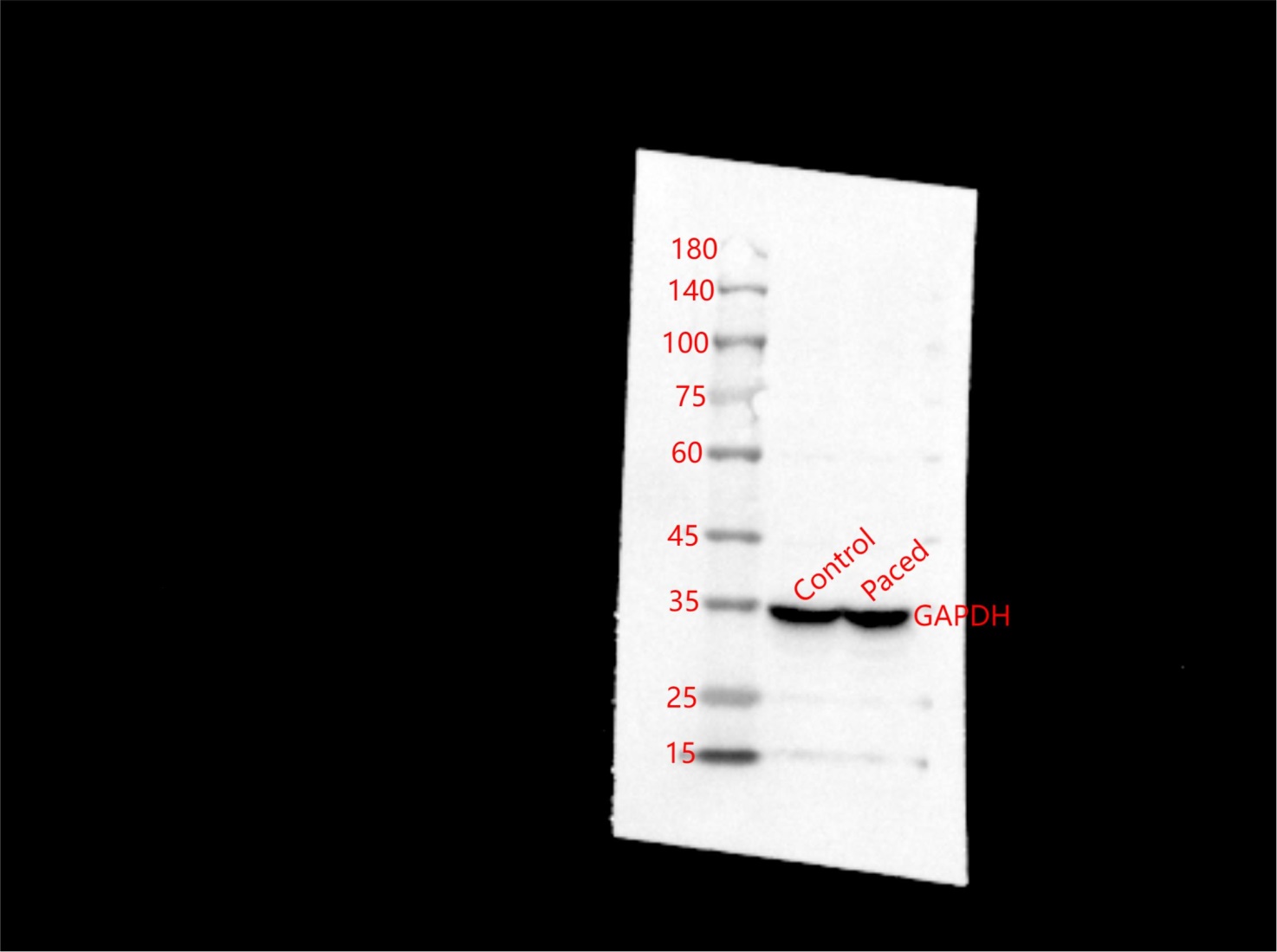


Figure 3D-4


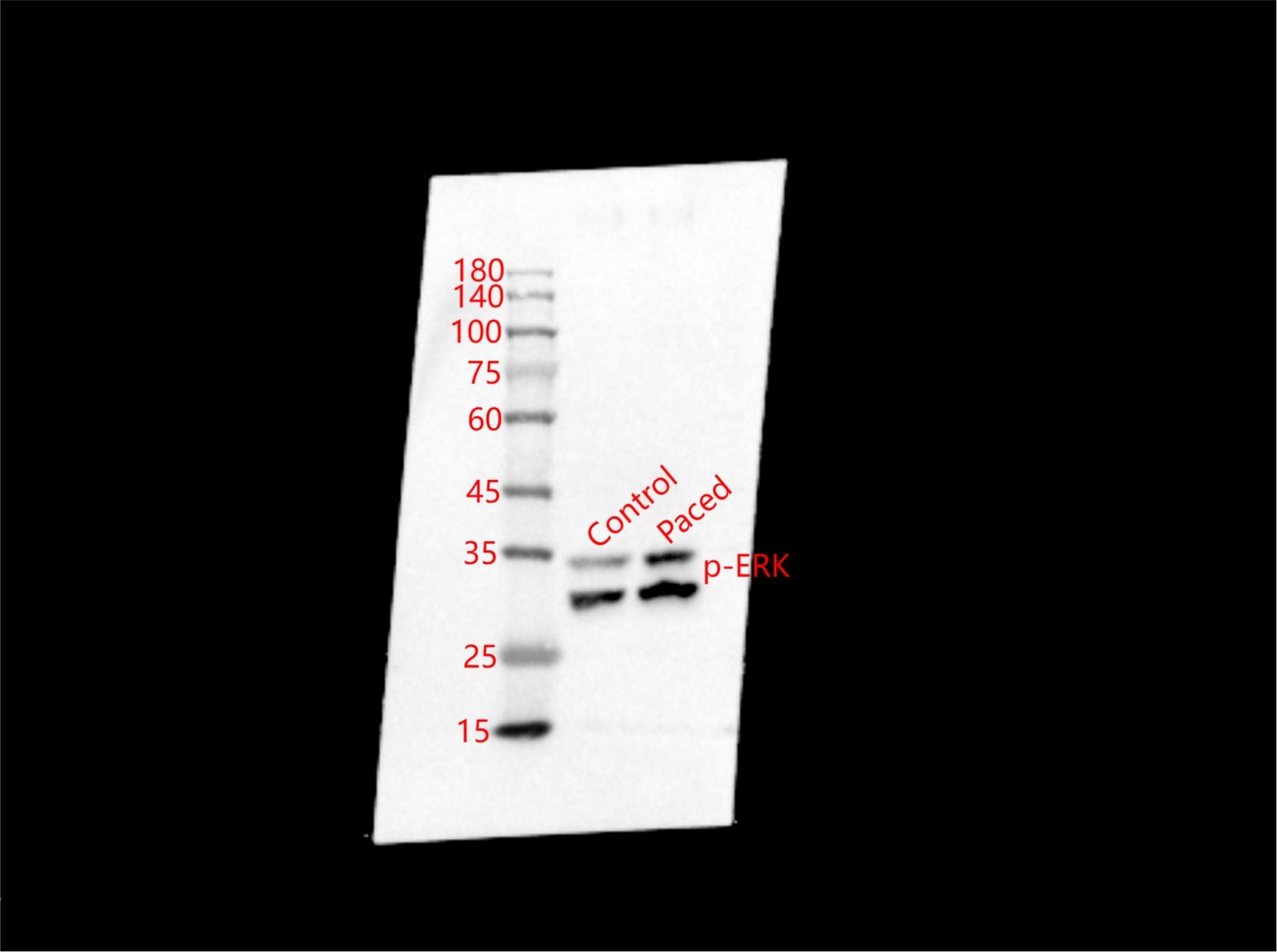


Figure 3F-1


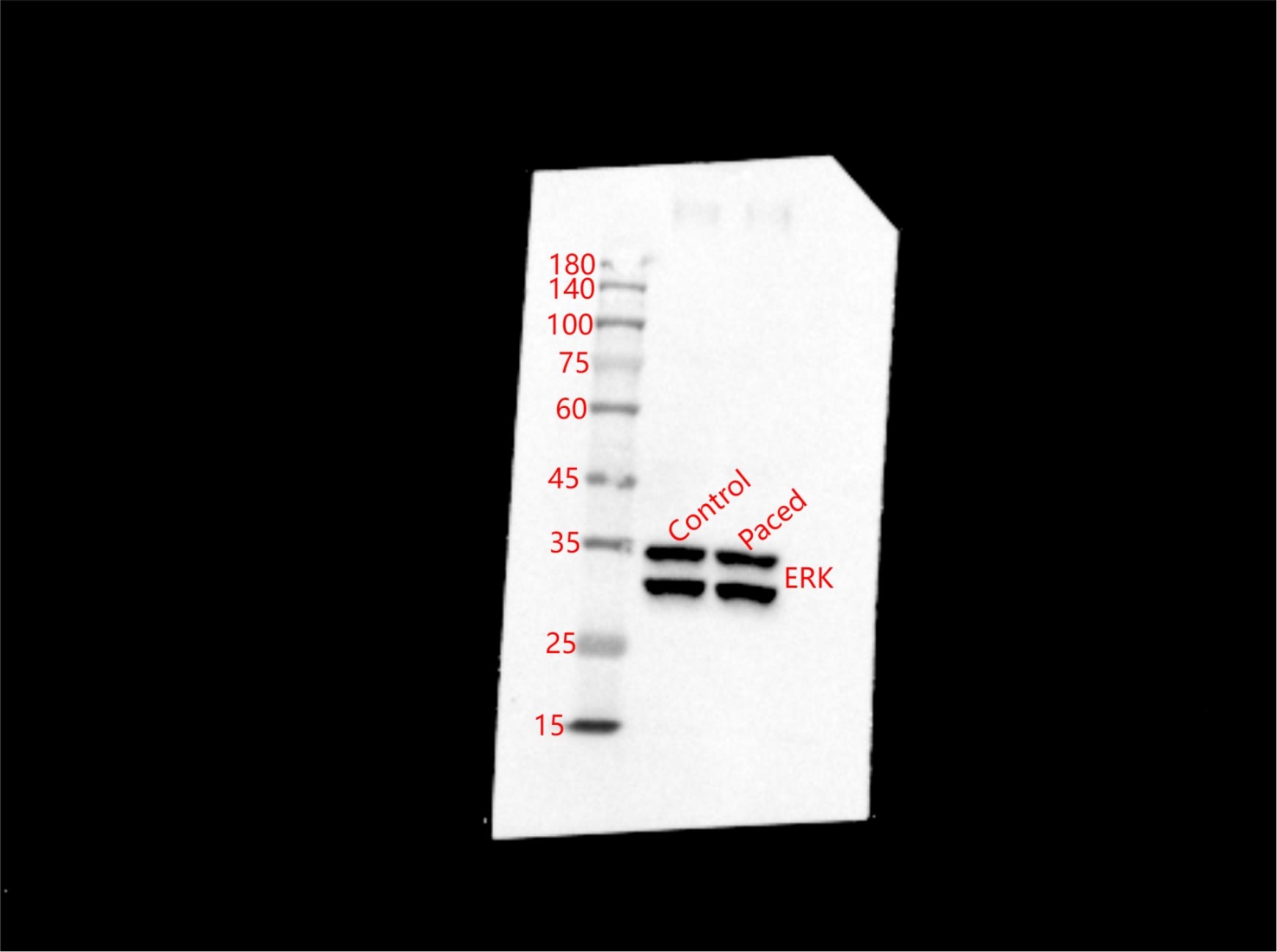


Figure 3F-2


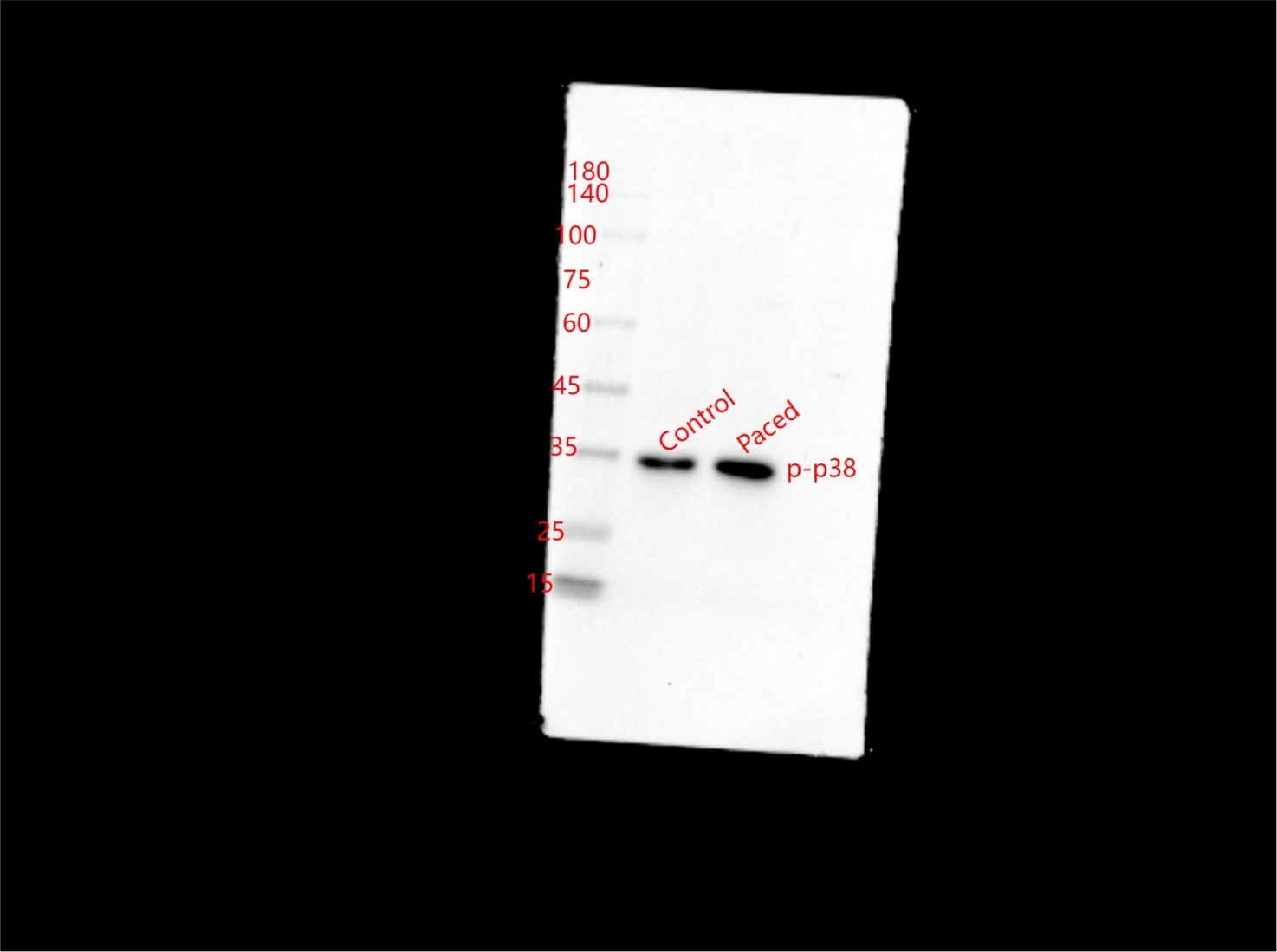


Figure 3F-3


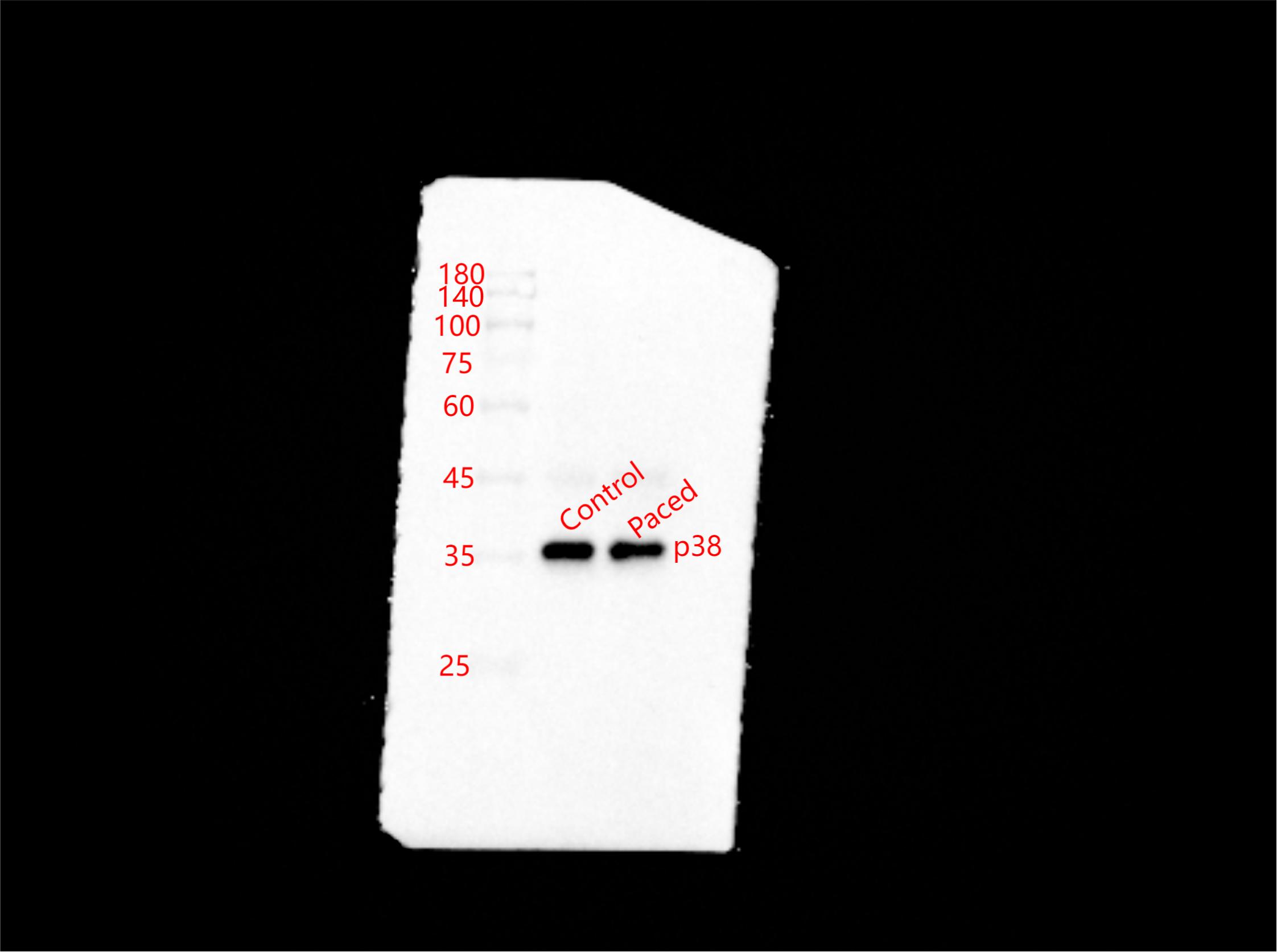


Figure 3F-4


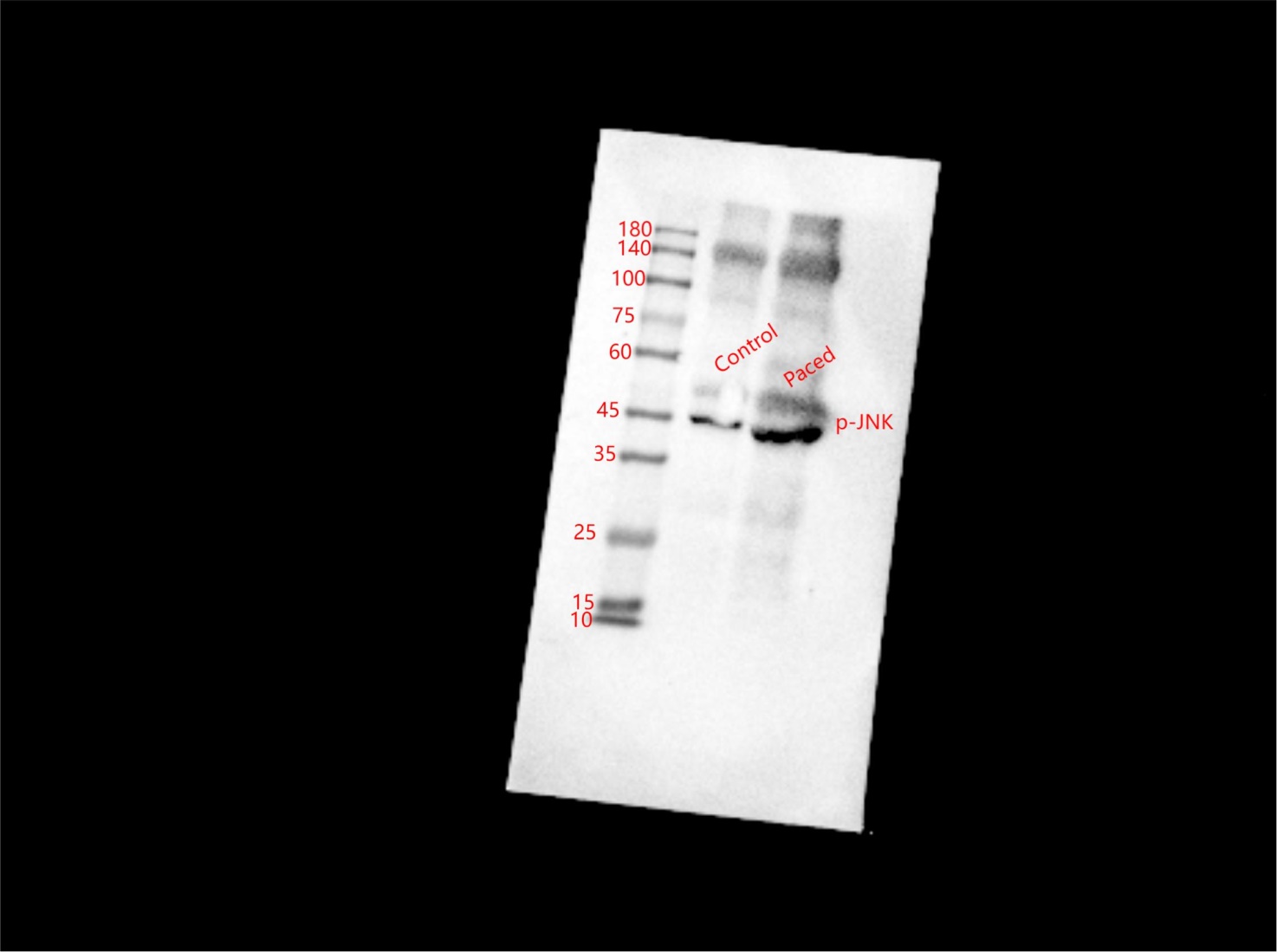


Figure 3F-5


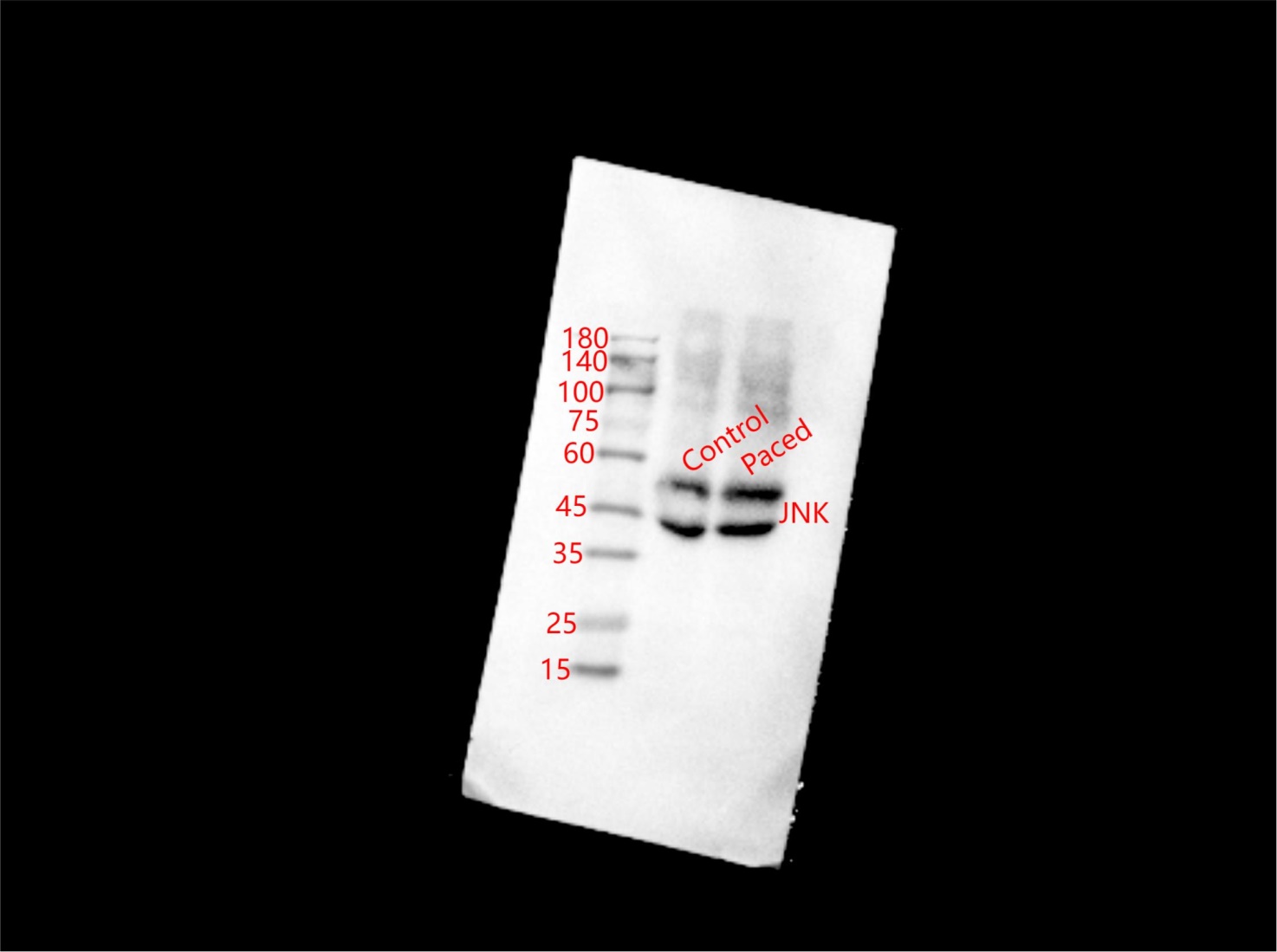


Figure 3F-6


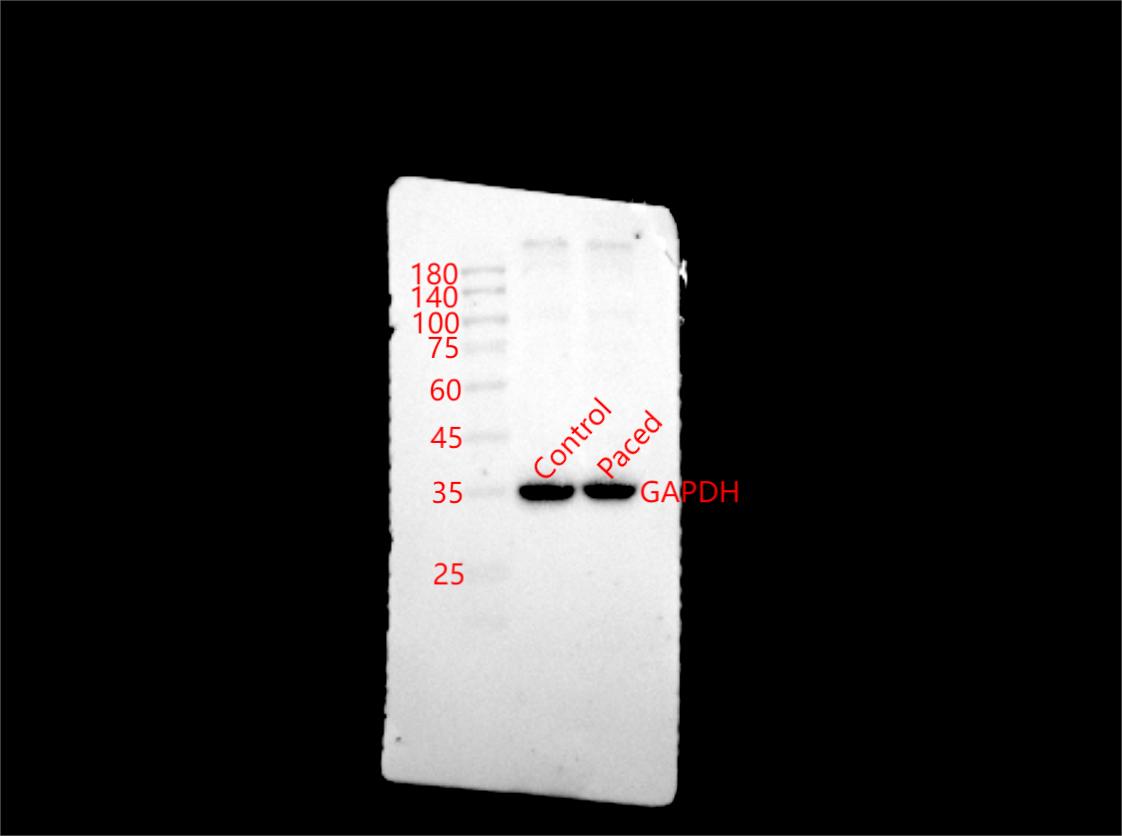


Figure 3F-7


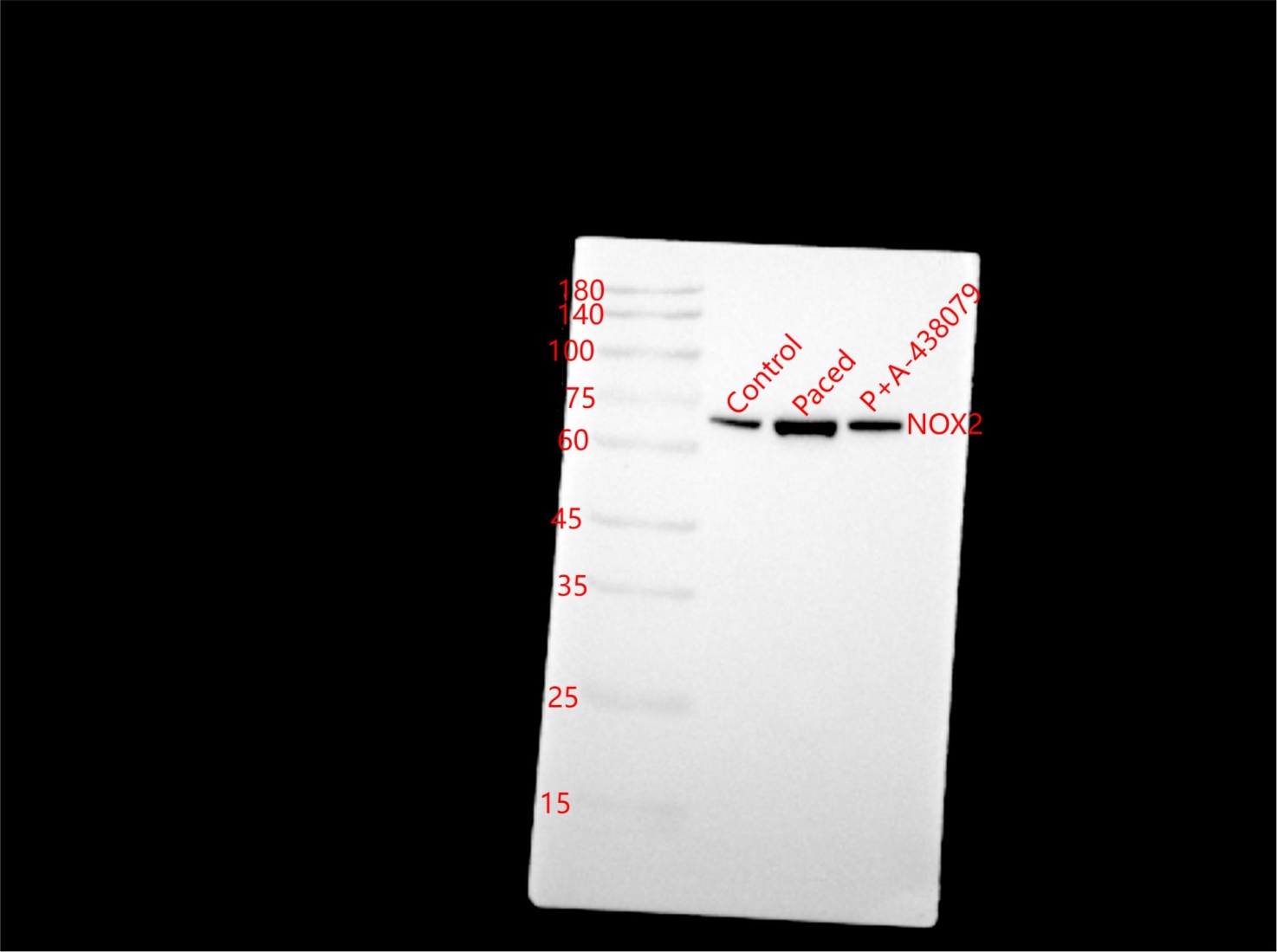


Figure 4C-1


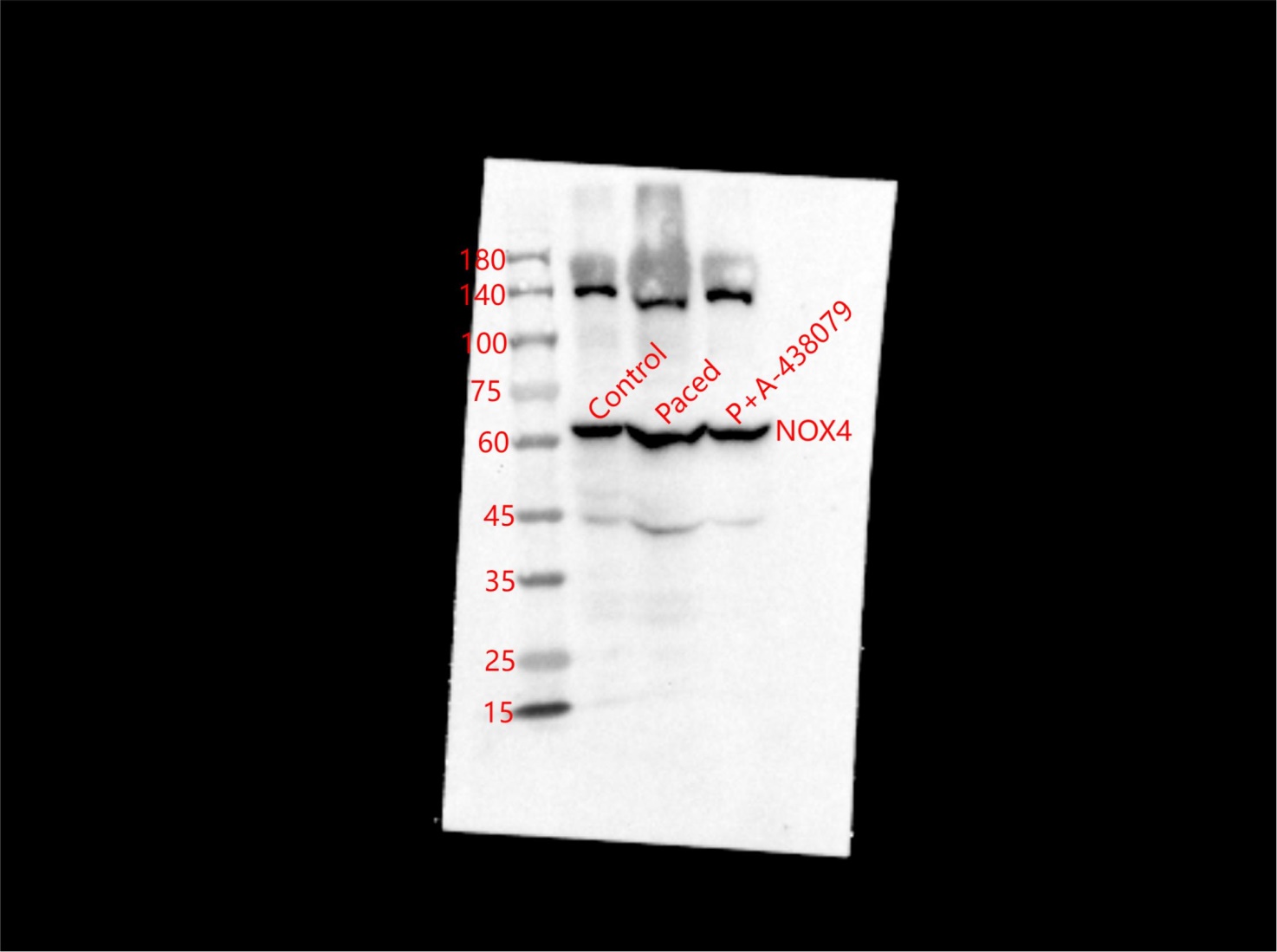


Figure 4C-2


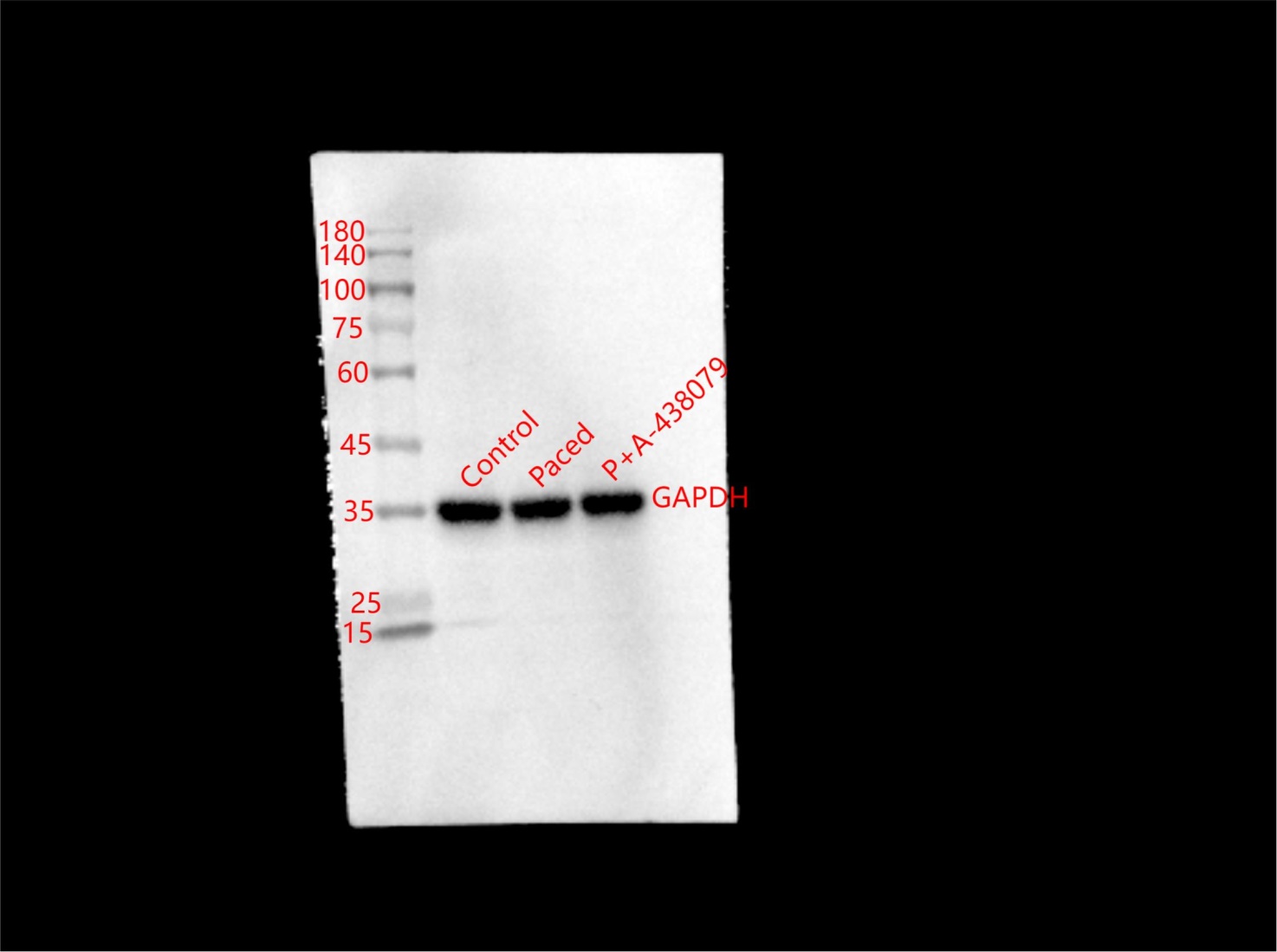


Figure 4C-3


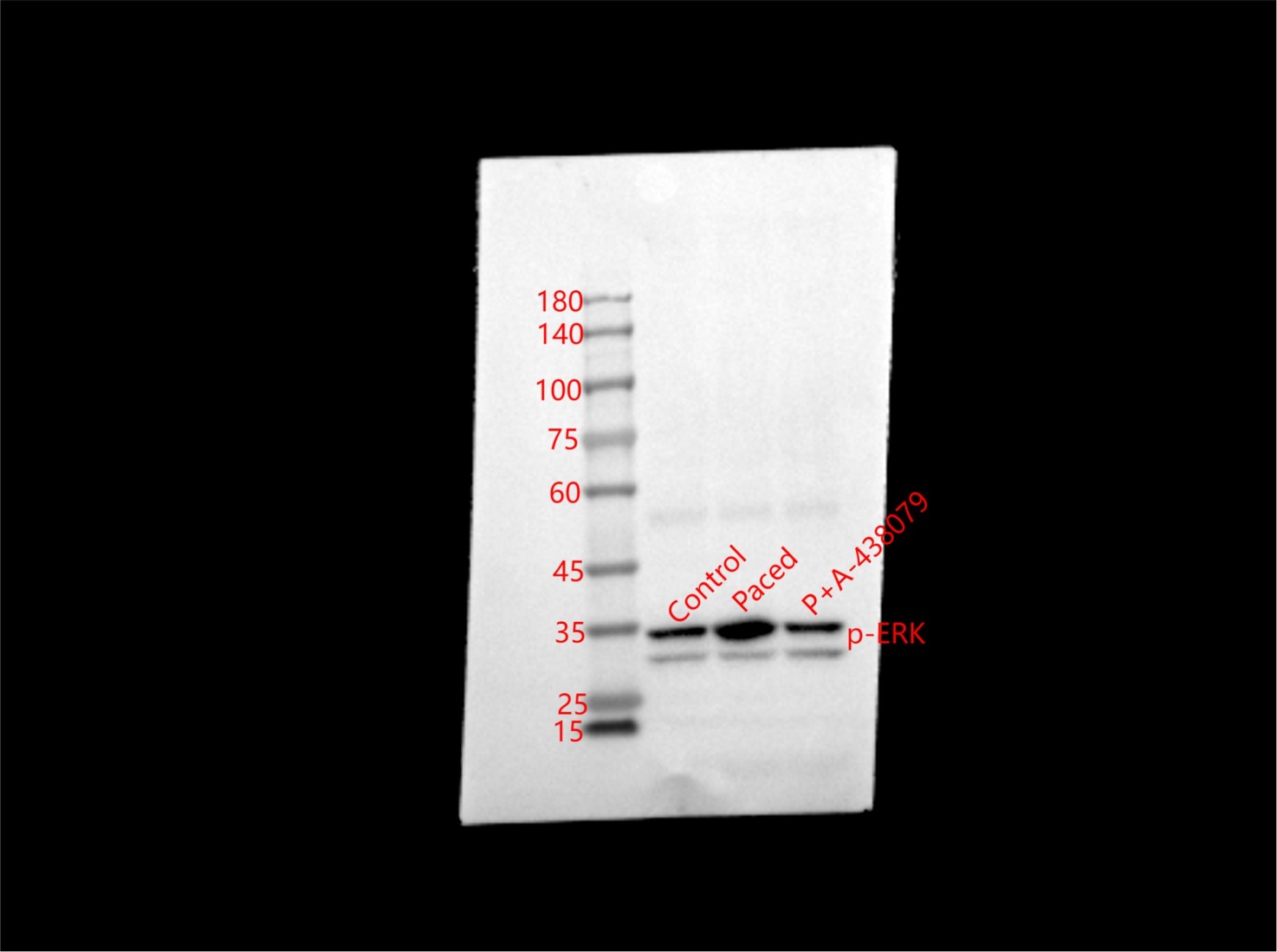


Figure 4E-1


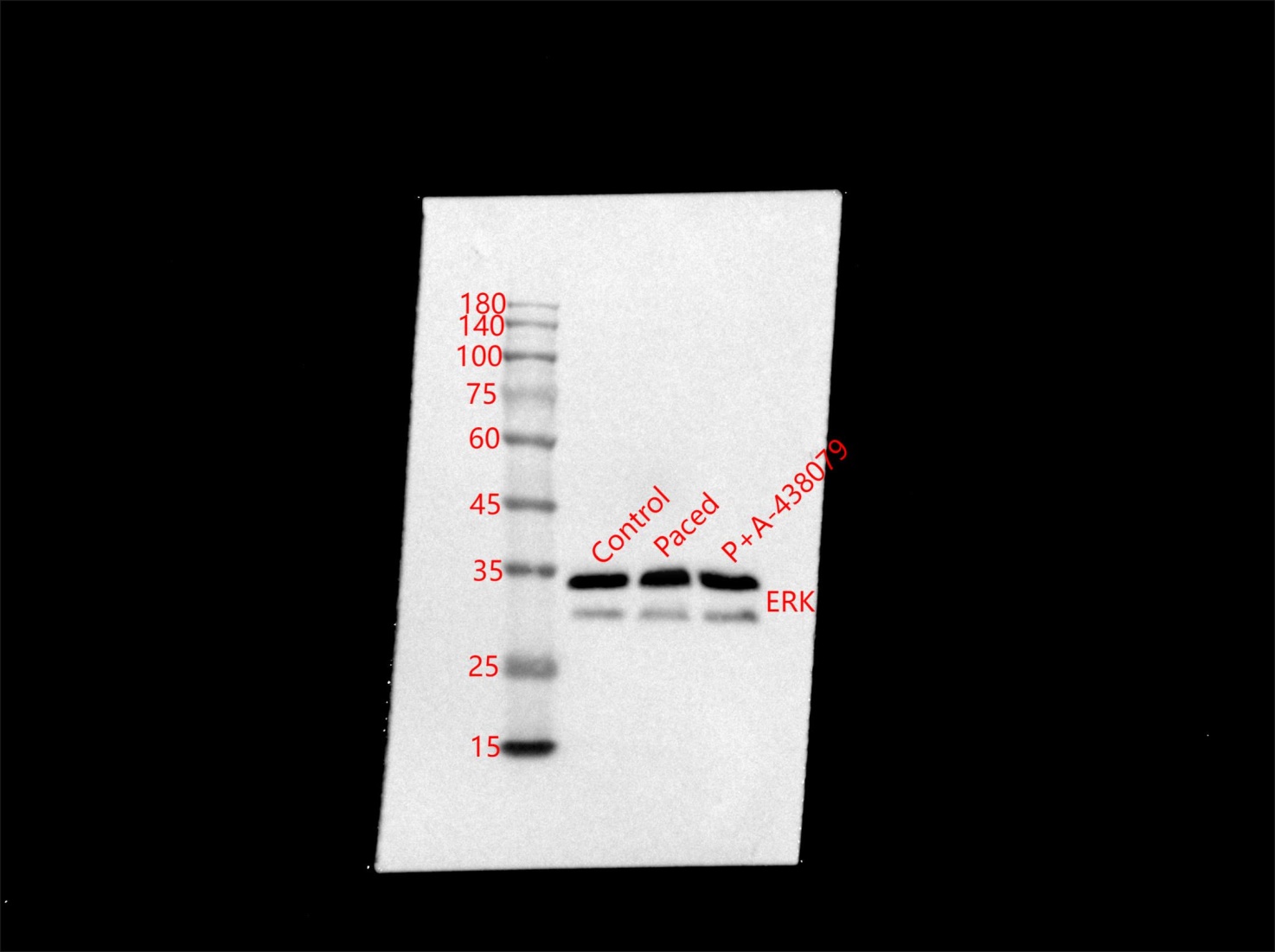


Figure 4E-2


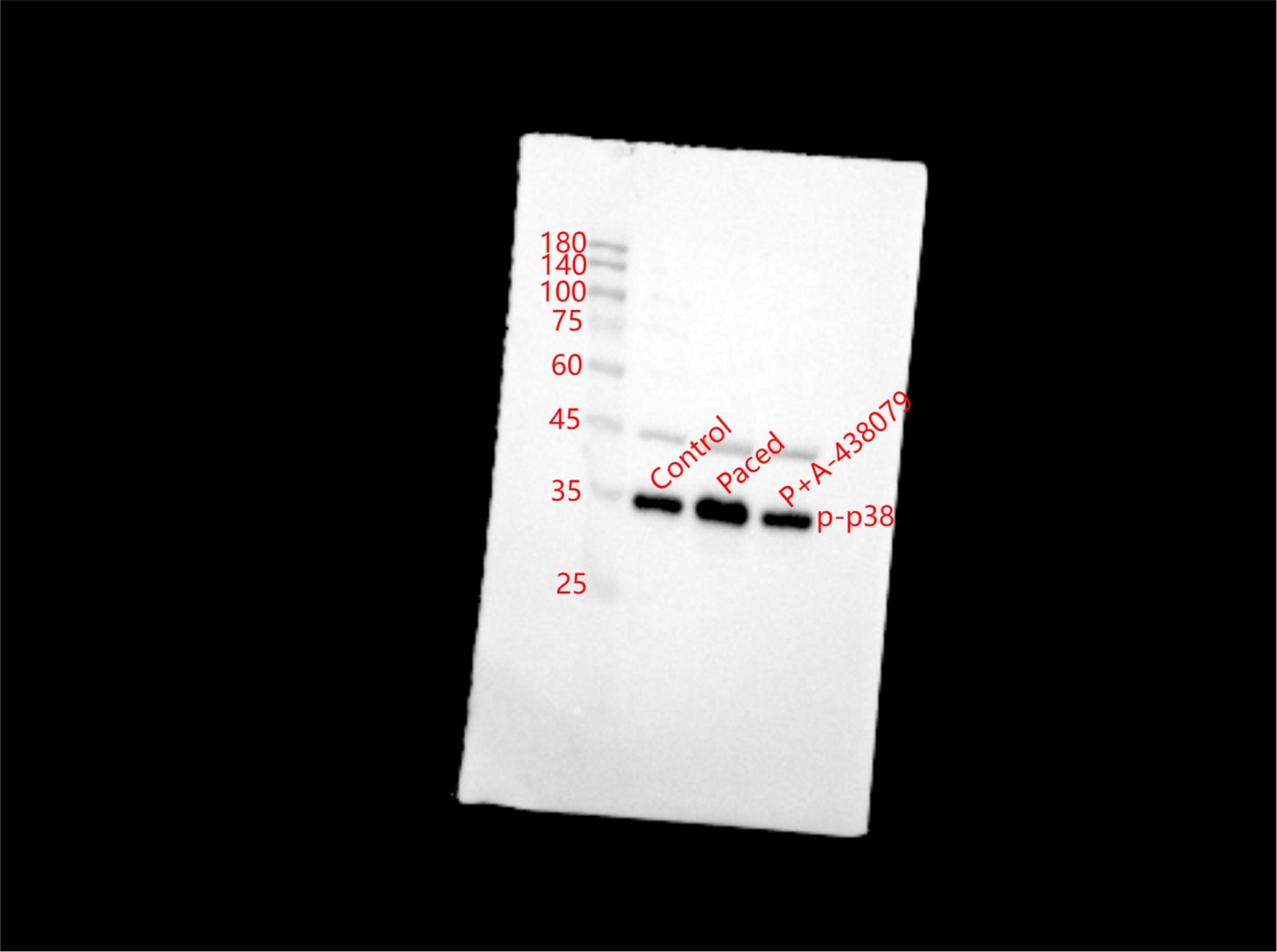


Figure 4E-3


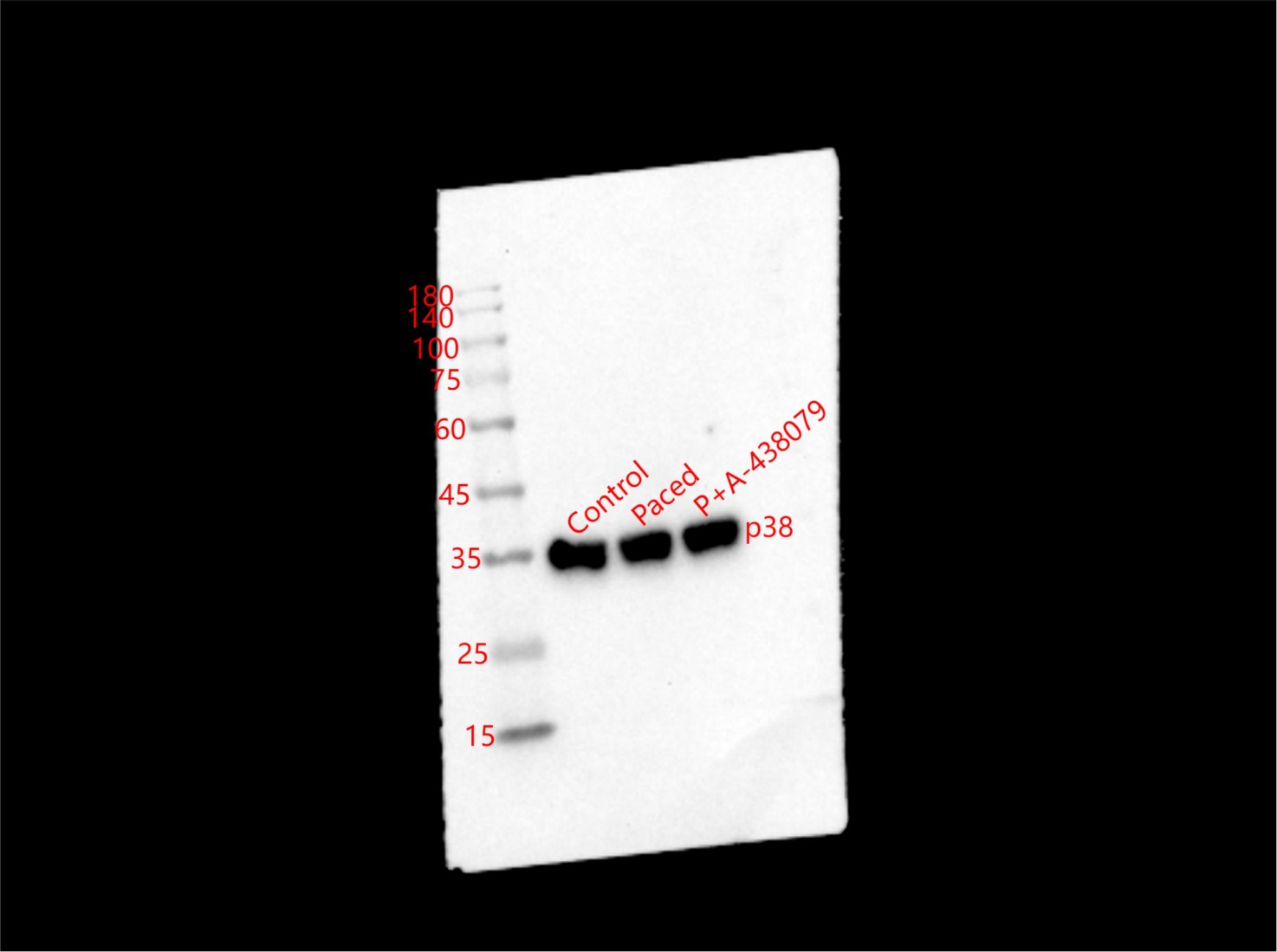


Figure 4E-4


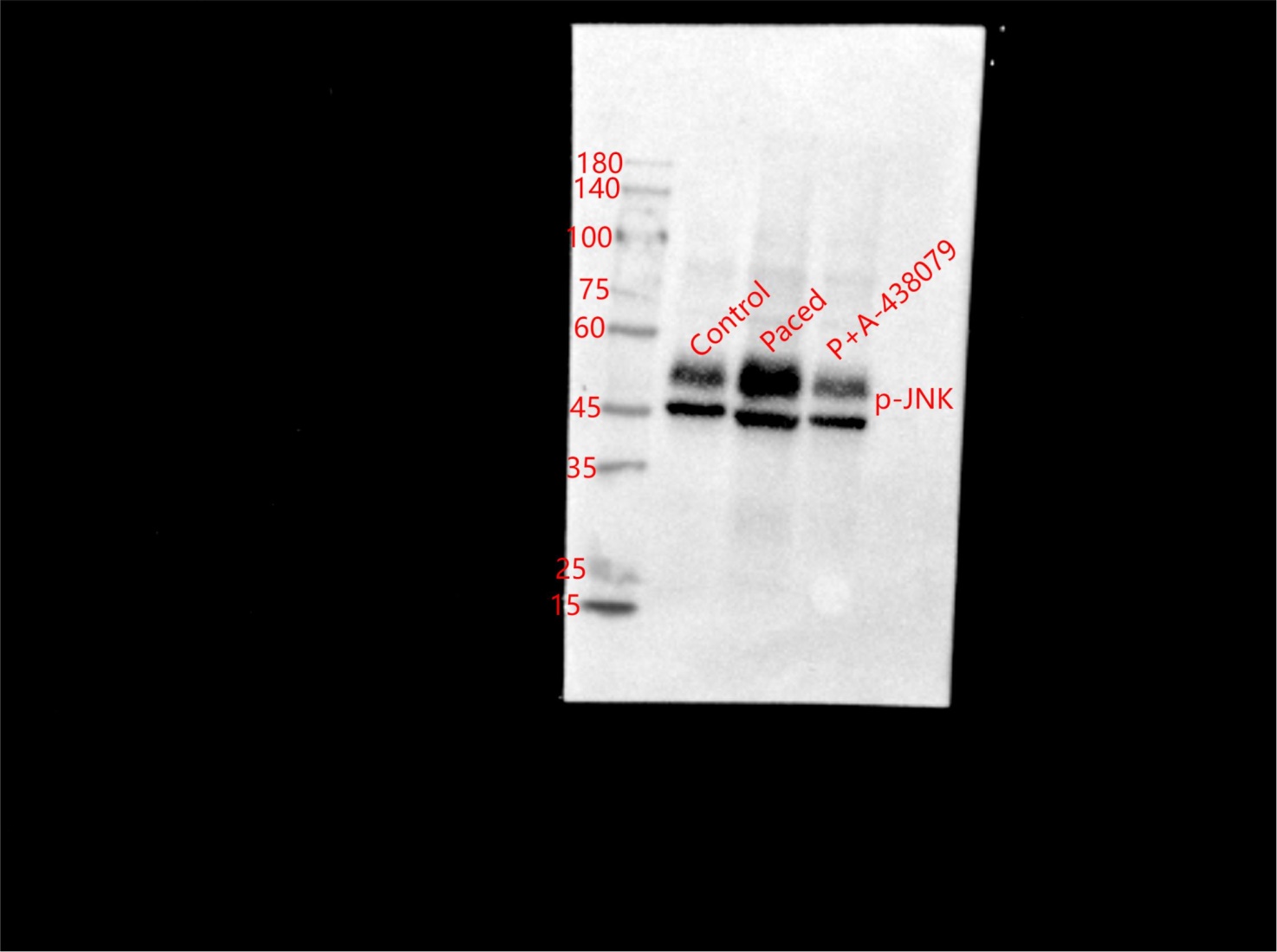


Figure 4E-5


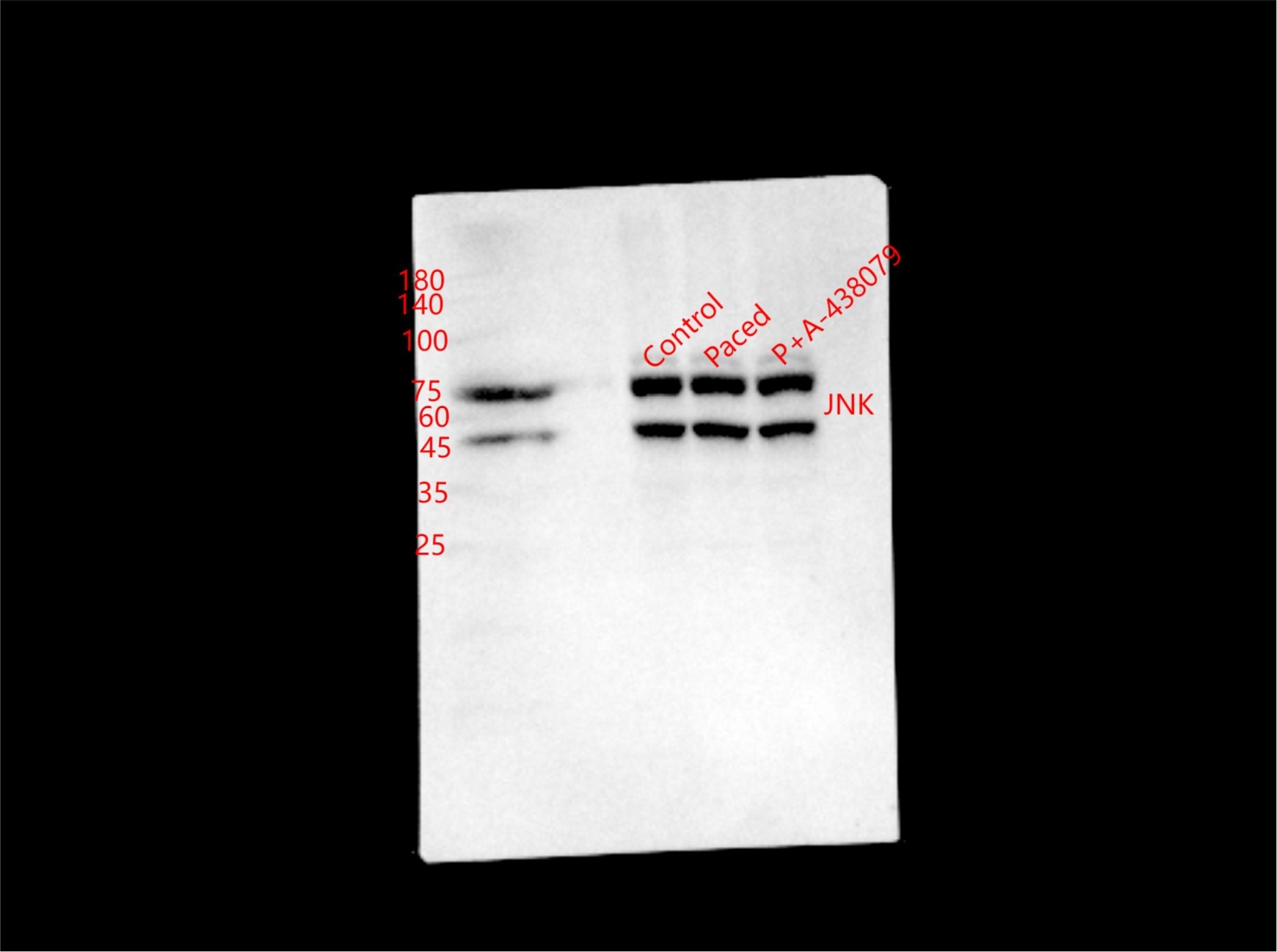


Figure 4E-6


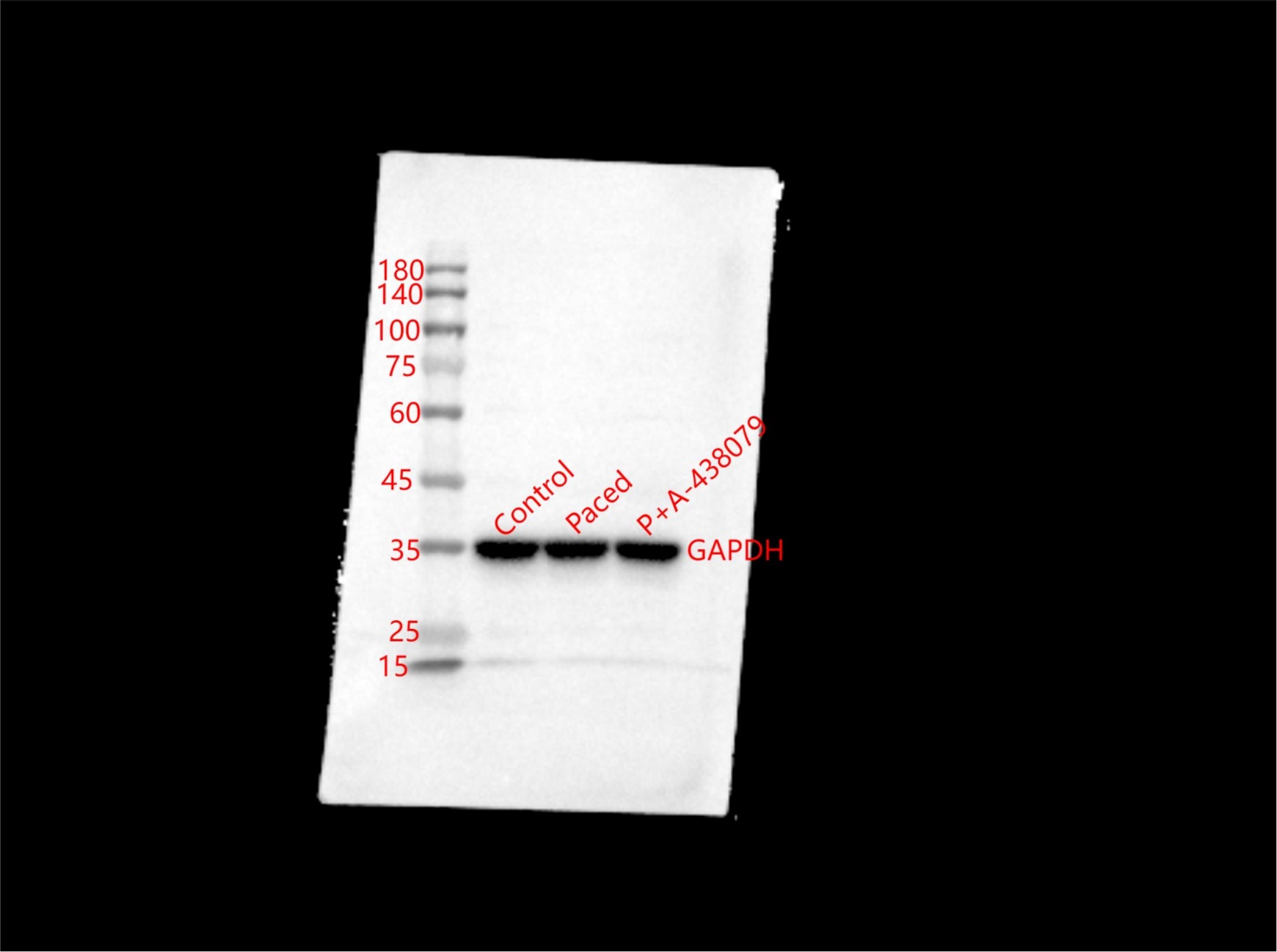


Figure 4E-7


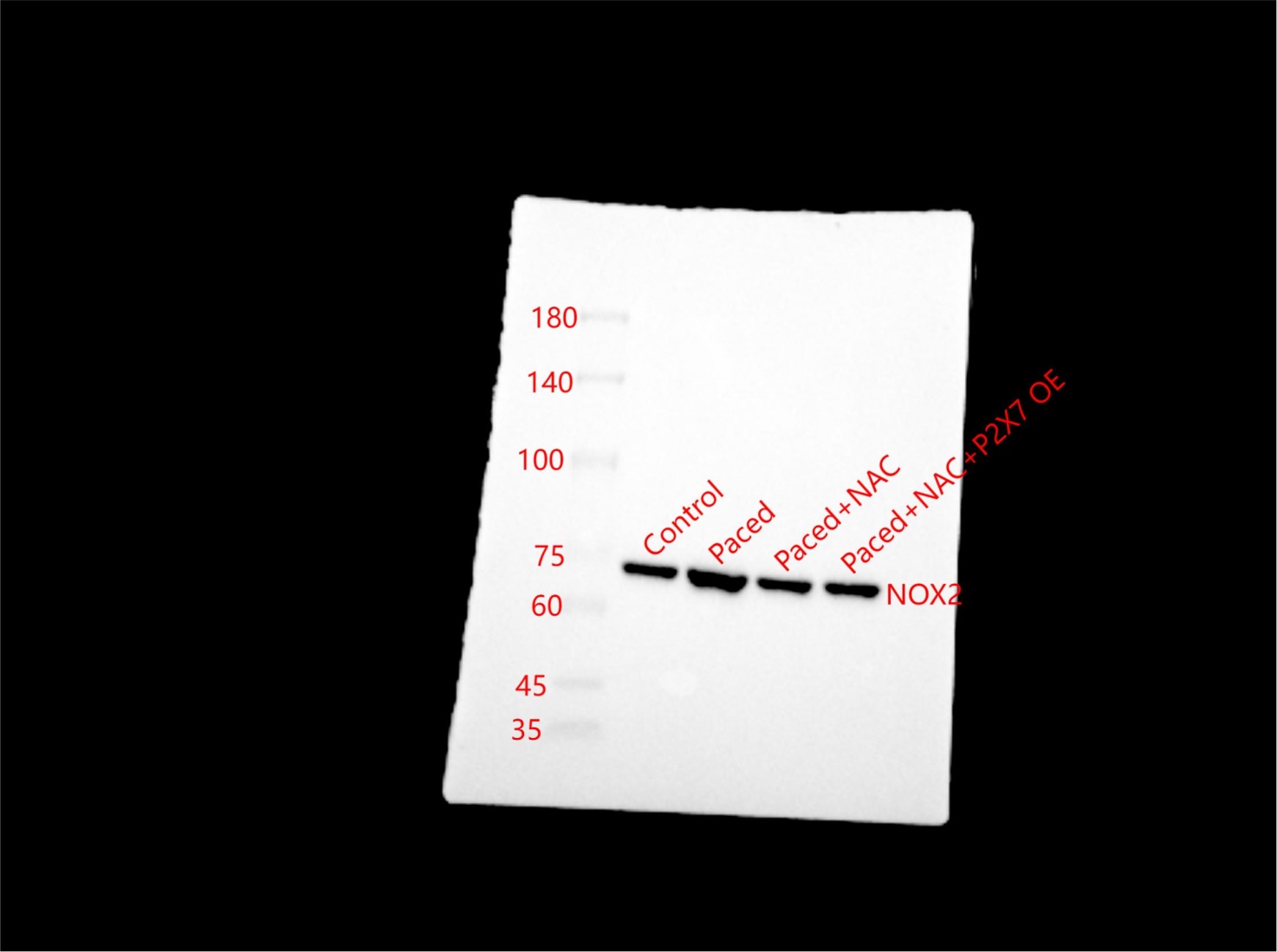


Figure 5D-1


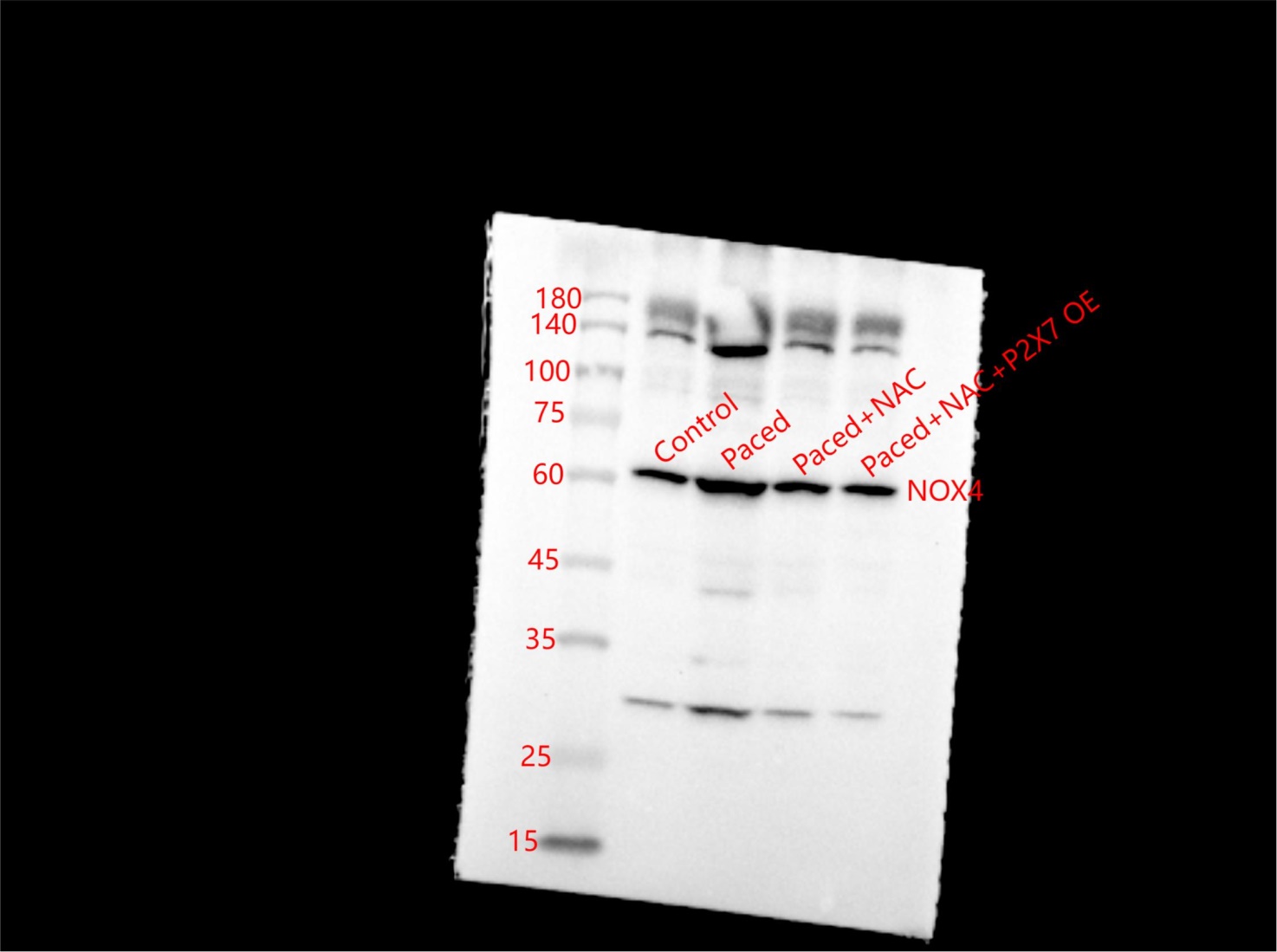


Figure 5D-2


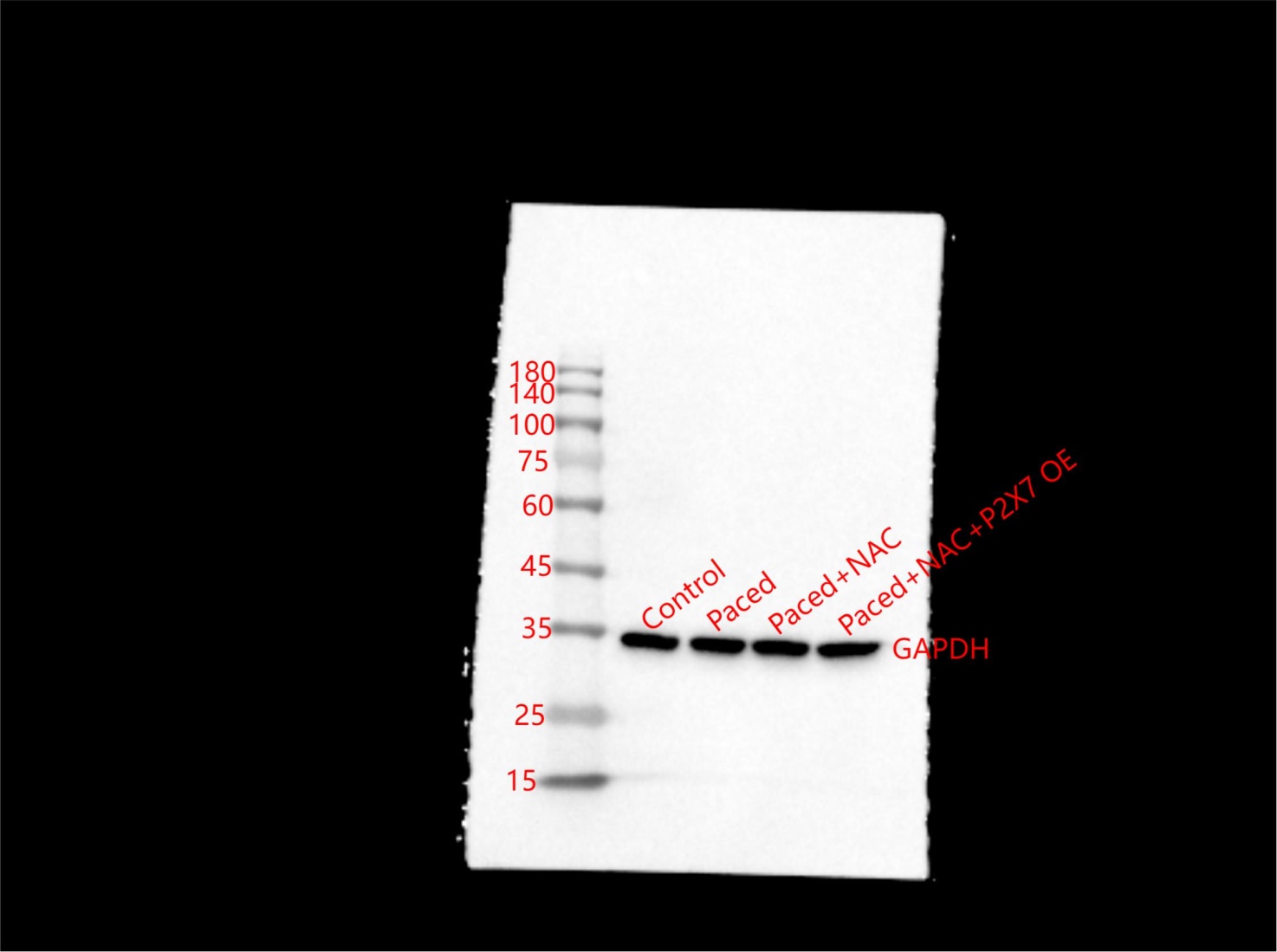


Figure 5D-3


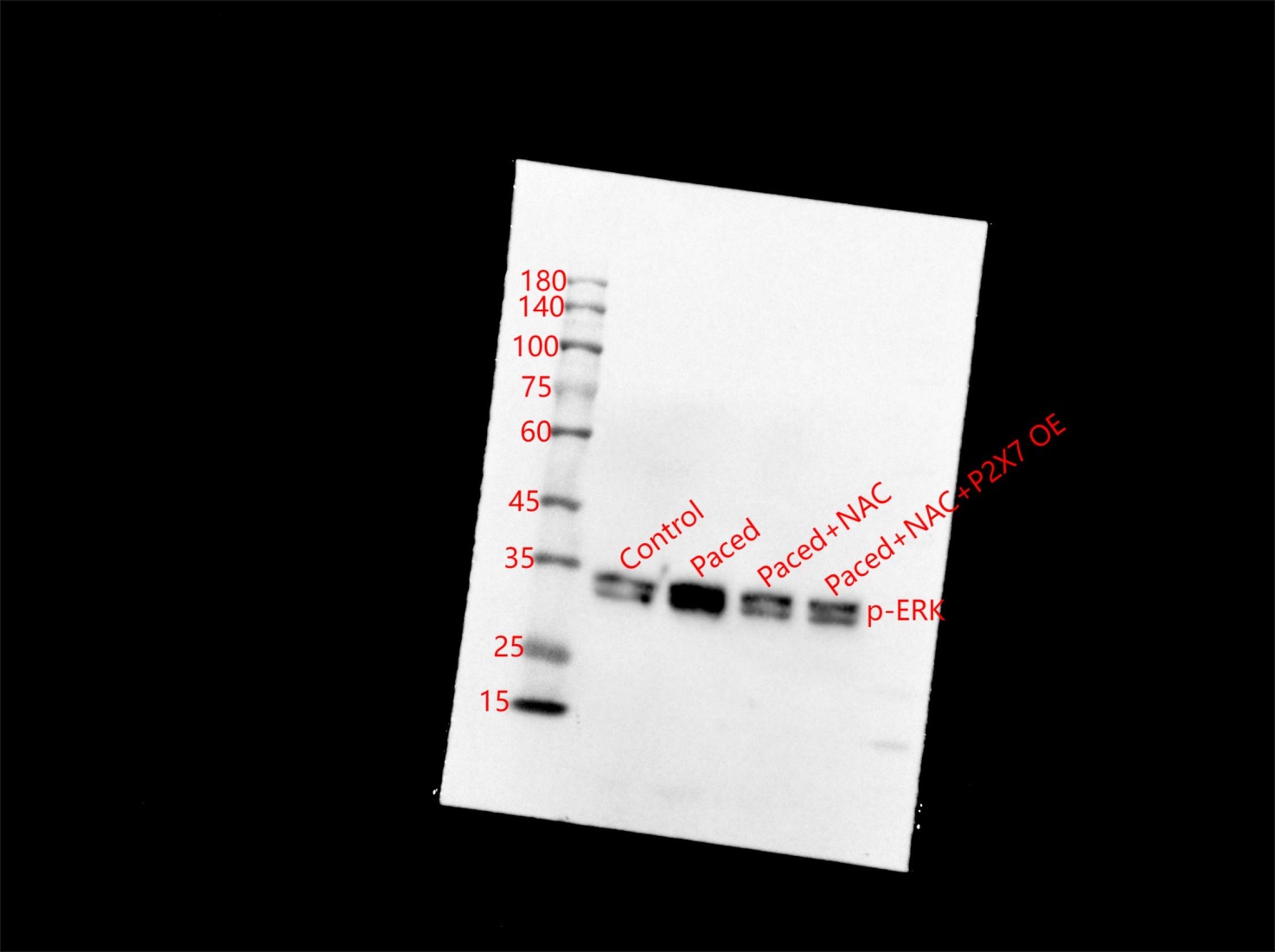


Figure 5E-1


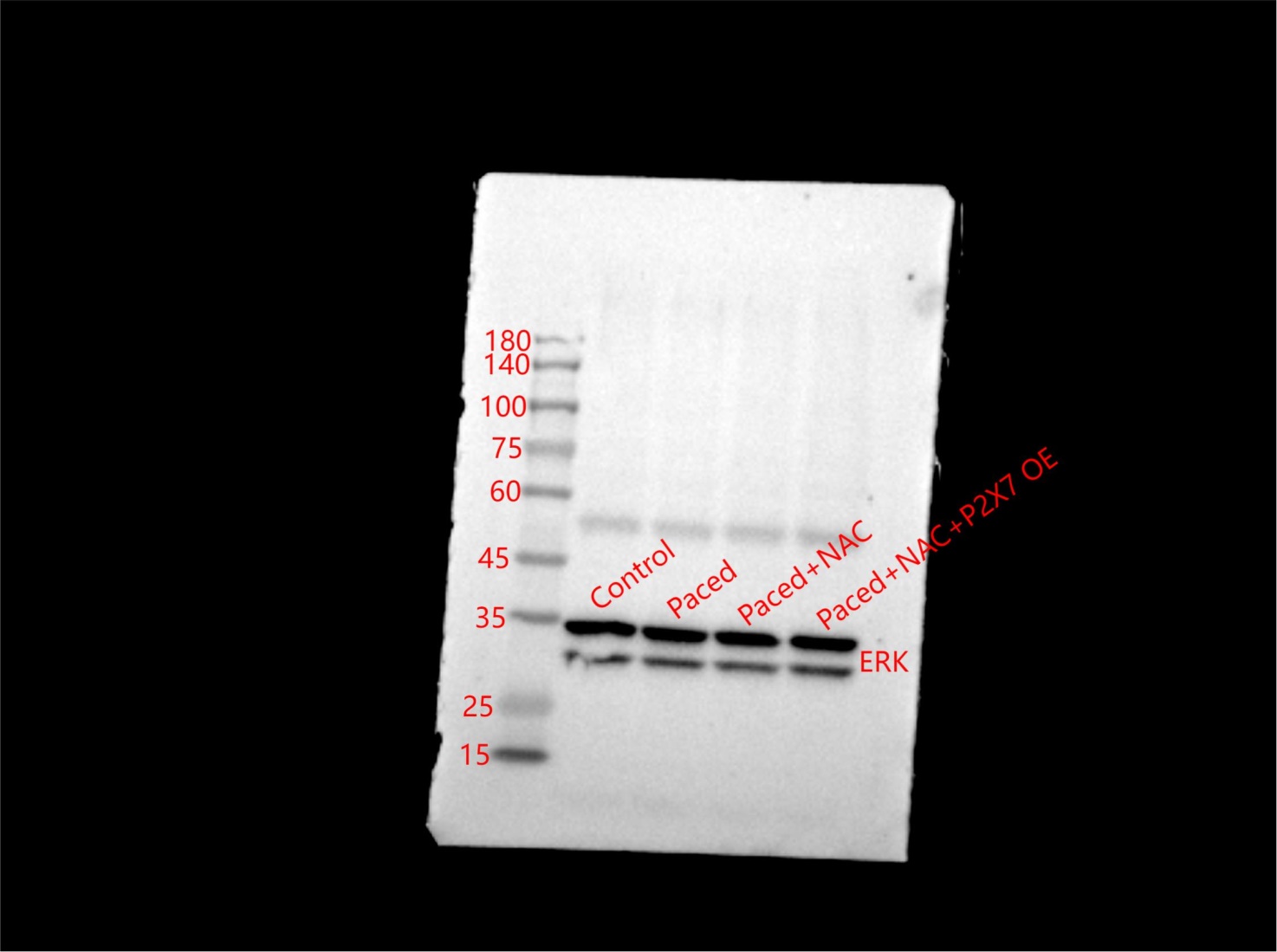


Figure 5E-2


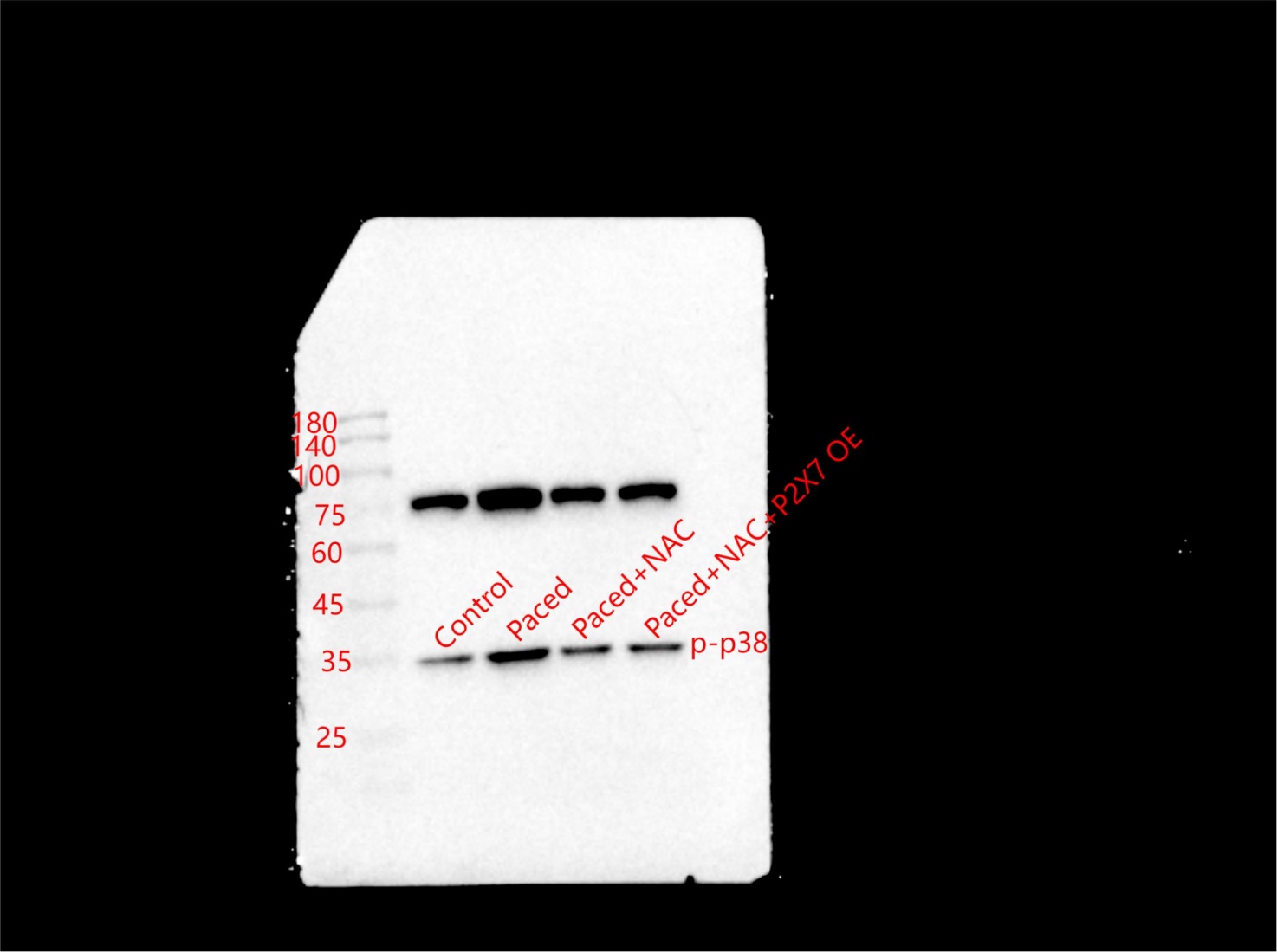


Figure 5E-3


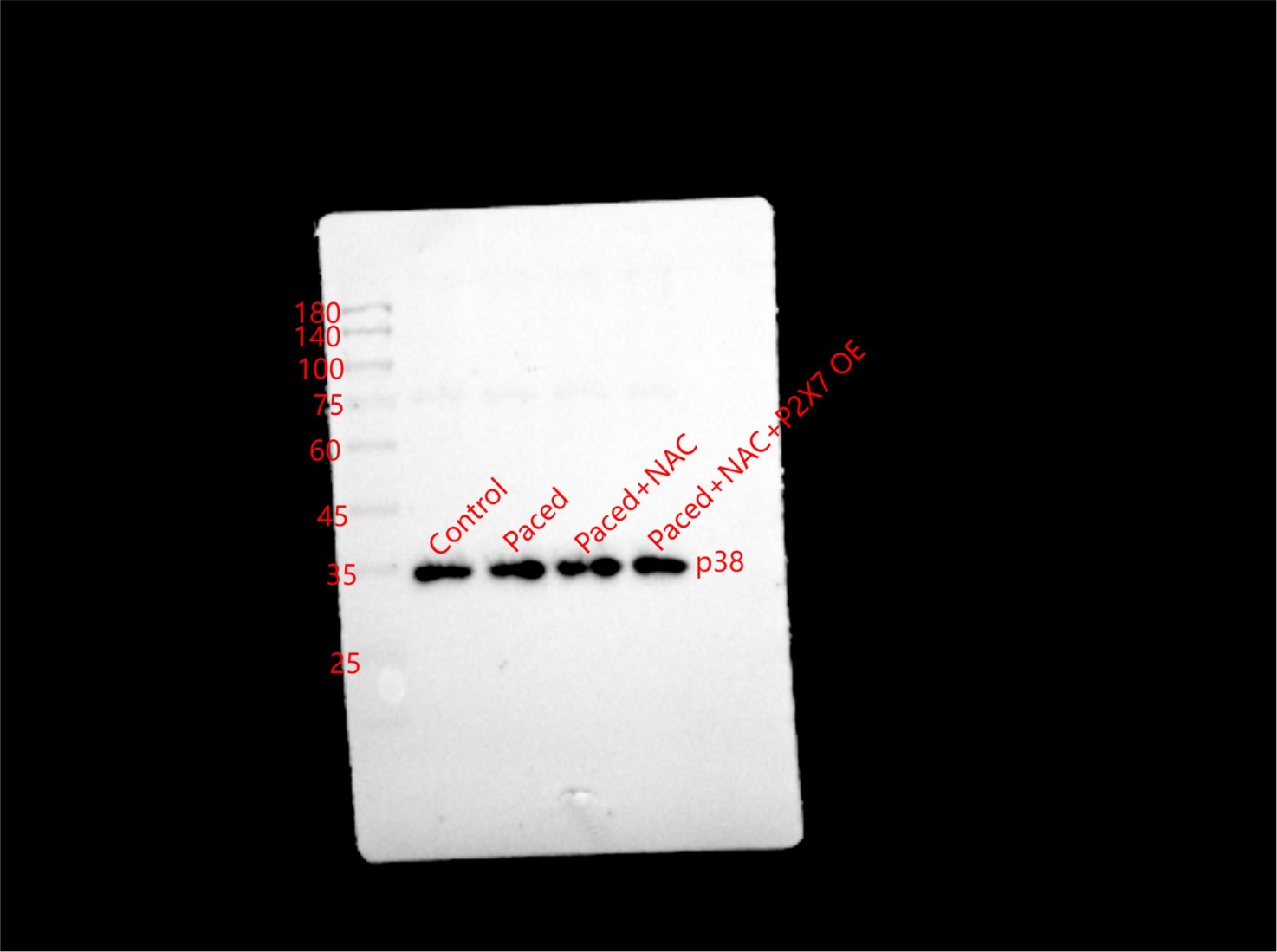


Figure 5E-4


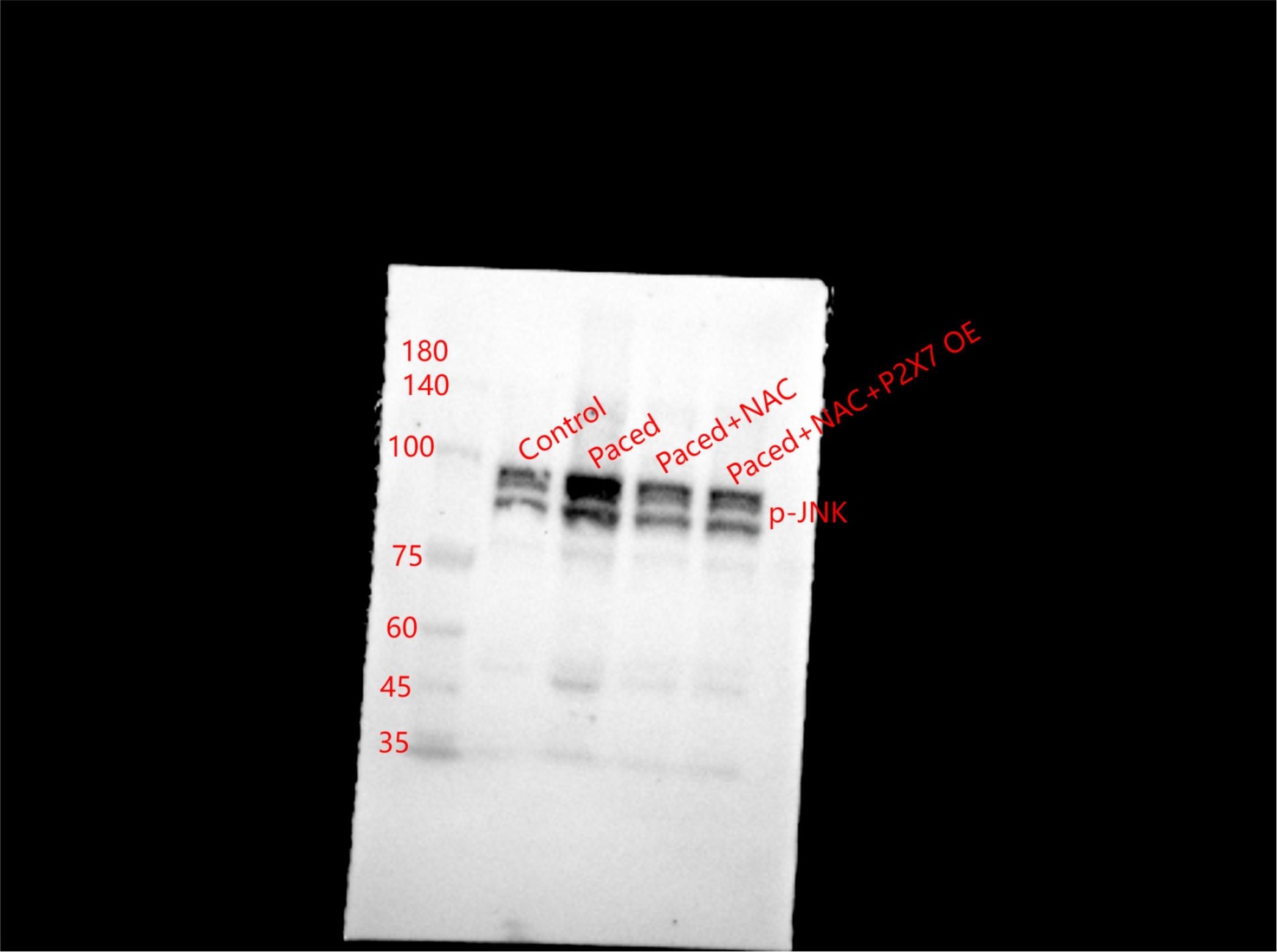


Figure 5E-5


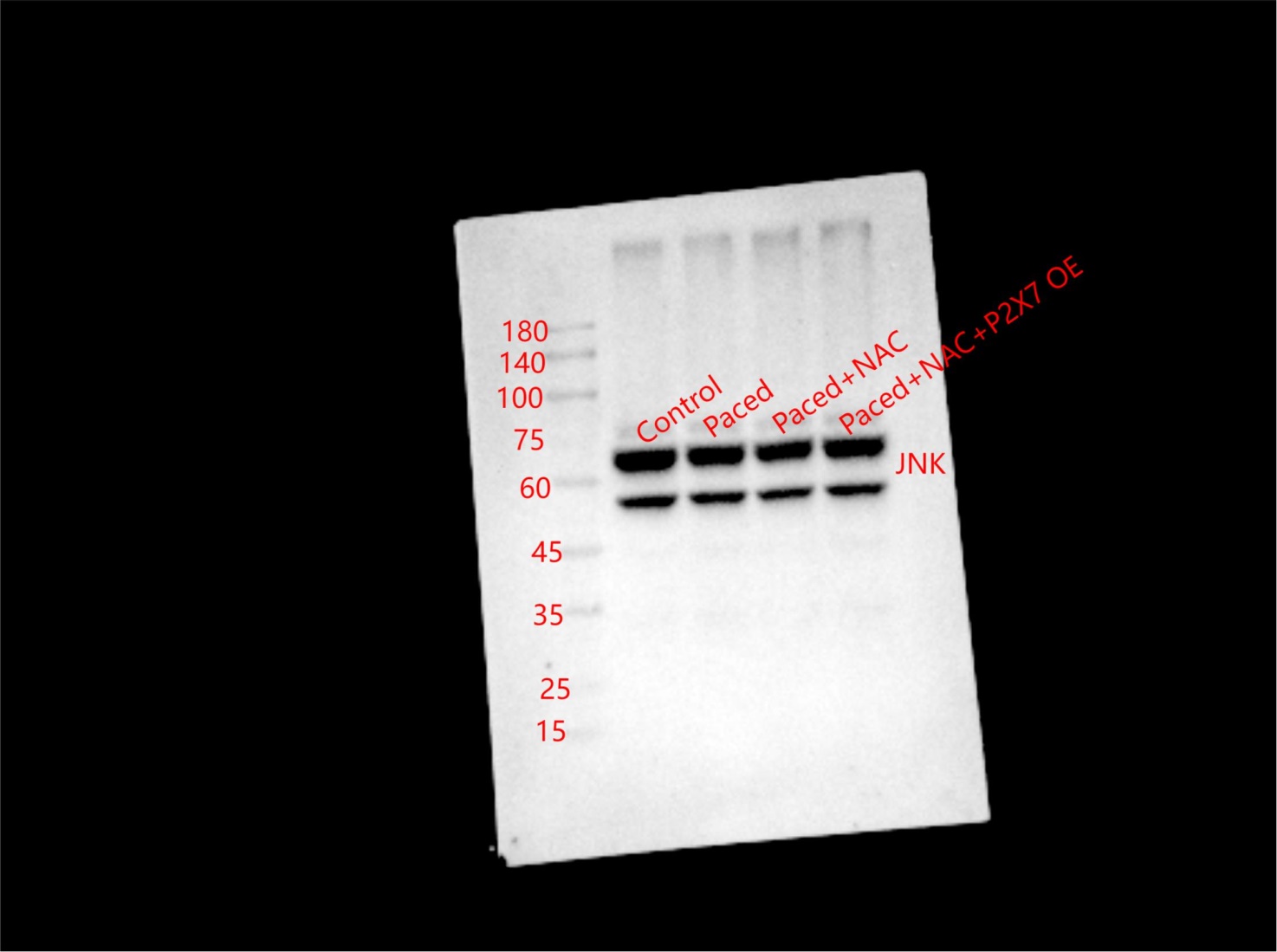


Figure 5E-6


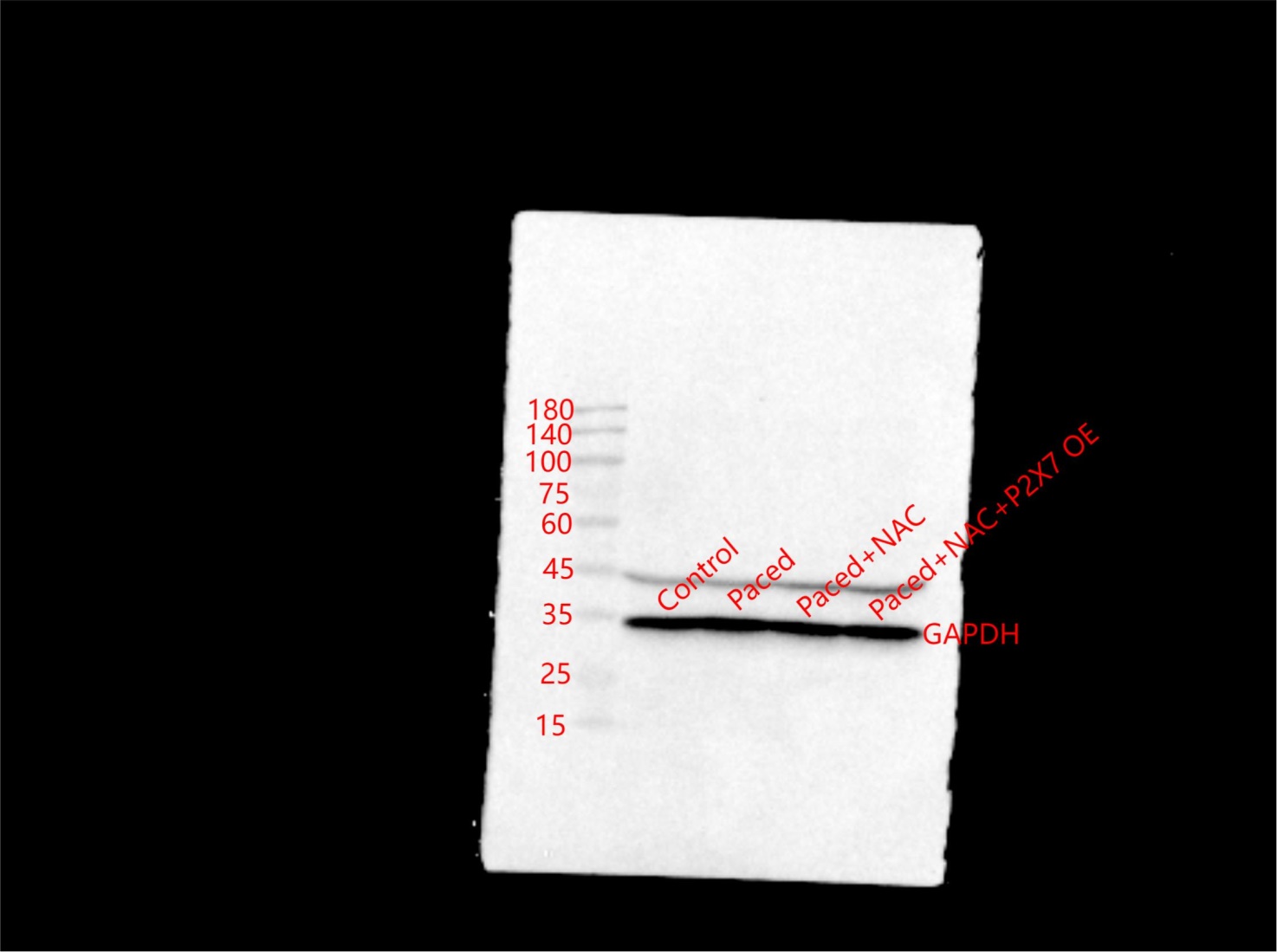


Figure 5E-7


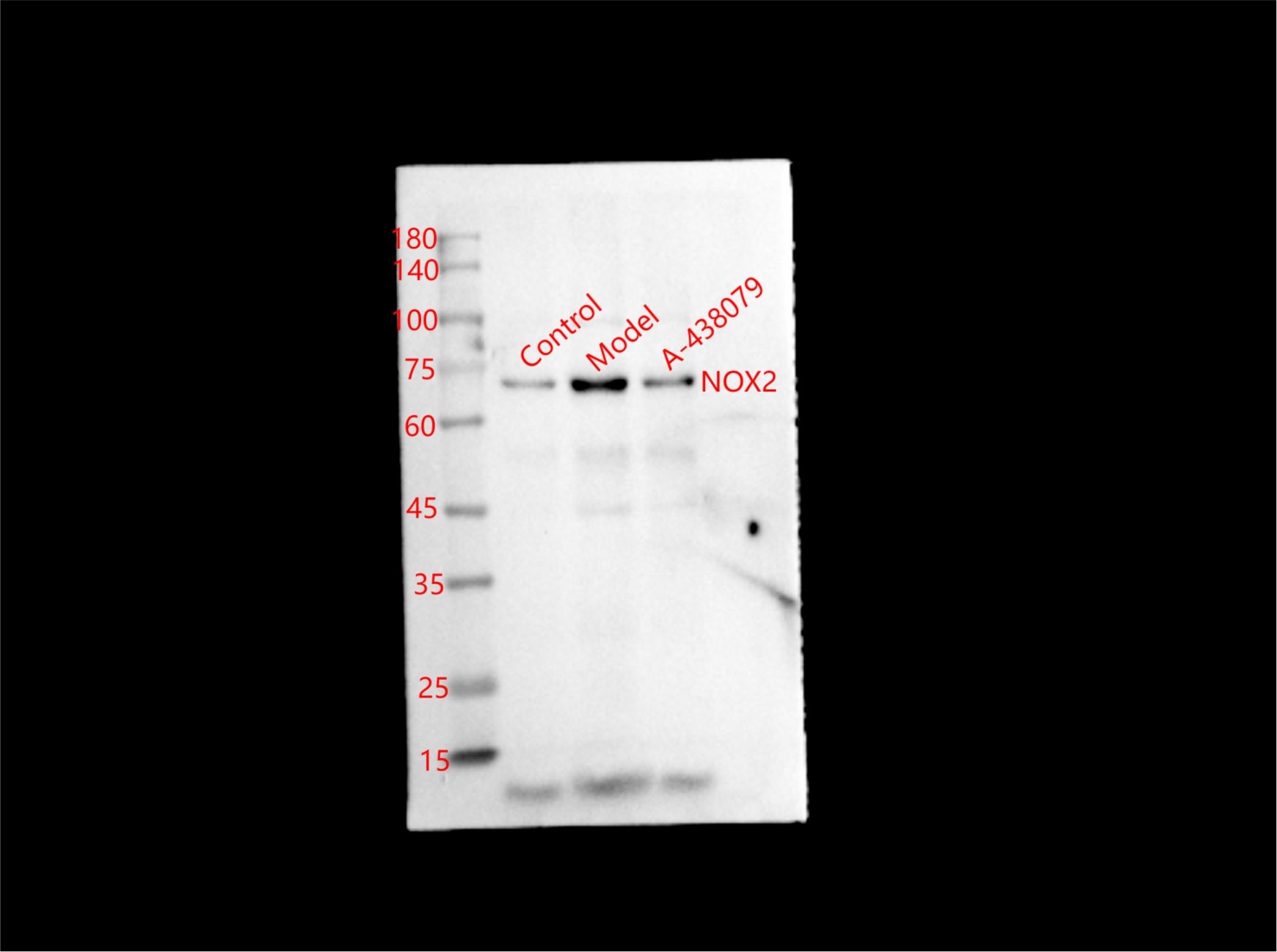


Figure 7C-1


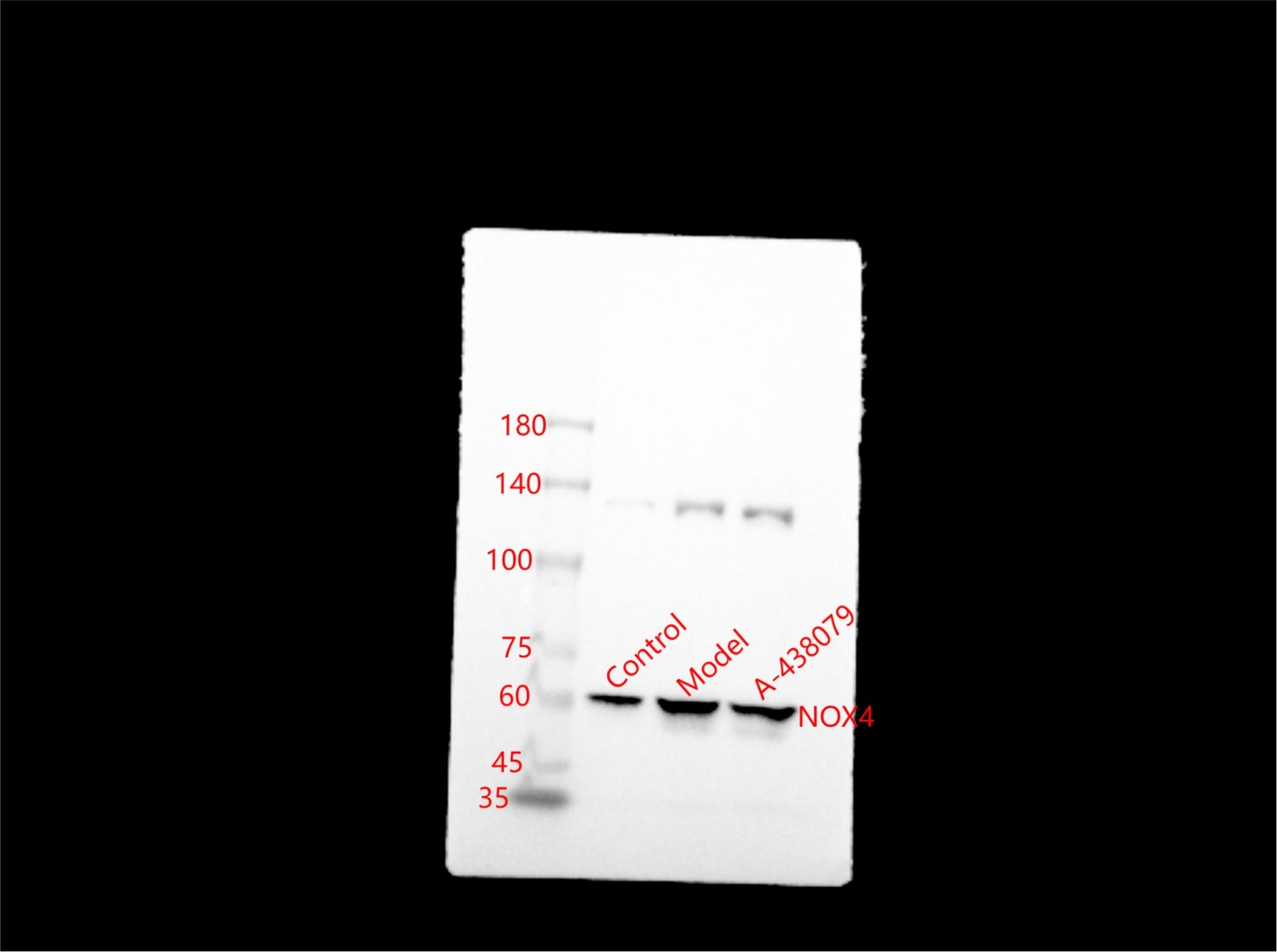


Figure 7C-2


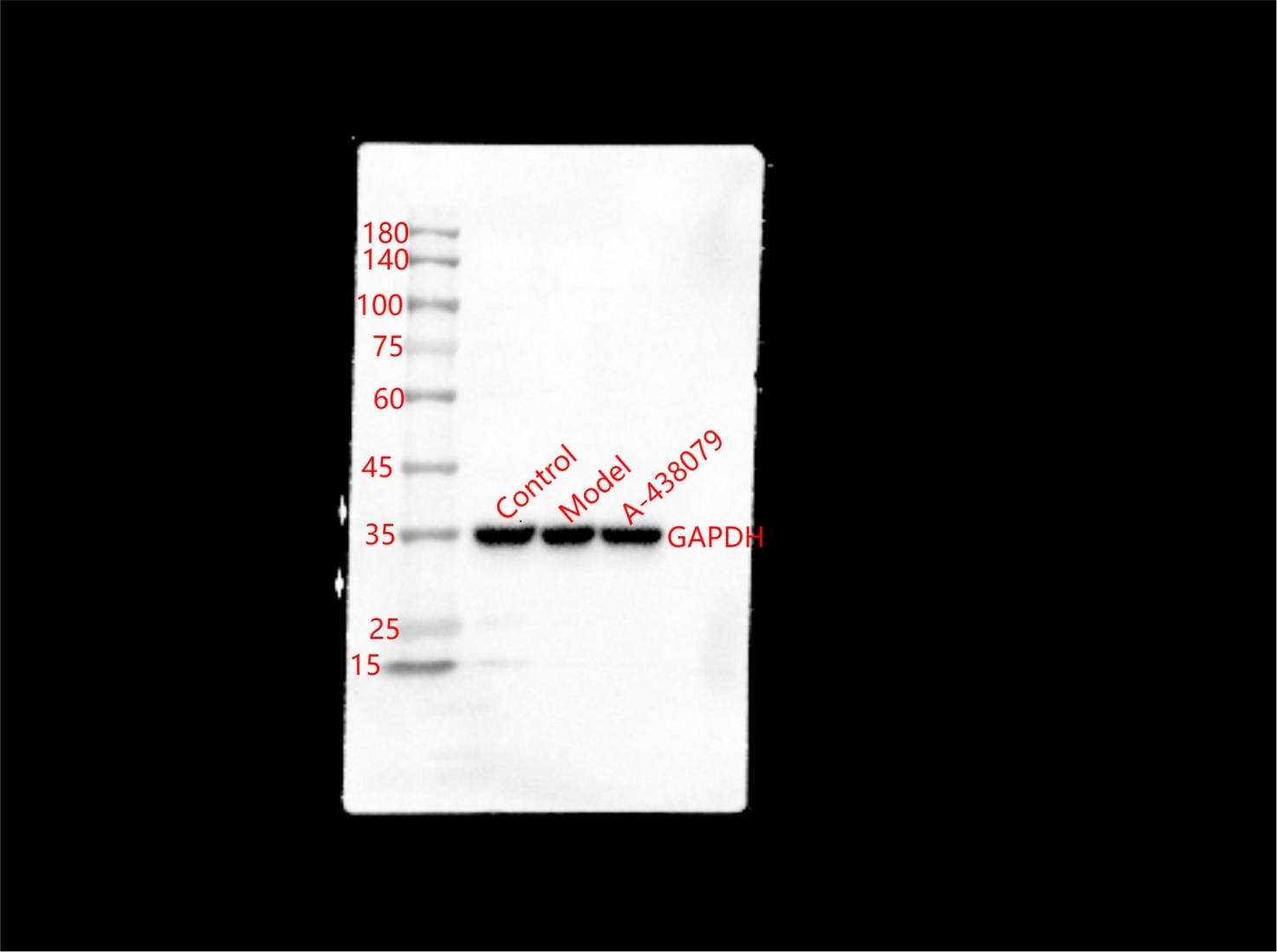


Figure 7C-3


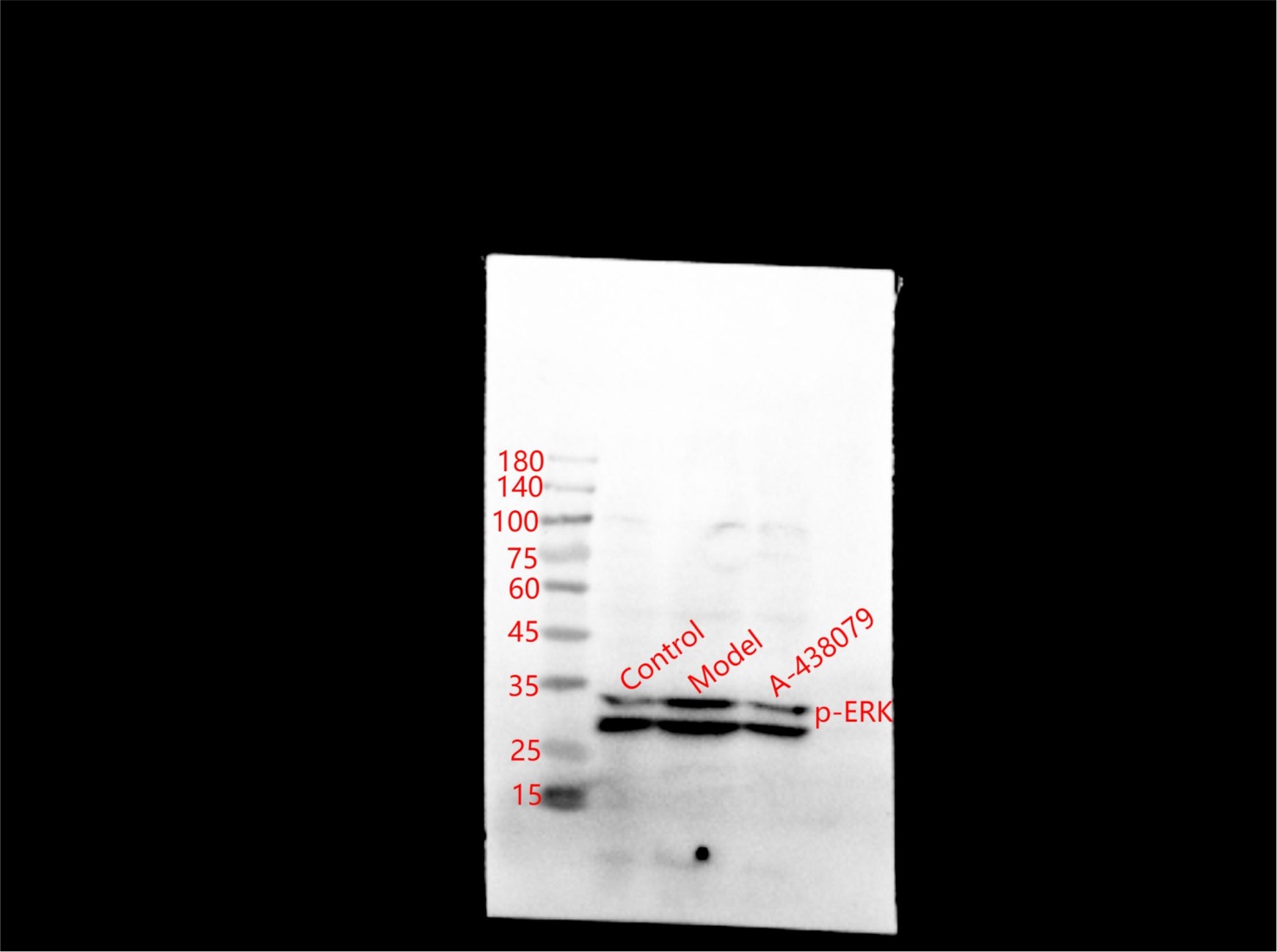


Figure 7E-1


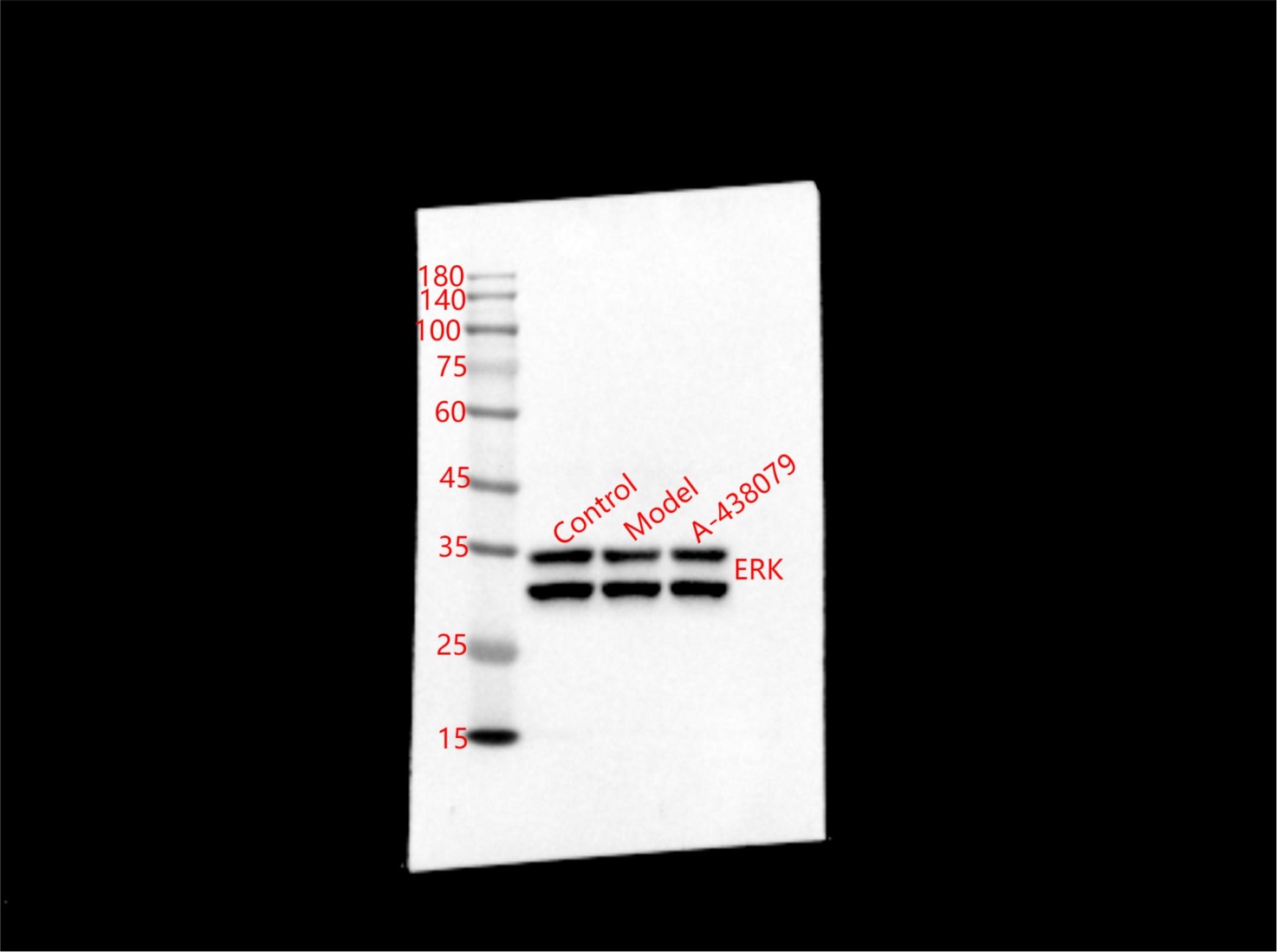


Figure 7E-2


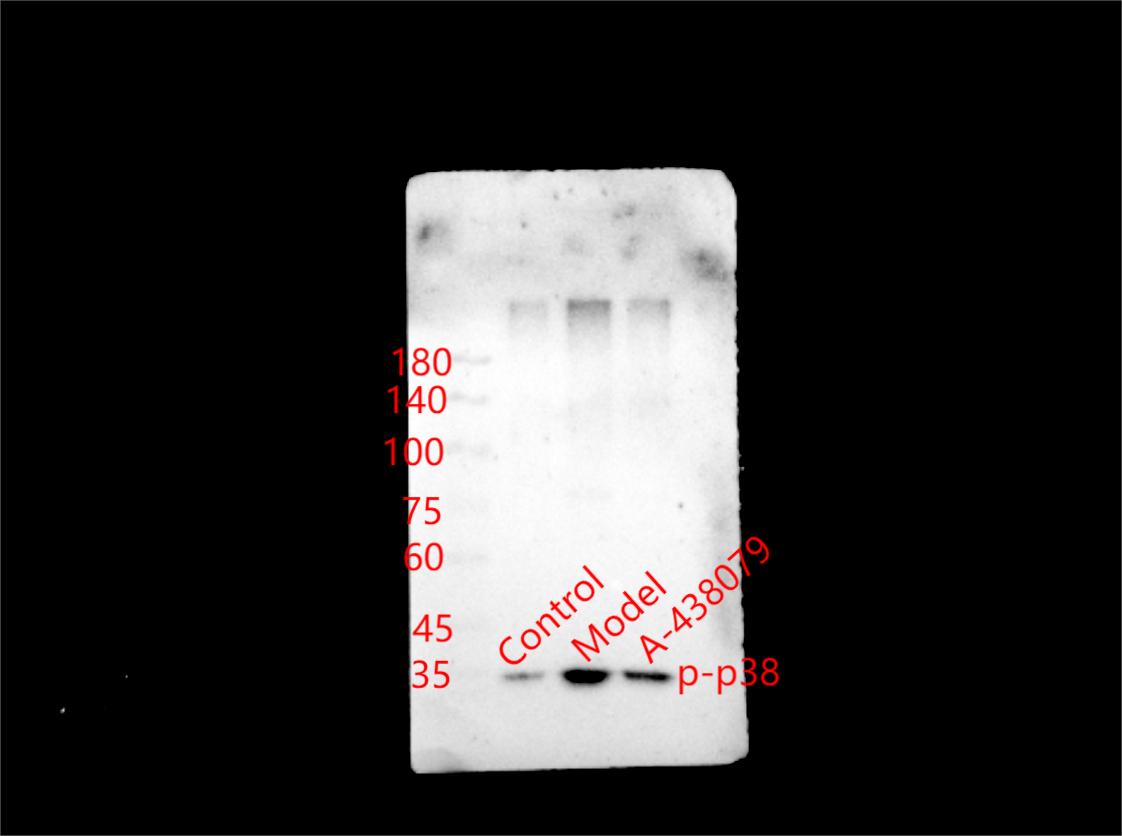


Figure 7E-3


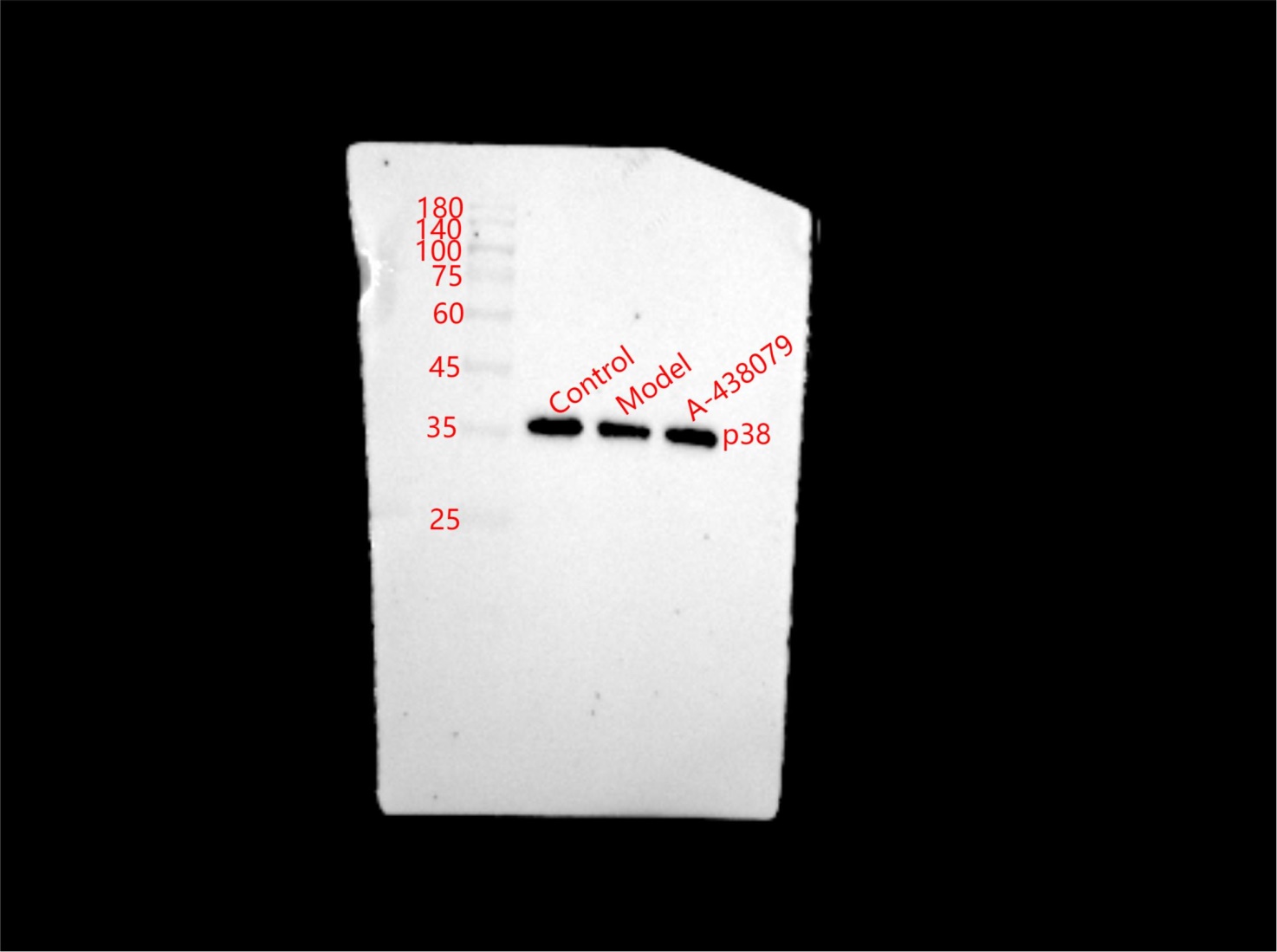


Figure 7E-4


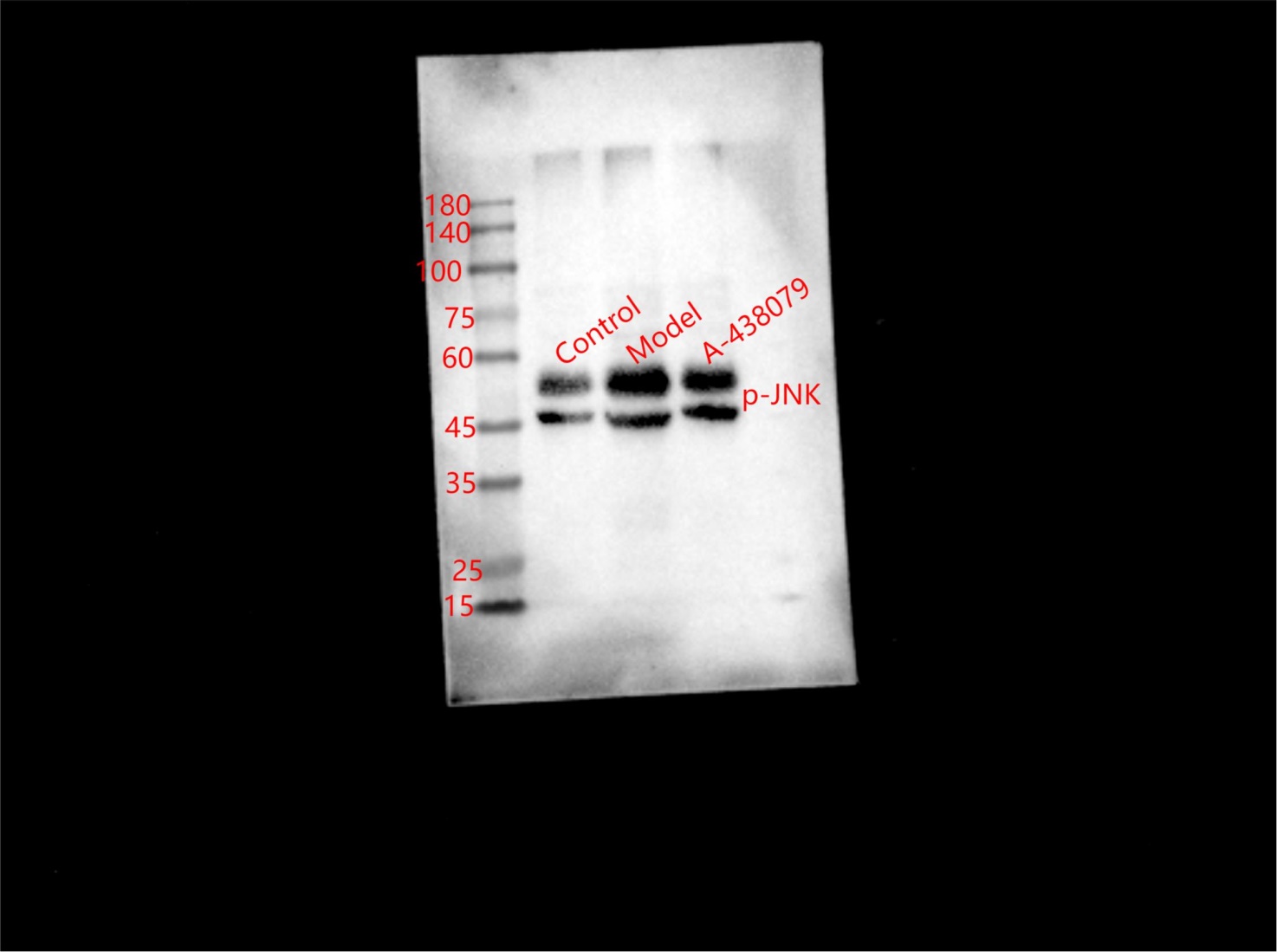


Figure 7E-5


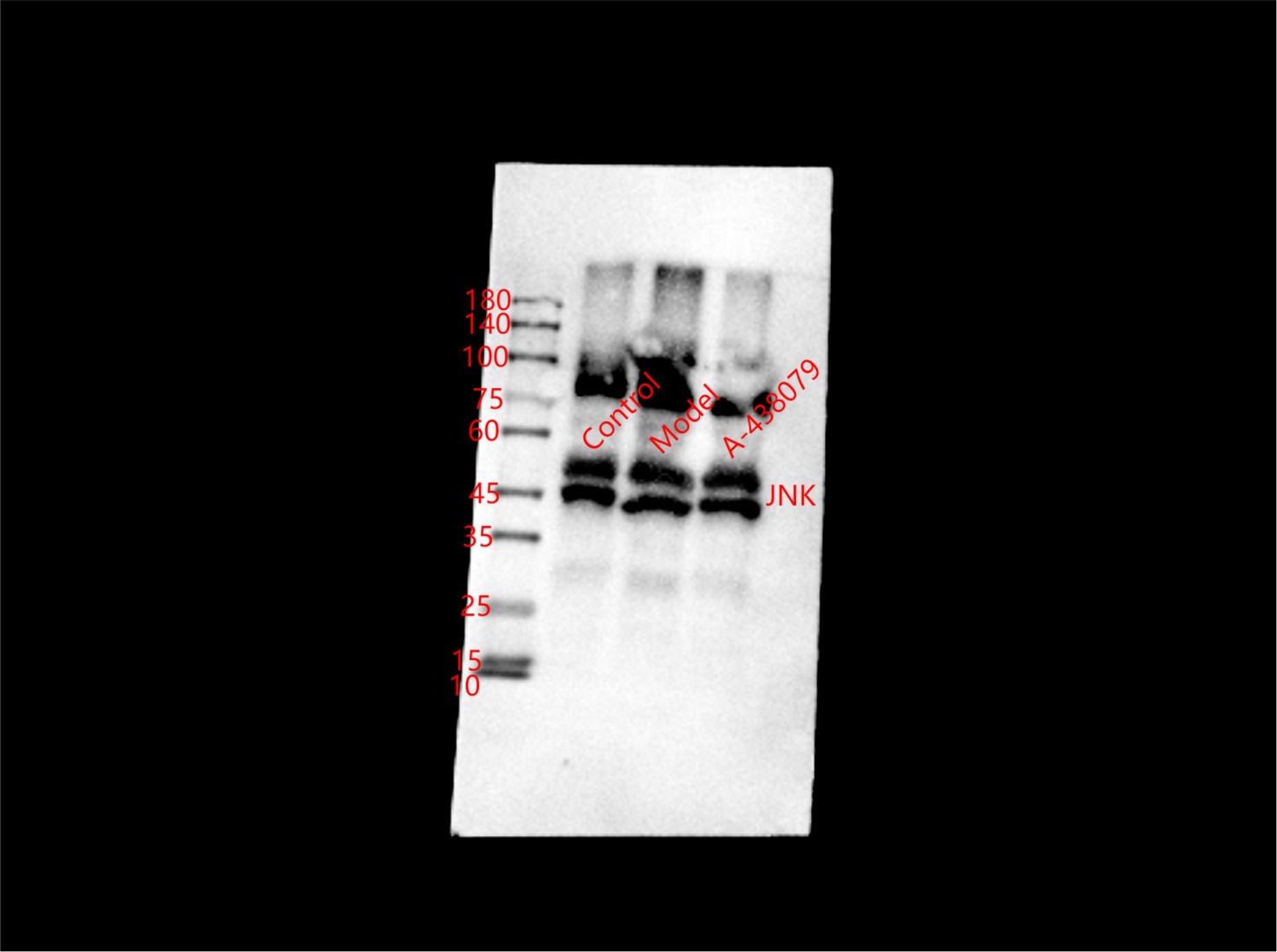


Figure 7E-6


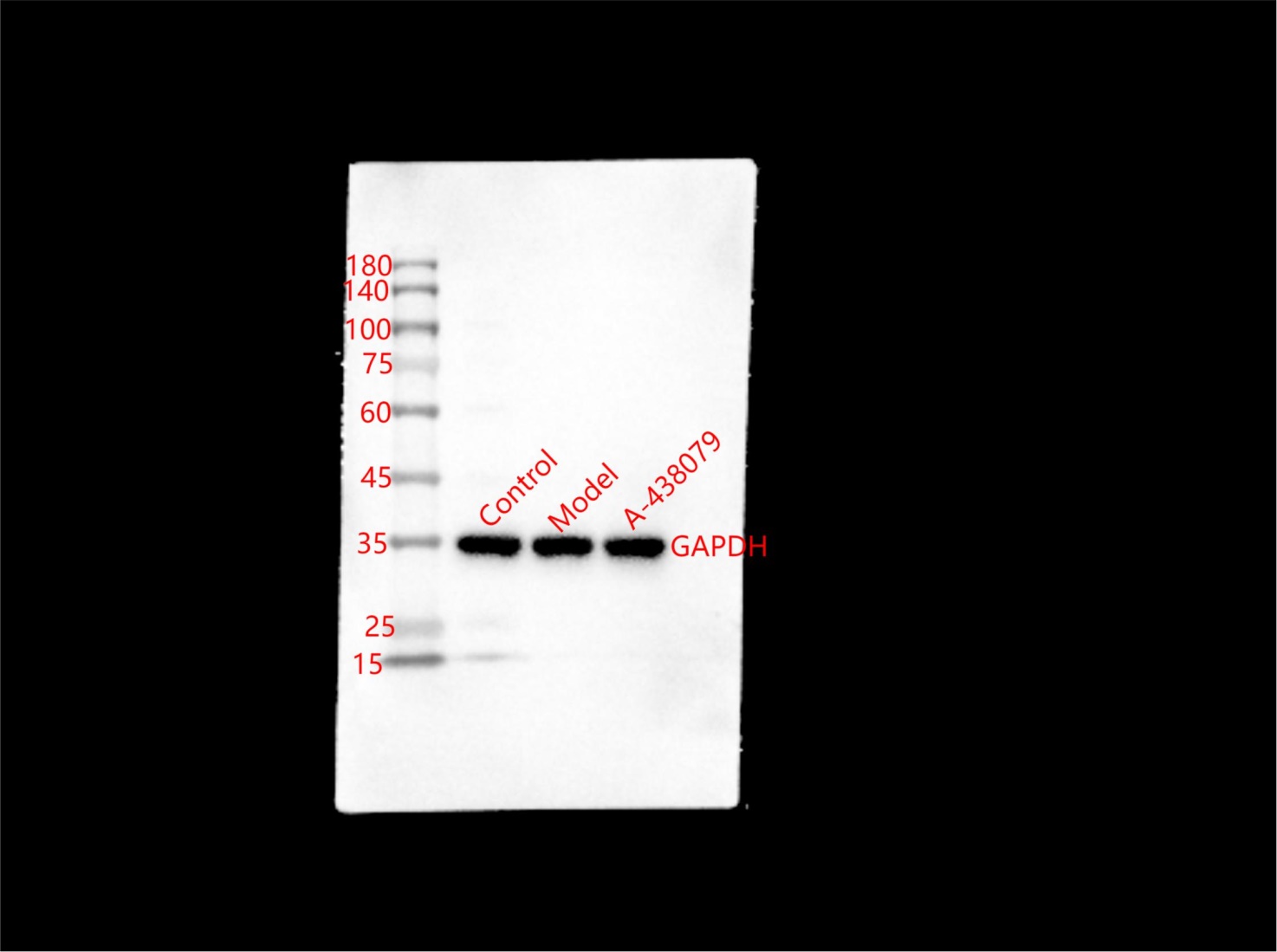


Figure 7E-7


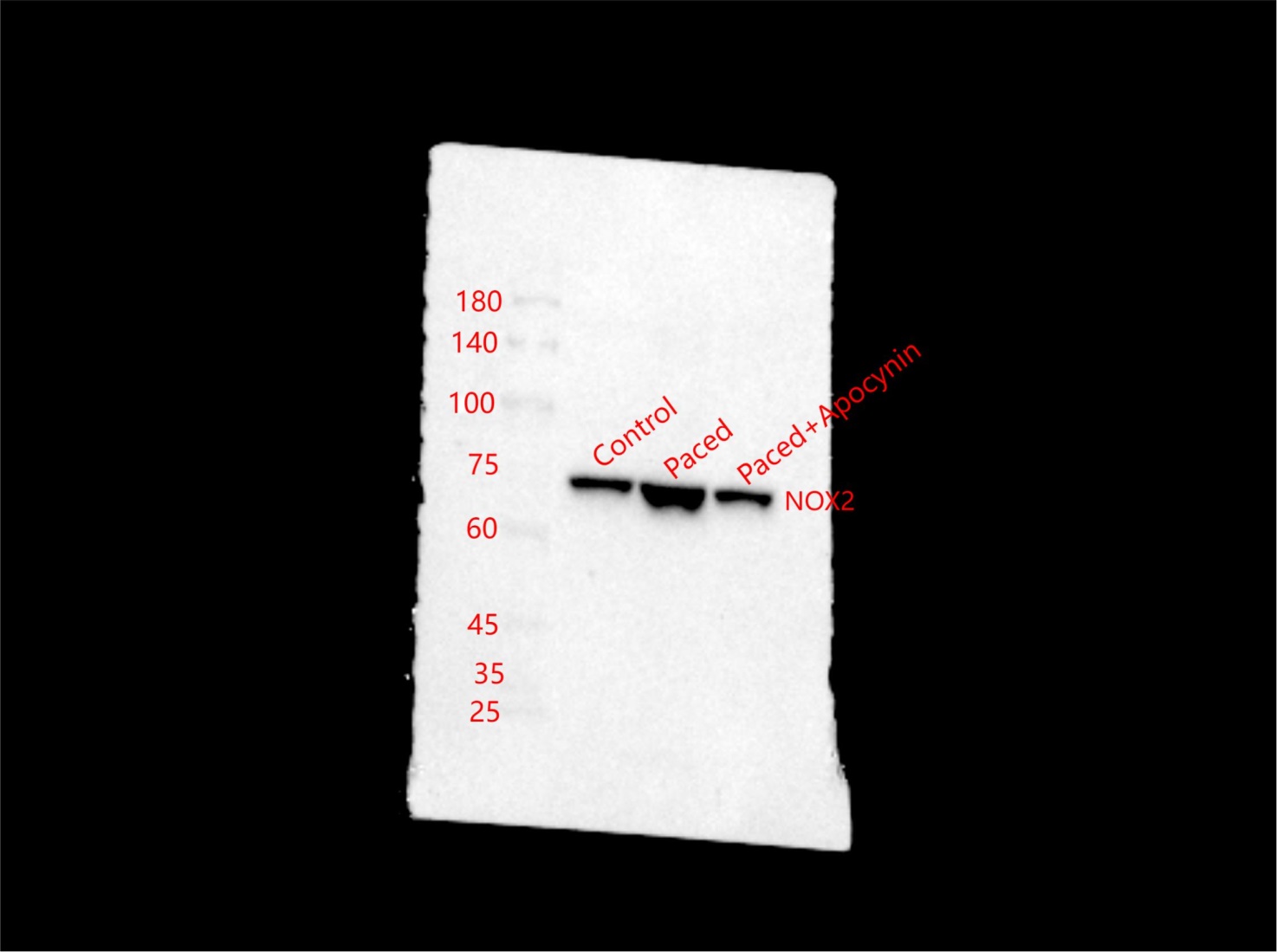


Figure S1C-1


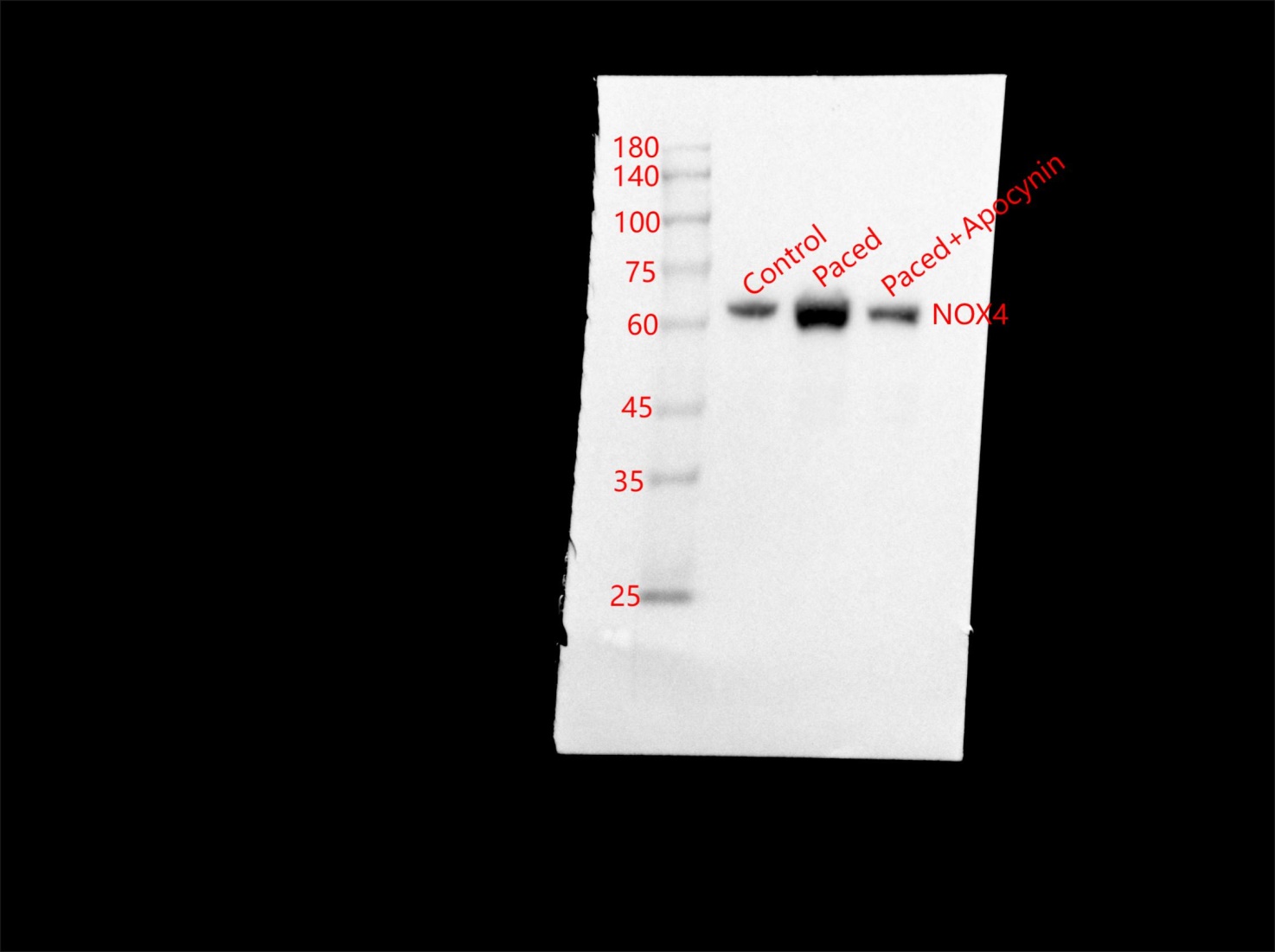


Figure S1C-2


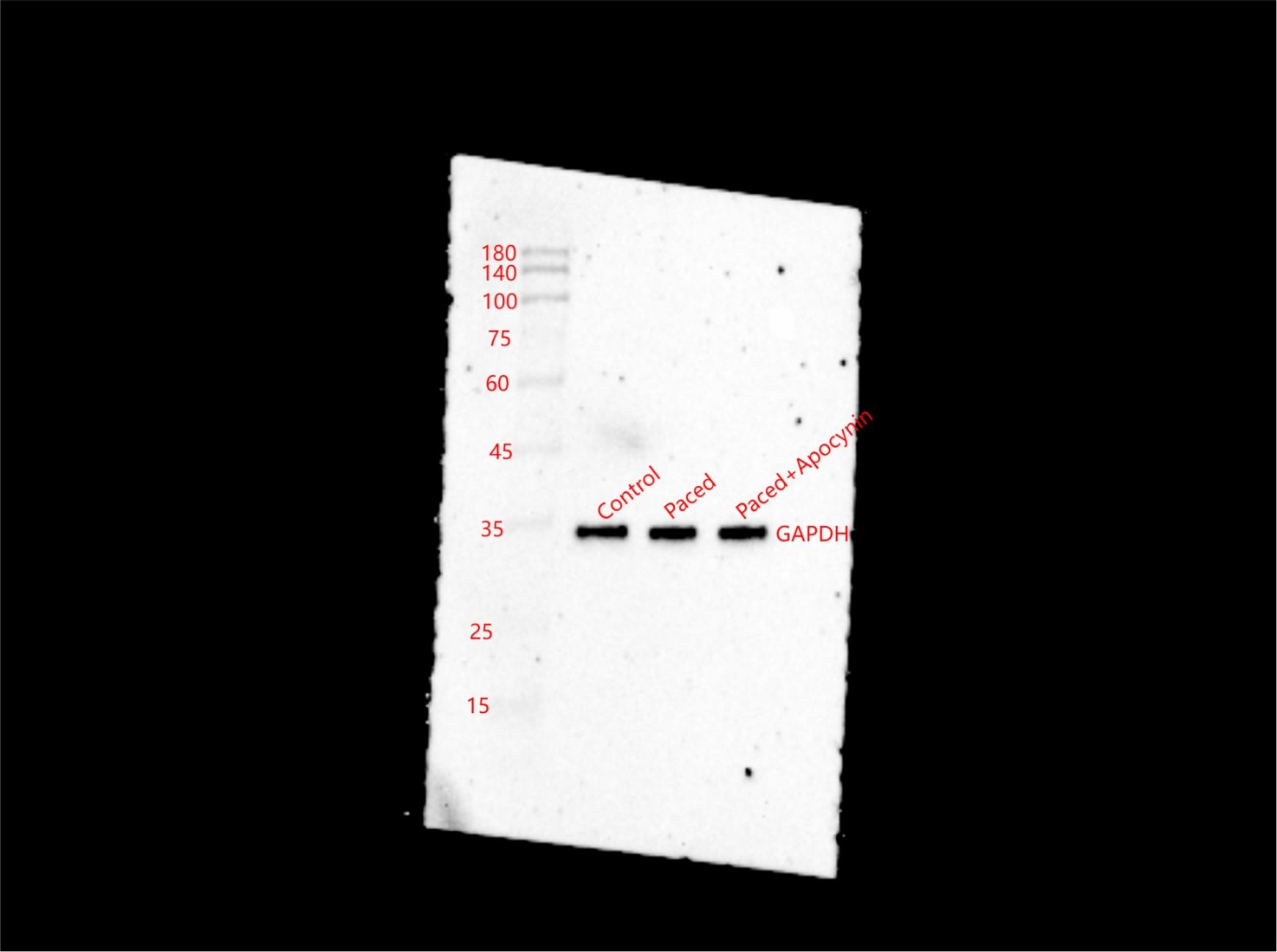


Figure S1C-3


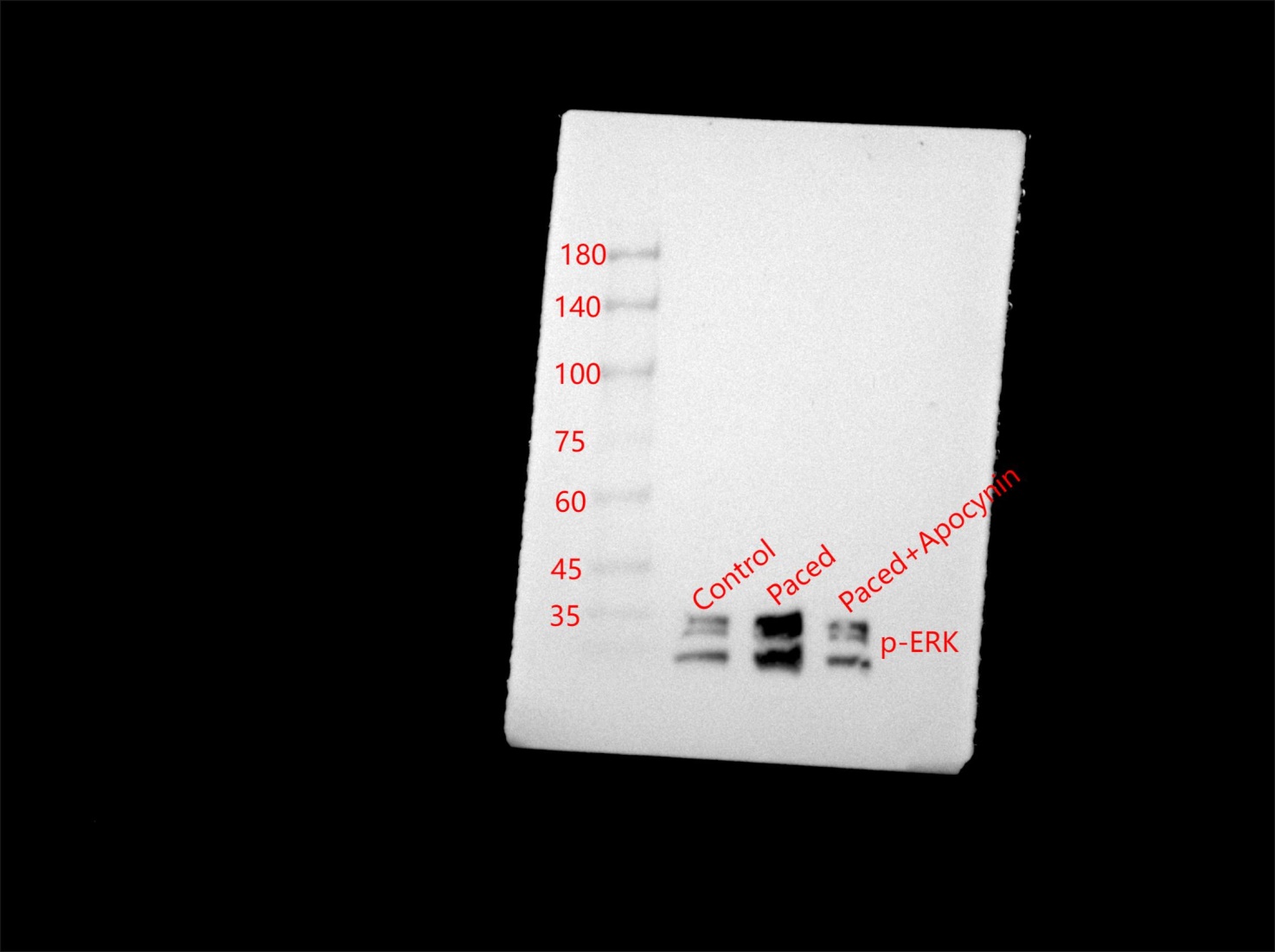


Figure S1E-1


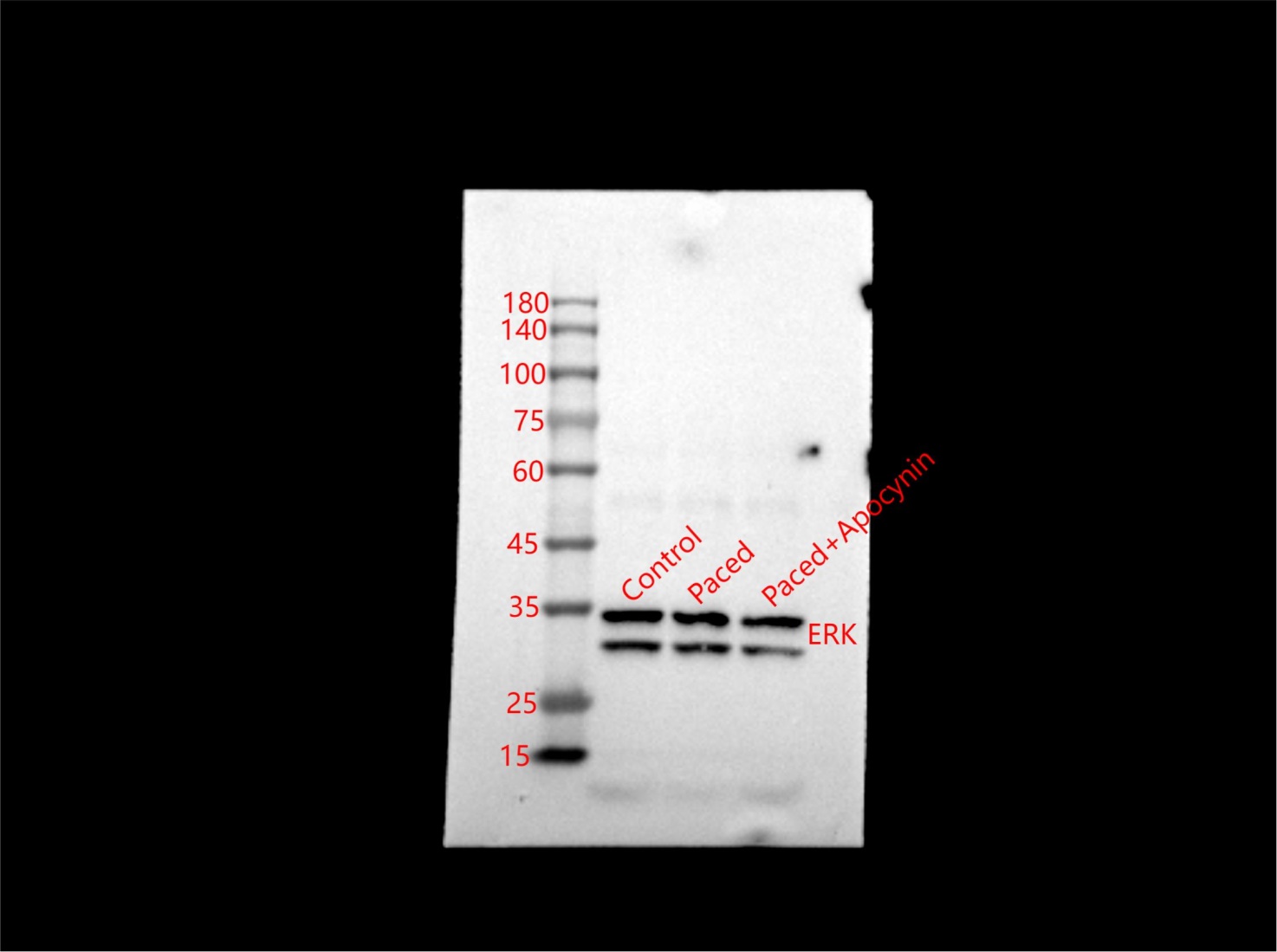


Figure S1E-2


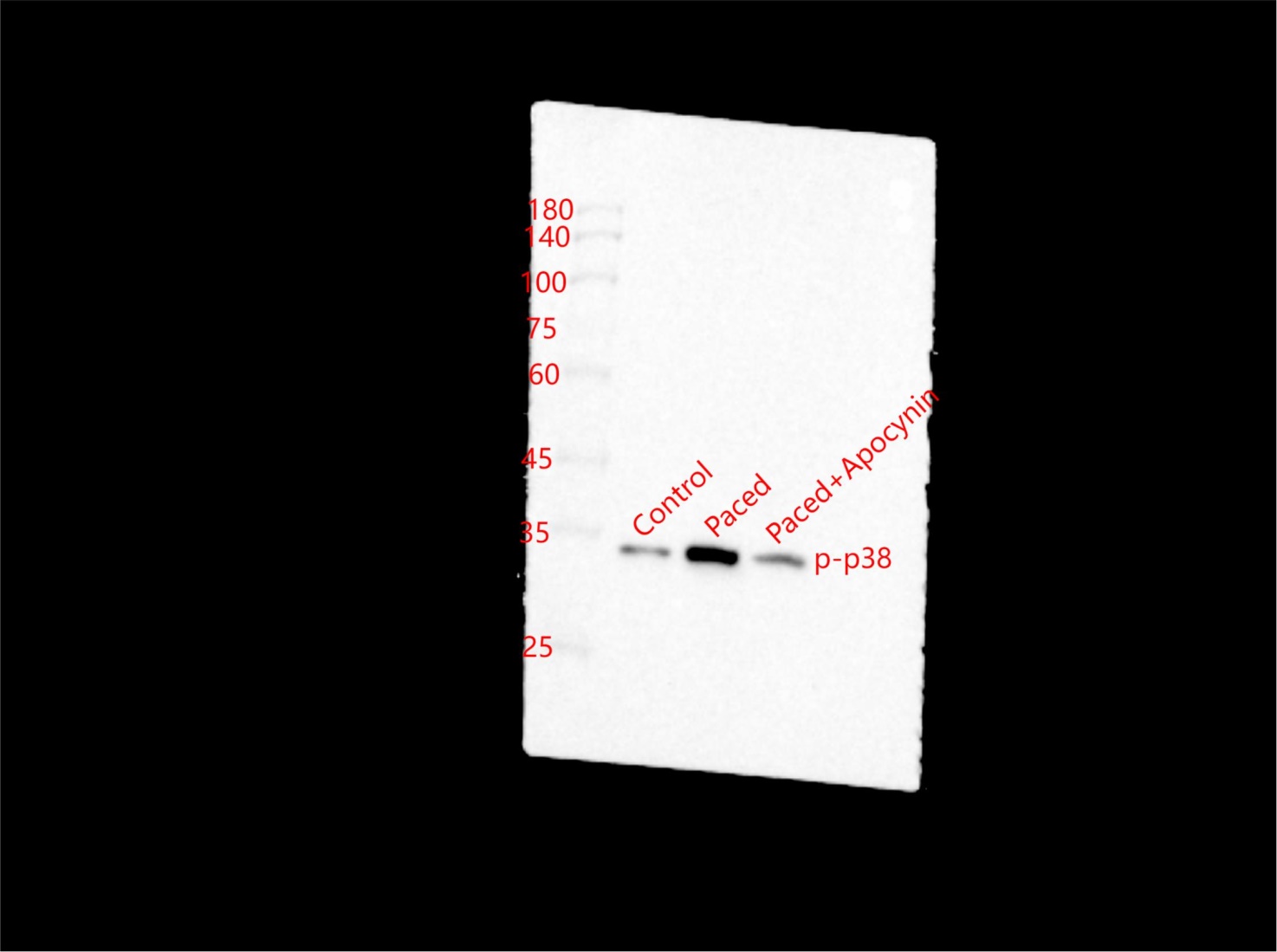


Figure S1E-3


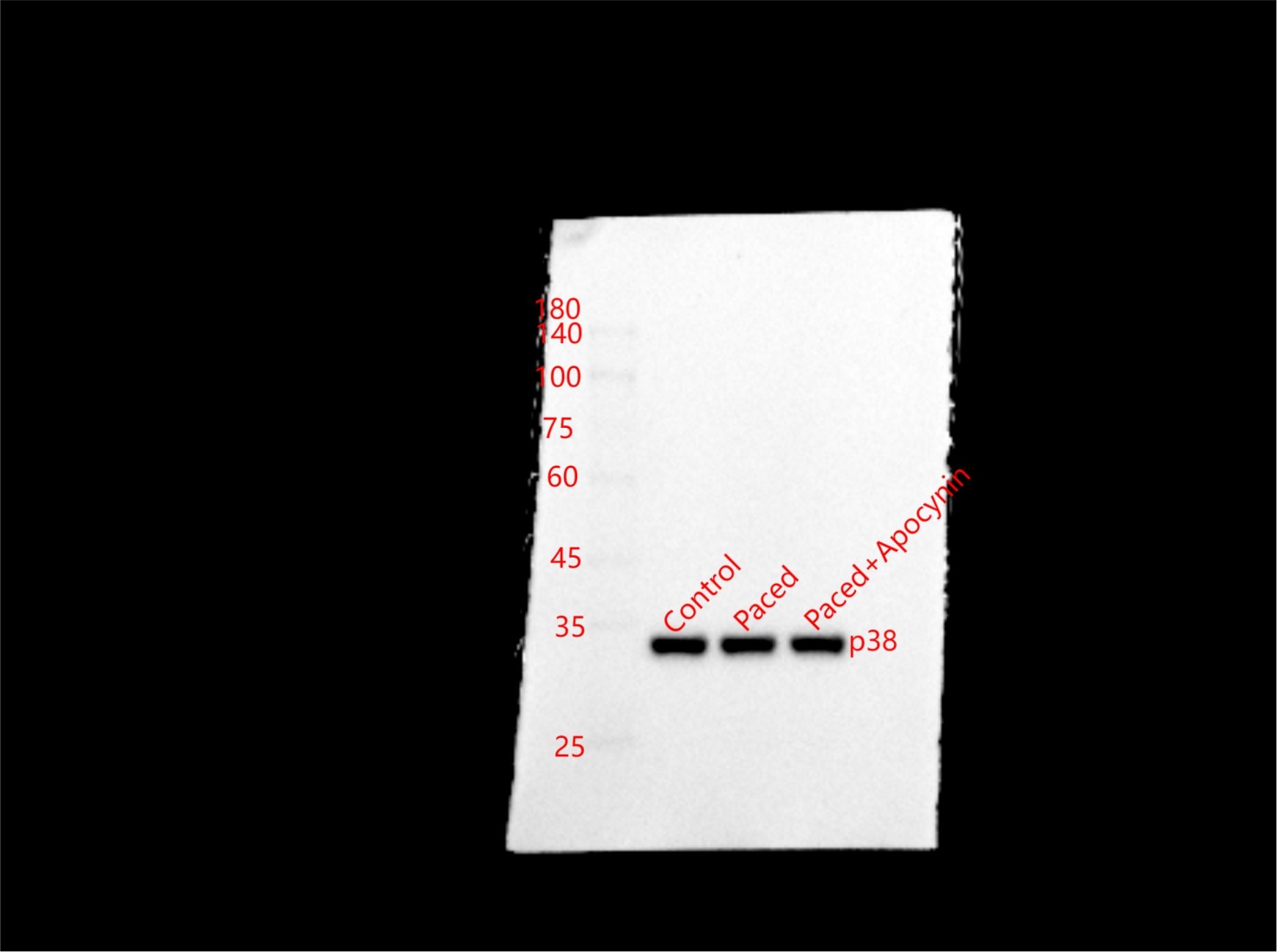


Figure S1E-4


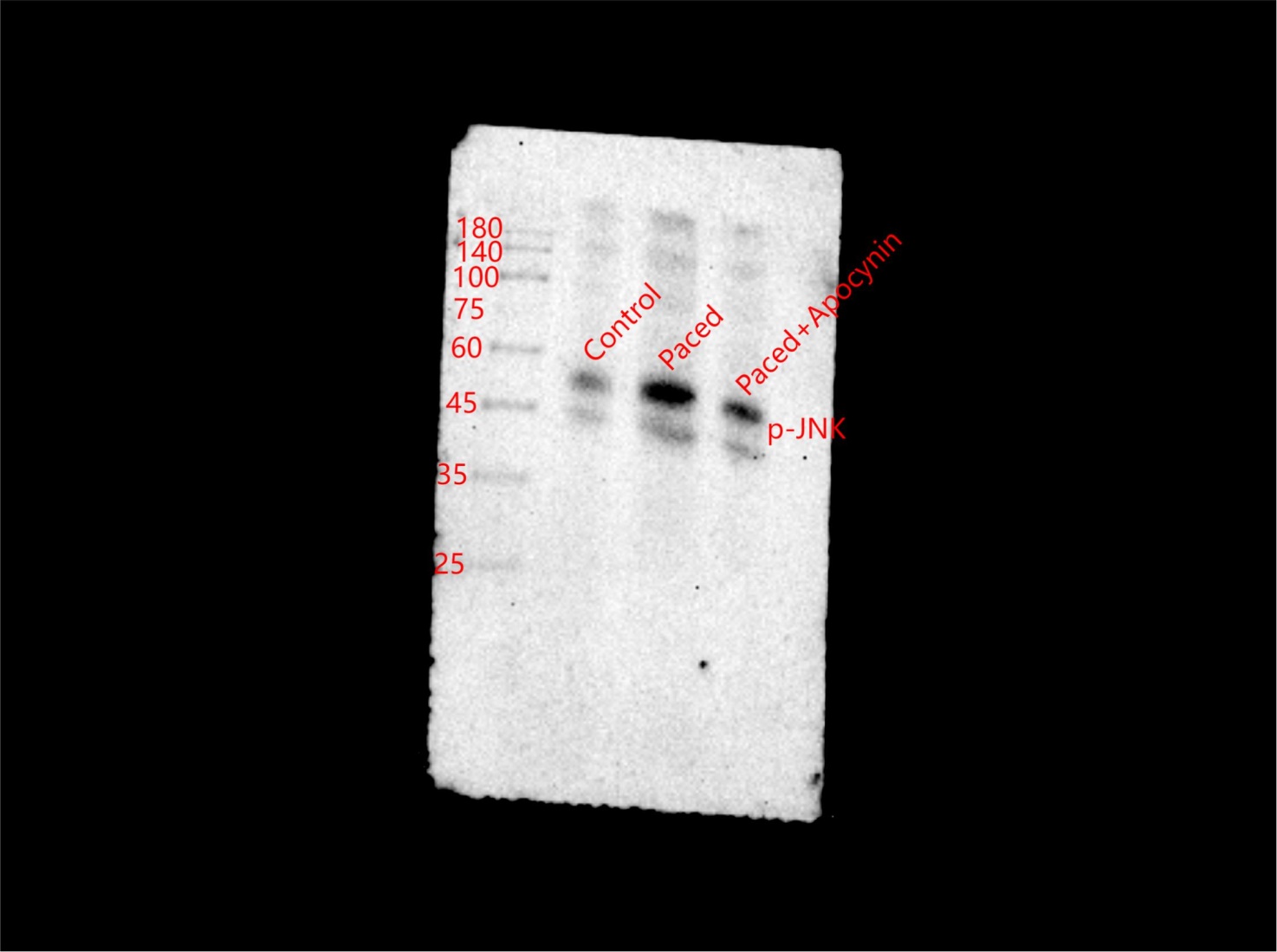


Figure S1E-5


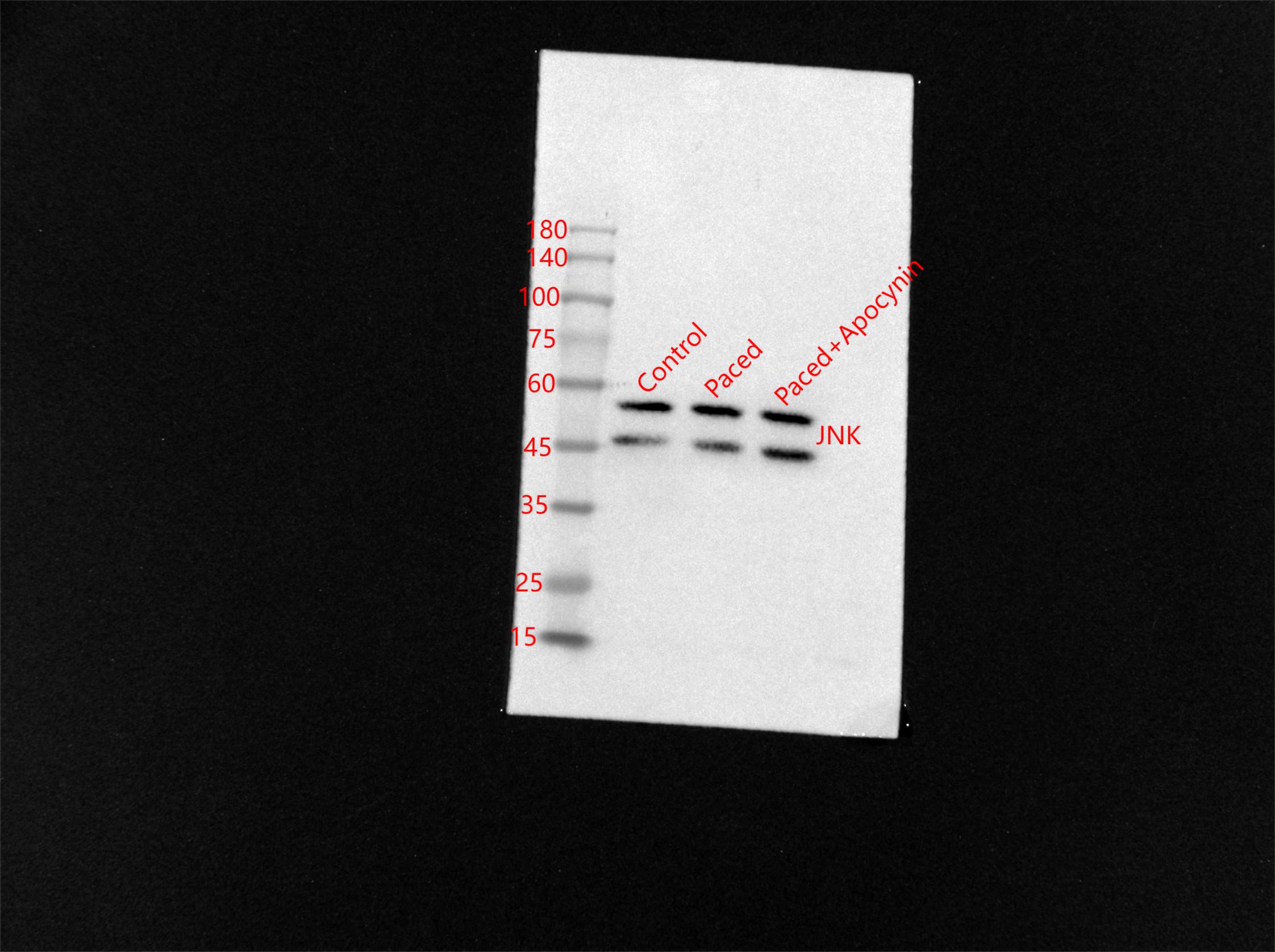


Figure S1E-6


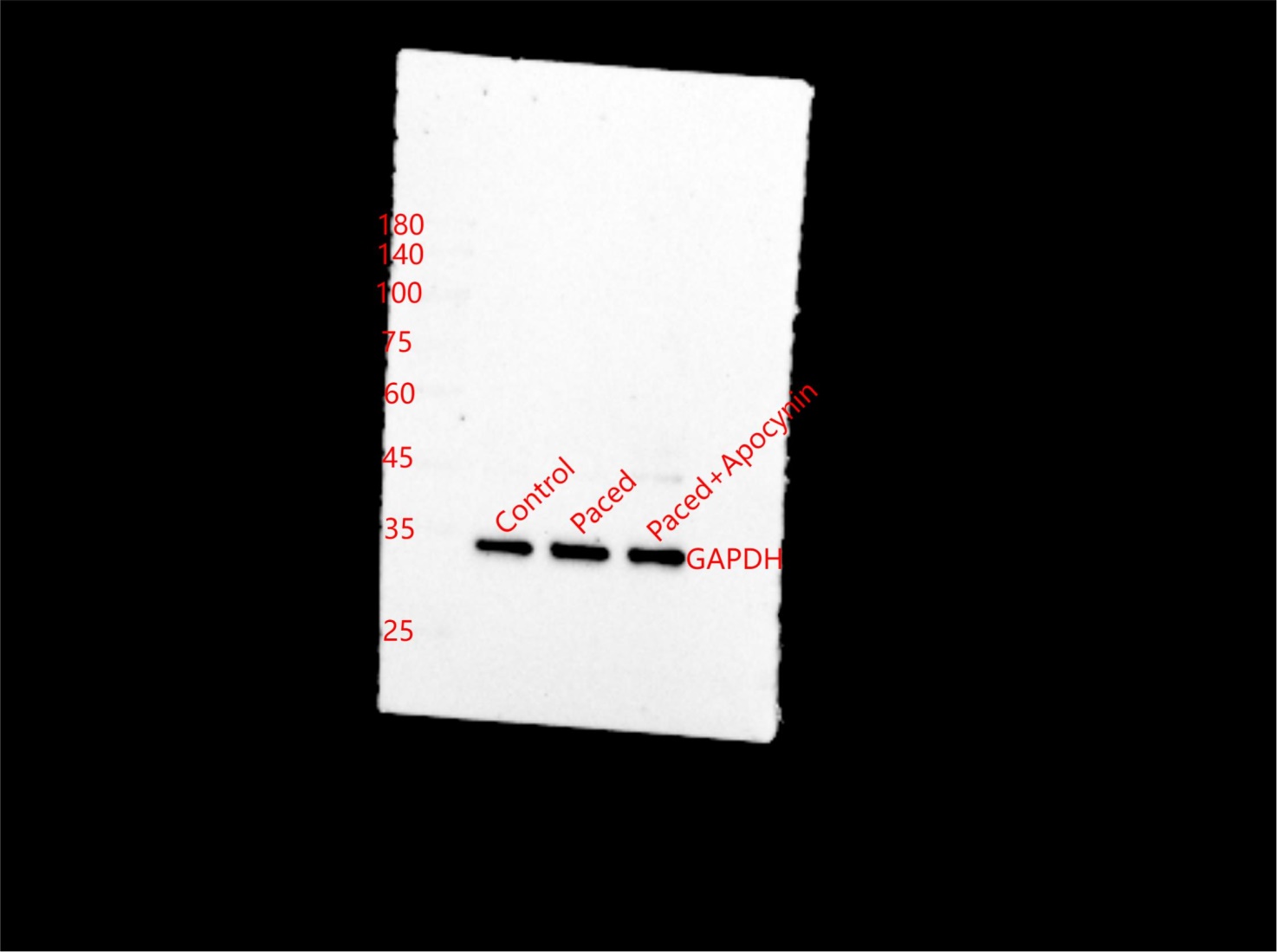


Figure S1E-7
