# Supplementary material for: P2X7 receptors promote atrial remodeling and atrial fibrillation susceptibility via reactive oxygen species‐mediated mitogen‐activated protein kinase signaling activation
Source: J Cell Commun Signal. 2026 Apr 30;20(2):e70071. doi: 10.1002/ccs3.70071 (PMC13130150; doi:10.1002/ccs3.70071)
Supplement: Supplementary file 2 — Figure S1 [file CCS3-20-e70071-s002.docx]

**
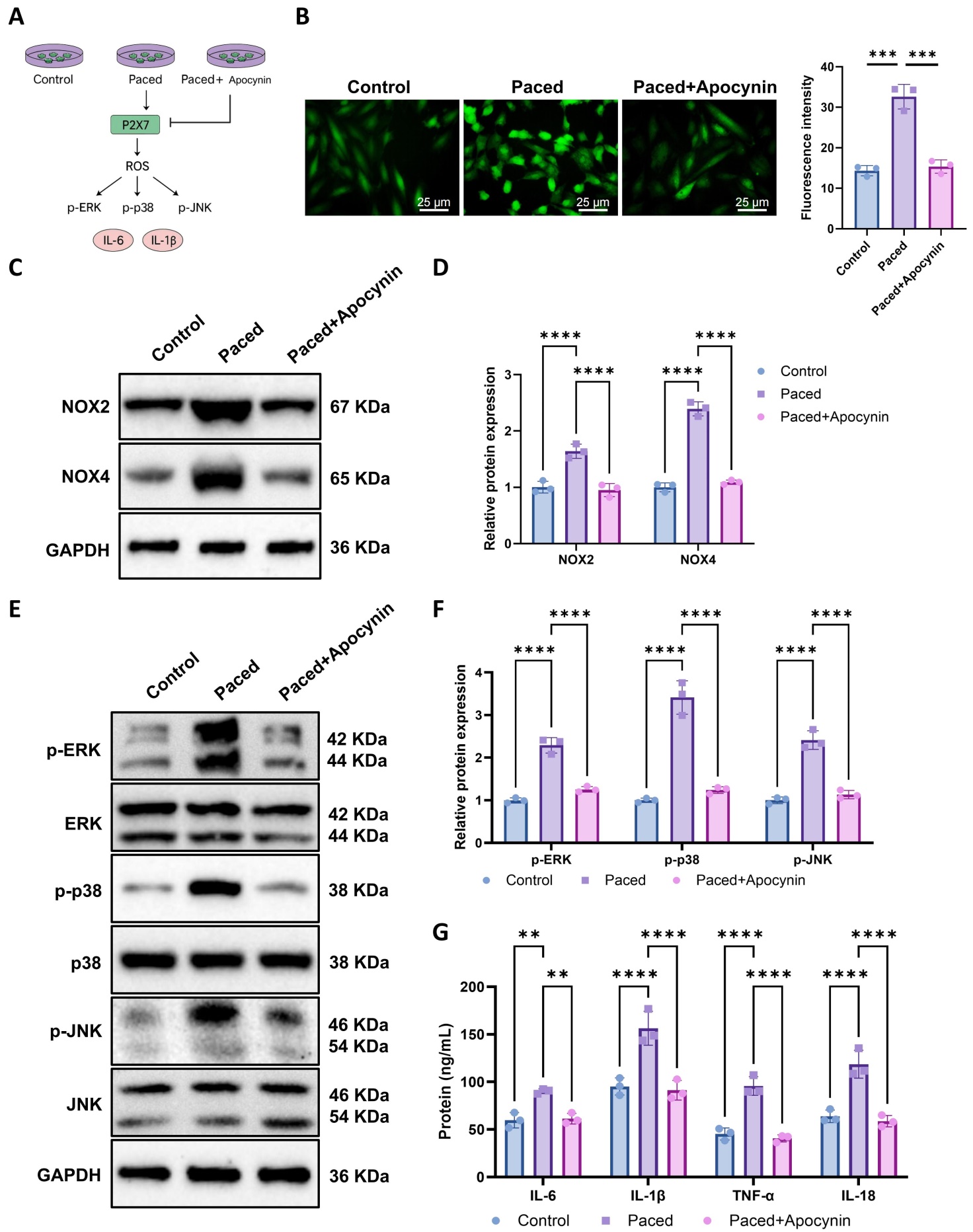
**

**Figure S1. Apocynin markedly attenuates Oxidative Stress and MAPK Pathway Activation**

Note: (A) Schematic illustration of the experimental workflow; (B) Representative fluorescence images of intracellular ROS levels detected by **DCFH-DA staining** in HL-1 cells from the **Control**, **Paced**, and **Paced+apocynin** groups, along with quantitative analysis of ROS fluorescence intensity. **Scale bar: 50 μm;** (C-F) **Western blot analysis** of protein expression and the corresponding **densitometric quantification;** (G) **ELISA-based quantification** of inflammatory cytokines **IL-6, IL-1β, TNF-α, and IL-18** in cell culture supernatants. All cell-based experiments were performed with **three independent biological replicates (n = 3)**. ***p* < 0.01; ****p* < 0.001, *****p* < 0.0001.
